# Unraveling the dual roles of MICAL2 in skeletal muscle physiology and fusion negative rhabdomyosarcoma tumor progression

**DOI:** 10.1038/s41419-026-09029-w

**Published:** 2026-07-07

**Authors:** Ashley Wang, Nefele Giarratana, Simen Cassiman, Martina Biglietto, Lorenza Rinvenuto, Laura Yedigaryan, Enrico Pozzo, Vittoria Marini, Maxime Smits, Alvaro Cortes Calabuig, Khaled Mirzaei, Pedro Magalhães, Zhongpeng Ge, Margalida I. Campaner Socias, Jianhao Zhang, Alessandro Fanzani, Rik Gijsbers, Maurilio Sampaolesi

**Affiliations:** 1 Translational Cardiomyology Laboratory, Stem Cell and Developmental Biology, Department of Development and Regeneration, KU Leuven, Herestraat 49, 3000 Leuven, Belgium; 2 ESAT-PSI, KU Leuven, 3000 Leuven, Belgium; 3 Histology and Medical Embryology Unit, Department of Anatomy, Histology, Forensic Medicine and Orthopedics, Sapienza University of Rome, Rome, Italy; 4 ADVANTAGE Group - Advanced Disease Modelling, Targeted Drug Discovery, and Gene Therapy, Department of Pharmaceutical and Pharmacological Sciences, KU Leuven, 3000 Leuven, Belgium; 5 Leuven Viral Vector Core (LVVC), KU Leuven, 3000 Leuven, Belgium; 6 KU Leuven Genomics Core, Department of Human Genetics, KU Leuven, 3000 Leuven, Belgium; 7 Laboratory of Applied Mass Spectrometry, Department of Cellular and Molecular Medicine, KU Leuven, Leuven, Belgium; 8 VIB-KU Leuven Center for Neuroscience, Proteomics Technology Unit, Herestraat 49, 3000 Leuven, Belgium; 9 Department of Molecular and Translational Medicine, University of Brescia, Brescia, Italy

**Keywords:** Paediatric cancer, RNAi

## Abstract

Rhabdomyosarcoma (RMS) is a prevalent soft tissue sarcoma in pediatric populations characterized by dysregulated myogenesis and limited treatment options. Notably, fusion negative RMS (FN-RMS) constitutes the predominant subtype in pediatric patients and commonly harbors RAS mutations, resulting in uncontrolled cell proliferation and invasion. To date, the precise molecular determinants underlying FN-RMS tumor biology remain poorly understood, but there is increasing evidence implicating aberrant cytoskeletal remodeling in promoting malignant phenotypes. In this study, we present MICAL2, a flavin monooxygenase involved in regulating actin dynamics, as a novel driver of FN-RMS tumor progression. We first revealed elevated MICAL2 expression in FN-RMS cell lines, which was consistent with prior reports in other malignancies, then demonstrated that its silencing altered tumor cell behaviors. By knocking down MICAL2 using RNA interference strategies, murine and human FN-RMS cells displayed reduced proliferative and migratory capacities in vitro, which translated to attenuated primary tumor growth, inhibited metastasis formation, and improved functional performances in vivo. Furthermore, multi-omics analyses highlighted the molecular changes associated with MICAL2 silencing, including simultaneous suppression of oncogenic programs and enhancement of muscle integrity and oxidative metabolic function. Collectively, these data highlight the duality of MICAL2 as a cytoskeletal regulator whose dysregulation may disrupt myogenic identity and promote FN-RMS pathogenesis.

## Introduction

Skeletal muscles, which represent around 30–40% of the adult body mass, constitute a complex and heterogeneous organ system that is responsible for many different critical physiological functions [1, 2]. Muscle tissue possesses the capacity to respond to physiological changes, with intricate biological processes intricately regulating adult myogenesis [3, 4]. However, pathologies may also emerge during muscle development, giving rise to neoplasms such as rhabdomyosarcoma (RMS), the most prevalent soft tissue sarcoma that accounts for almost 5% of pediatric cancers [5]. RMS is characterized by dysregulated myogenesis arising from unsuccessful terminal differentiation and represents a paradox where cells express myogenic regulatory factors MYOD and MYOGENIN but are unable to fully commit to a mature muscle fate [6]. Patient outcomes depends heavily on the subtype, primary tumor size, presence of metastasis, and treatment options, but even with the introduction of multimodal therapy, survival rates of patients with metastatic disease remain below 30%, as it is unable to restore intrinsic myogenic propensity [7]. Notably, since fusion protein status has emerged as the most important prognostic factor in RMS, the two main subtypes of fusion positive (FP-RMS) and fusion negative (FN-RMS) have replaced conventional classification systems, with the latter being the most common [8, 9].

In contrast to FP-RMS, which is defined by the presence of *PAX3*-*FOXO1* or *PAX7*-*FOXO1* fusion genes, FN-RMS lacks these chimeric proteins and instead, harbors a variety of genetic alterations [7, 10]. Particularly, mutations in RAS family genes, including KRAS, NRAS, and HRAS, are regarded as key drivers of FN-RMS and account for a large proportion of these cases [11, 12]. A major consequence of hyperactive RAS signaling is perturbed cytoskeletal remodeling, involving alteration of actin turnover, disruption of microtubule dynamics, and destabilization of intercellular junctions, which collectively promote uncontrolled cell proliferation and invasive behavior [13–16]. However, the specific regulators of cytoskeletal dynamics that mediate RAS-driven tumor aggressiveness and suppression of myogenic commitment in FN-RMS have not been elucidated. Within this context, we have identified Microtubule Associated Monooxygenase, Calponin And LIM domain containing protein 2 (MICAL2) as a potential molecular contributor to FN-RMS pathogenesis. This member of the MICAL family of flavin monooxygenases plays an important role in regulating cytoskeletal dynamics via redox-mediated F-actin depolymerization [17, 18]. We recently discovered that although MICAL2 has been shown to be a marker of metastatic disease in several malignancies including gastric, breast, lung, and pancreatic cancers [19–22], it is in fact crucial for modulating myogenic differentiation and muscle regeneration under normal physiological conditions [23], highlighting its dual role in promoting myogenesis at homeostatic levels and driving malignancy upon overexpression. In this study, we provide the first evidence that MICAL2 is aberrantly expressed across both murine and human FN-RMS models and link its elevated abundance with features of tumor aggressiveness. Using microRNA (miR)-based short hairpin constructs, we showed that modulation of MICAL2 expression to more homeostatic levels significantly reduces tumor cell proliferation and migration in vitro, suppresses primary tumor growth, diminishes metastasis formation, and improves ambulation performances. Complementary bulk RNA-sequencing and mass spectrometry analyses of in vivo tumor tissues further revealed that MICAL2-silenced FN-RMS cells shifted toward transcriptional and proteomic signatures more closely resembling healthy muscle. Together, these findings identify MICAL2 as a significant contributor to FN-RMS tumor progression and provide initial evidence linking its aberrant expression to both oncogenic behavior and impaired myogenic commitment.

## Results

### MICAL2 knockdown reduces murine FN-RMS cell proliferation and migration

**Fig. 1:**
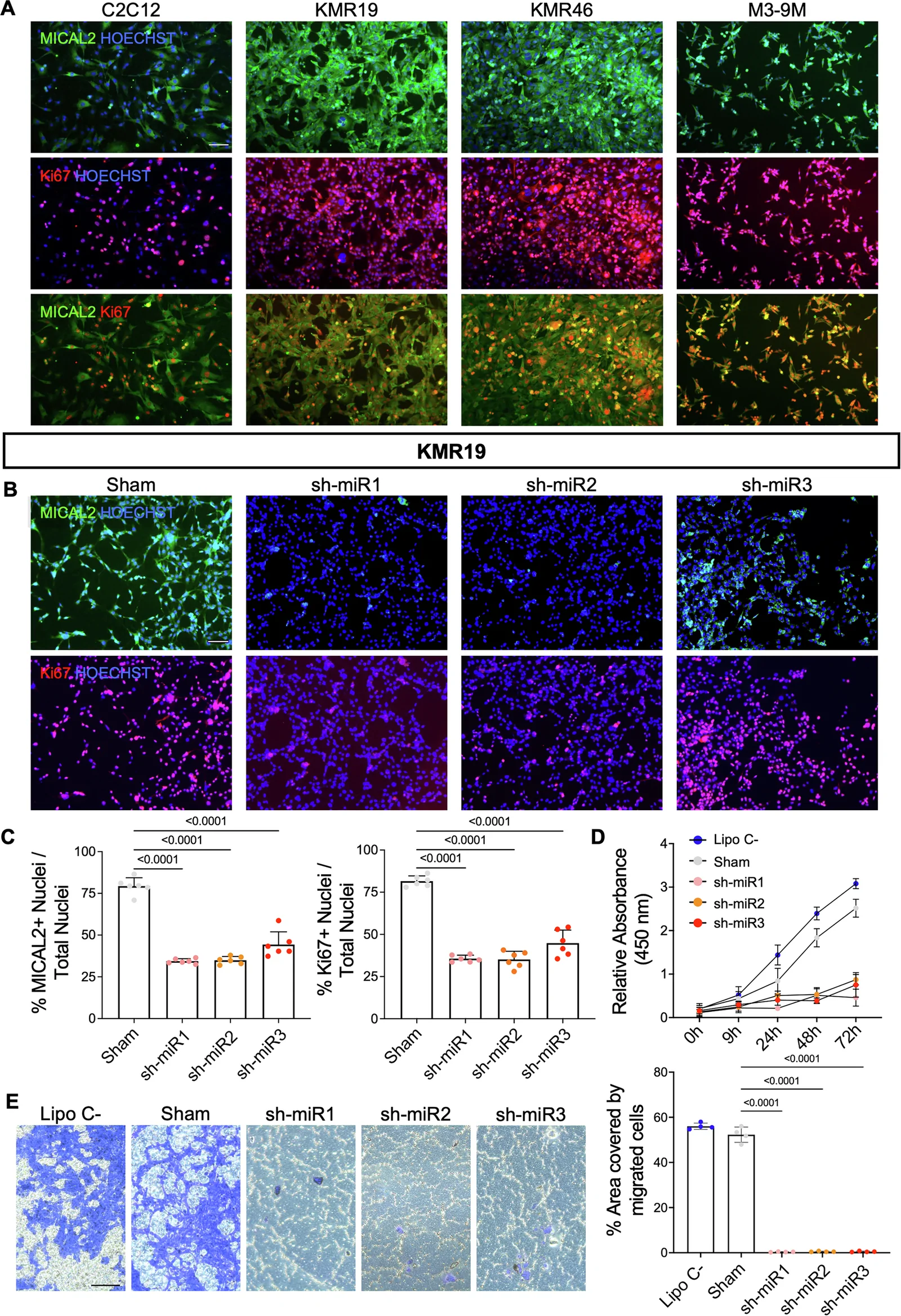
MICAL2 silencing reduces proliferative and migratory capacities in murine FN-RMS cells. Representative immunofluorescence images for MICAL2 (green) and Ki67 (red) in (**A**) proliferating C2C12 myoblasts and FN-RMS cancer cells and (**B**) KMR19 FN-RMS cells 72 h post-transfection with MICAL2-targeting plasmid constructs. Nuclei were stained with HOECHST (blue). Scale bar = 50 μm. **C** Relative quantification of MICAL2 (left) and Ki67 (right) shown as % expression (number of positive nuclei over total number of nuclei). Data are representative of six independent experiments (*n* = 6) and expressed as mean ± SD. Statistical analysis was performed using one-way ANOVA with Dunnett’s multiple comparisons test. **D** XTT assessment of metabolic activity in transfected KMR19 cells. Absorbance measurements were recorded at 0, 9, 24, 48, and 72 h post-transfection. Data are representative of three independent experiments (*n* = 3) and expressed as mean ± SD. Statistical analysis was performed using two-way ANOVA with Dunnett’s multiple comparisons test (Table S1). **E** Representative images of crystal violet staining of transfected KMR19 cells after 24 h migration in a Boyden chamber assay (left). Quantification is expressed as the % of the area covered by migrated KMR19 cells (right). Scale bar = 100 μm. Data are representative of four independent experiments (*n* = 4) and expressed as mean ± SD. Statistical analysis was performed using one-way ANOVA with Dunnett’s multiple comparisons test.

To elucidate MICAL2’s role in FN-RMS, we conducted a screening of three established murine FN-RMS cell lines, namely, KMR19, KMR46, and M3-9M [24, 25], to assess MICAL2 protein content. Each FN-RMS line was analyzed by immunofluorescence (IF), using C2C12 immortalized murine myoblasts as healthy controls. MICAL2 positivity was detected in all lines and was stronger in the FN-RMS cell lines compared to C2C12 (Fig. 1A, top panel). These images also revealed the co-localization of MICAL2 with Ki67, a proliferation marker (Fig. 1A, middle and bottom panels). The concurrent presence of both markers suggested a potential interplay between MICAL2 and the active proliferative phase of FN-RMS cells. Notably, these observations are consistent with reported overexpression of MICAL2 in other tumor types documented in literature [19, 21, 22, 26–29] and underscore the potential involvement of MICAL2 in FN-RMS tumor progression. As such, plasmid constructs encoding miR-based short hairpins (sh-miR1/2/3) targeting different regions of the *Mm Mical2* RNA were generated to knock down MICAL2 in murine FN-RMS cells. We first tested these constructs in KMR19 and KMR46 cells, which were isolated from primary KRAS mutated FN-RMS tumors, with the broader scope to generate MICAL2-silenced cells that allow for in vivo transplants in mice [24, 30]. To do this, cells were transfected with sh-miR1/2/3 for 9 h and allowed to recover for 72 h prior to subsequent analyses. In total, seven conditions were examined, and we confirmed that all three plasmid constructs significantly reduced MICAL2 protein abundance in both KMR19 and KMR46 cells by western blot (WB) analyses (Fig. S1A, B).

To assess proliferative capacity of the two FN-RMS cell lines post-MICAL2 knockdown (KD), IF staining for MICAL2 and Ki67 was performed. Decreased MICAL2 positivity was observed in treated KMR19 and KMR46 cells compared to sham cells, which were transfected with a plasmid carrying a miR targeting firefly luciferase (FLuc) as a non-targeting control (Figs. 1B and S2A, upper panels). Significant differences confirmed reduced MICAL2 expression, quantified as the proportion of MICAL2+ nuclei relative to the total number of nuclei present per field of view, upon exposure to MICAL2-targeting plasmids (Figs. 1C and S2B, left panel). Likewise, Ki67 exhibited similar patterns of reduced expression resulting from MICAL2 KD (Figs. 1B and S2A, lower panels), which was corroborated by quantifications demonstrating significantly fewer Ki67+ cells for each of the sh-miR-treated conditions compared to sham cells (Figs. 1C and S2B, right panel).

To further explore the impact of MICAL2 silencing on cell viability and metabolic activity, XTT assays were conducted. Absorbance readings obtained immediately before initiating the transfection (0 h) designated the baseline wherein all the tested conditions began with approximately the same density of viable cells (Figs. 1D and S2C). For KMR19, measurements recorded immediately post-transfection (9 h) showed that cell densities (expressed here as relative absorbance at 450 nm) remained relatively comparable across all conditions, but at the 24 h mark, significant differences emerged compared to the starting point (Fig. 1D; Table S1). Specifically, the absorbance measurements of the Lipofectamine control (Lipo C-) and sham cells were higher than those of the sh-miR conditions. This suggested that these cells without MICAL2 KD were more metabolically active, and these differences became even more evident at both 48 h and 72 h. The absorbance readings for sh-miR1-3 stayed quite consistent throughout the proliferation period, suggesting that the number of metabolically active cells remained relatively stable across the 72 h time span. Additionally, XTT assays performed on KMR46 suggested that these cells were more sensitive to the transfection procedure on top of MICAL2 KD (Fig. S2C), which was corroborated by the IF staining showing sparse populations of cells compared to KMR19 (Fig. S2A). Despite comparable baseline absorbances, a significant decline in viable cells was noticeable following plasmid uptake (9 h) that was further exacerbated by sh-miR mediated KD of MICAL2 as time progressed (24 h and 48 h) (Table S1).

Next, we assessed the impact of MICAL2 silencing on processes associated with cancer spread and metastasis. Transwell migration Boyden chamber assays were performed, wherein transfected KMR19 and KMR46 cells after the 72 h recovery period were seeded in OptiMEM and allowed to migrate toward serum-rich medium for 24 h. The results of this assay indicated that all MICAL2-silencing constructs impacted the migratory potential of the FN-RMS cells. Specifically, the percentage of surface area covered by migrated cells was significantly lower for sh-miR1-3, as evidenced by the sparse presence of cells observed on the membranes (Figs. 1E and S2D). In contrast, the Lipo C- and sham cells i.e. those retaining an overexpression of MICAL2, continued to display high migratory capacities and therefore, covered the majority of the surface area of the membrane. These results suggested that in addition to having an impact on the proliferative potential of FN-RMS cells, MICAL2 silencing also seemed to affect the migratory capacities of these cells, potentially by interfering with their propensity to undergo epithelial-mesenchymal transition (EMT).

### Silencing MICAL2 affects key signaling pathways associated with malignant progression in murine FN-RMS cell lines

**Fig. 2:**
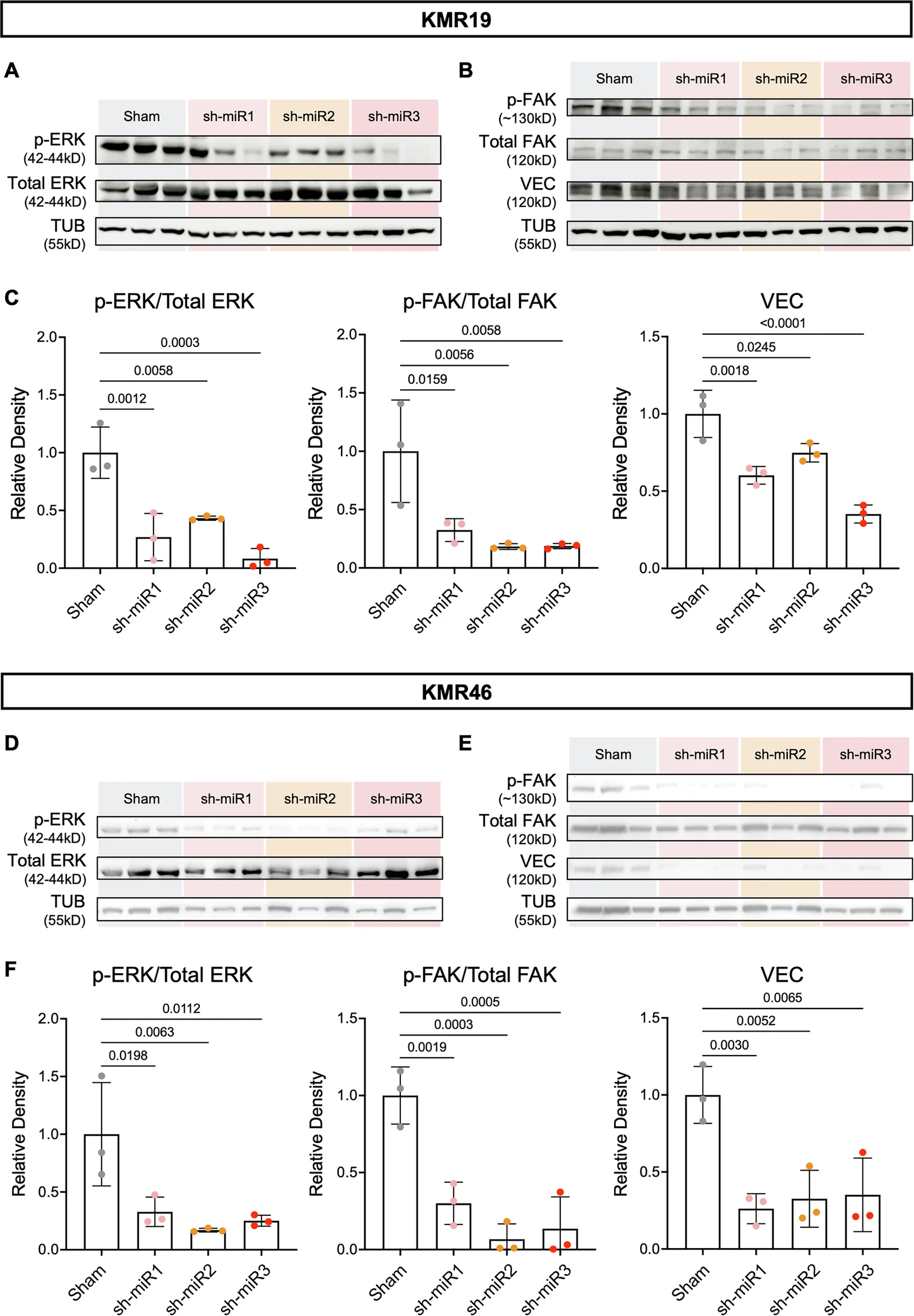
MICAL2 silencing in murine FN-RMS cells affects pathways associated with tumor aggressiveness. WB performed on murine FN-RMS cells collected 72 h after MICAL2 silencing via plasmid transfection. WB analyses of phosphorylated-ERK (p-ERK), total ERK, and tubulin (TUB) in (**A**) KMR19 and (**D**) KMR46 cells. WB analyses of phosphorylated-FAK (p-FAK), total FAK, vascular endothelial cadherin (VEC), and TUB in (**B**) KMR19 and (**E**) KMR46 cells. Quantification of WB analyses for (**C**) KMR19 and (**F**) KMR46 cells: proliferation markers were quantified as the ratio of p-ERK to total ERK, and migration markers were quantified as the ratio of p-FAK to total FAK and VEC normalized to TUB. Band intensities were normalized to sham and presented as relative density. Data are representative of three independent experiments (*n* = 3) and expressed as mean ± SD. Statistical analysis was performed using one-way ANOVA with Dunnett’s multiple comparisons test.

To investigate the mechanisms driving the observed reductions in FN-RMS cell proliferation and motility, we examined the effects of MICAL2 silencing on key signaling pathways known to facilitate tumor growth and invasion. A crucial signaling cascade involved in mediating normal cell proliferation and survival is the RAS-extracellular signal-regulated kinase (ERK) pathway. Therefore, to study the effects of MICAL2 silencing on this pathway, WB analyses were performed on KMR19 and KMR46 cells following MICAL2 KD, and the protein content of phosphorylated-ERK (p-ERK), the active form of the protein, showed reduced levels compared to sham, suggesting decreased activity of the RAS-ERK signaling pathway in both cell lines (Fig. 2A, D). Other proteins involved in pathways implicated in metastatic progression were also investigated, including focal adhesion kinase (FAK), whose activation upon phosphorylation sets off several signaling cascades promoting survival, migration, and invasion, and this hyperactivation is frequently observed in cancers [31]. Moreover, FAK is also implicated in EMT, a crucial process underlying tumor dissemination, as well as mediating the phosphorylation of vascular endothelial cadherin (VEC), which is involved in tumor vascularization [32]. As such, WB analyses of these migration and invasion-associated markers were performed, which demonstrated that MICAL2 KD with any of the three plasmids induced a decrease in phosphorylated-FAK (p-FAK) and VEC protein levels compared to sham (Fig. 2B, E). These combined results indicate that MICAL2 silencing triggers significant alterations (Fig. 2C, F) in important signaling cascades implicated in driving the aggressiveness of this cancer.

### Modulating MICAL2 expression in human FN-RMS cells recapitulates phenotypes observed in murine models

**Fig. 3:**
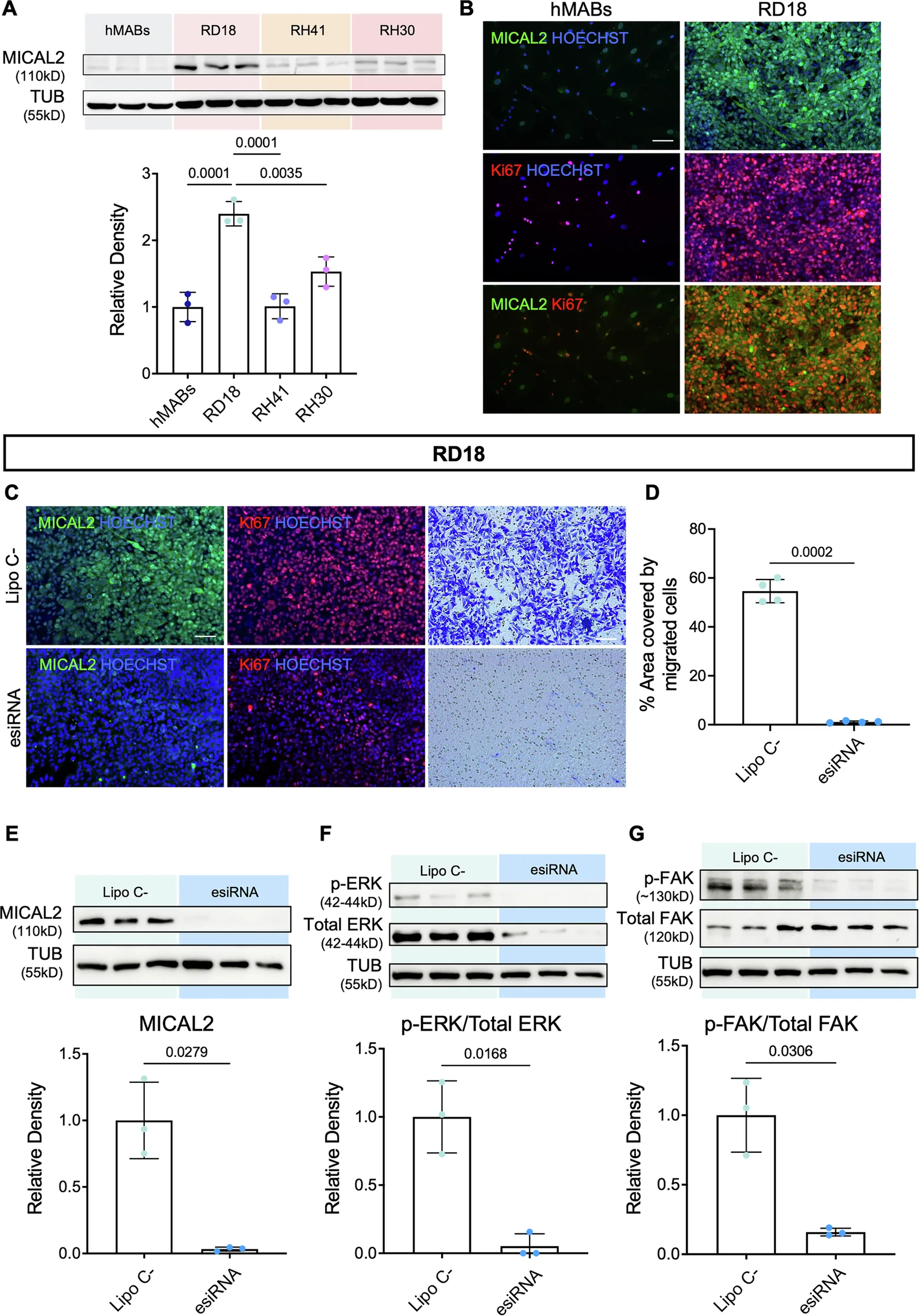
Silencing MICAL2 in human FN-RMS cells mirrors effects observed in murine cells. **A** WB analyses of MICAL2 and TUB protein levels in mesoangioblasts (hMABs), FN-RMS (RD18), and FP-RMS (RH41 and RH30) human cell lines (top panel). Band intensities were normalized to TUB, then to hMABs (healthy control), and expressed as relative density (bottom panel). Data are representative of three independent experiments (*n* = 3) and expressed as mean ± SD. Statistical analysis was performed using one-way ANOVA with Tukey’s multiple comparisons test. **B** Representative IF images for MICAL2 (green) and Ki67 (red) in hMABs and RD18 cells. Scale bar = 50 µm. **C** Representative IF images for MICAL2 and Ki67 in RD18 cells 72 h after transfection with esiRNA targeting MICAL2, and crystal violet staining of transfected RD18 cells after 24 h migration in a Boyden chamber assay. Scale bar = 50 µm. **D** Quantification of crystal violet staining is expressed as the % of the area covered by migrated RD18 cells. Data are representative of four independent experiments (*n* = 4) and expressed as mean ± SD. Statistical analysis was performed using unpaired *t*-test with Welch’s correction. WB analyses of (**E**) MICAL2, (**F**) p-ERK and total ERK, (**G**) and p-FAK and total FAK with TUB as loading controls in RD18 cells collected 72 h post-transfection (top panels). Quantifications of WB analyses (bottom panels): MICAL2 was normalized to TUB, proliferation markers were quantified as the ratio of p-ERK to total ERK, and migration markers were quantified as the ratio of p-FAK to total FAK. Band intensities were normalized to Lipofectamine control (Lipo C-) and presented as relative density. Data are representative of three independent experiments (*n* = 3) and expressed as mean ± SD. Statistical analysis was performed using unpaired *t*-test with Welch’s correction.

To investigate whether MICAL2 silencing holds potential translational relevance in humans, we assessed MICAL2 expression in three human RMS cell lines, specifically, RD18, RH41, and RH30, and compared them with healthy myogenic cells (human mesoangioblasts, hMABs) [33]. Interestingly, a significant elevation in MICAL2 protein abundance was only observed in RD18, a FN-RMS patient-derived cell line, while RH41 and RH30, which are of FP-RMS origin, did not exhibit a comparable increase (Fig. 3A). IF staining for MICAL2 and Ki67 displayed higher positivity for both markers in RD18 compared to hMABs and confirmed their colocalization (Fig. 3B), which was consistent with results obtained from their murine counterparts. RD18 cells were transfected with a commercially available engineered silencing RNA targeting MICAL2, and following which, showed reduced expression of both MICAL2 and Ki67 (Fig. 3C, left and middle panels). Furthermore, Boyden chamber assays demonstrated the significantly reduced migratory capacity of these transfected cells (Fig. 3C, right panels and 3D). WB analyses further confirmed the successful KD of MICAL2 protein (Fig. 3E), with significant downregulations of p-ERK and p-FAK (Fig. 3F, G), which corresponded with the signaling changes observed in MICAL2-silenced murine cells. Interestingly, in addition to decreased abundance of p-ERK, total ERK protein was also reduced in RD18 cells, perhaps due to altered transcriptional regulation or protein stability, while it remained unaffected in murine cell lines with MICAL2 silencing. These results support a role for MICAL2 in driving cancer-related phenotypes in a human FN-RMS model.

We further interrogated whether MICAL2 dysregulation is also observed in primary human RMS samples by analyzing a publicly available dataset (GSE108022) consisting of normal muscle (*n* = 5), FP-RMS (*n* = 35), and FN-RMS patient samples (*n* = 66), and of the 66 FN-RMS samples, 14 harbored mutations in either HRAS, NRAS, or KRAS (Fig. S3A). Compared with normal skeletal muscle, RMS tumors, encompassing both FP and FN subtypes, exhibited considerable heterogeneity in *MICAL2* expression. Interestingly, by subsetting for RAS mutations in FN-RMS, these samples showed a bias toward higher *MICAL2* expression (Fig. S3B), suggesting a potential correlation between RAS pathway activation and MICAL2 dysregulation within FN-RMS.

### MICAL2 silencing attenuates tumor growth and metastasis in vivo

Results obtained from the previously described experiments provided proof of concept that short hairpin-mediated MICAL2 silencing reduces tumor cell proliferative and migratory behavior, thereby motivating further investigation into whether these in vitro effects would translate in vivo. Using the KMR19 cell line, we proceeded with sh-miR1 and sh-miR2 only, which target the non-coding and the coding region of *Mical2* mRNA, respectively. To allow non-invasive monitoring of tumor progression in vivo, KMR19 cells underwent two transductions (see Methods), and the resulting cells selected from these two transductions were positive for firefly luciferase (FLuc + ) and contained a Tet inducible sh-miR1/2 sequence targeting *Mm Mical2* mRNA (or eGFP in the case of sham), allowing inducible MICAL2 knockdown upon doxycycline administration. XTT assays were performed on these transduced cell lines to assess the effect of doxycycline exposure. Indeed, doxycycline administration reduced metabolic activity in the MICAL2-targeting cell lines but not in the sham control, and notably, the results showed this effect was reversible upon doxycycline withdrawal (Fig. S4; Table S2).

**Fig. 4:**
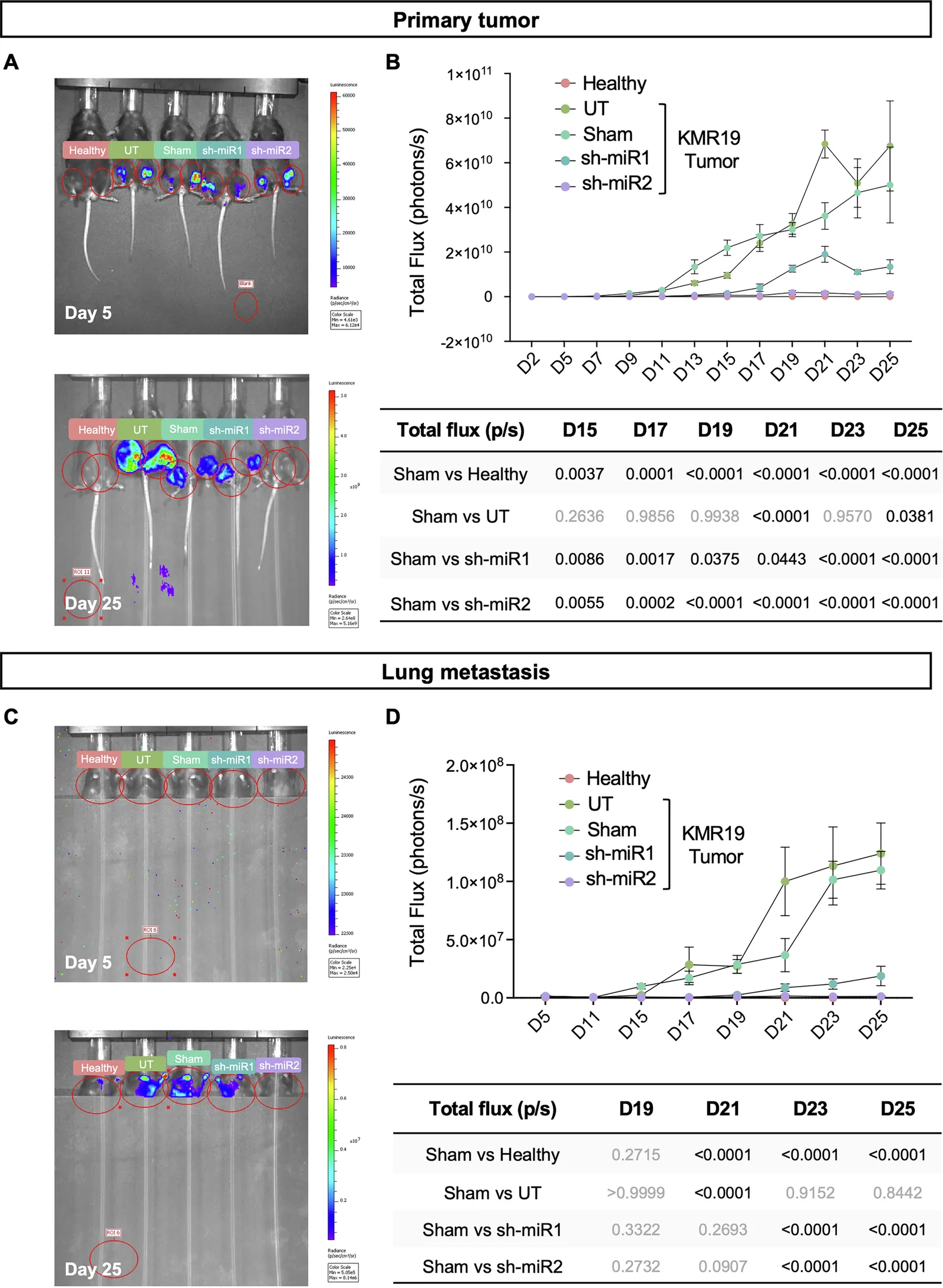
MICAL2 silencing impedes primary tumor growth and metastasis progression in FN-RMS tumor-bearing mice. Representative bioluminescent images of (**A**) primary tumors and (**C**) lung metastasis in healthy, UT, sham, sh-miR1, and sh-miR2 tumor-bearing mice. Images were taken every other day from day 5 to day 25 post-injection. Quantification of the BLI signal for (**B**) primary tumors and (**D**) lung metastasis. Data are expressed as mean ± SD (*n* = 6 mice per group). Statistical analysis was performed using two-way ANOVA with Tukey’s multiple comparisons test (Table S3).

Following this validation of the inducible system, five experimental groups of mice were subjected to bilateral intramuscular injections in their *tibialis anterior* (TA), *gastrocnemius* (GN), and *quadricep* (Q) muscles with saline (negative controls, Healthy), KMR19 FLuc+ cells (positive controls, untreated or UT), or KMR19 FLuc+ cells with sh-miRs (sham, sh-miR1, and sh-miR2). UT and sham tumor-bearing mice are collectively referred to as “non-treated” mice, and the complete experimental timeline is illustrated in Fig. S5A. Doxycycline administration began on the same day that tumor cell injections took place and continued throughout the duration of the experiments to maintain MICAL2 KD. Tumor progression in the hindlimbs was monitored by bioluminescence imaging (BLI, FLuc photon flux). Imaging was regularly performed on alternate days after tumor engraftment and continued until the end of the experiment (Figs. 4A and S5B). Imaging on day 5 confirmed establishment of the tumor mass (Fig. 4A, top panel), and it was not until day 15 that significant differences between experimental conditions arose (Figs. 4B and S5B; Table S3). Specifically, on day 15, the luminescent signal obtained from “non-treated” tumor-bearing mice was significantly more intense than all the other groups, and this trend continued throughout the whole experiment until day 25 (Fig. 4A, bottom panel and 4B), signifying that MICAL2-silencing reduced primary tumor burden. Notably, not only did time and treatment both have significant main effects (*p* < 0.0001), a significant interaction between these factors (*p* < 0.0001) was also observed, indicating that tumor growth dynamics differed between the conditions over time. Additionally, knowing that FN-RMS can develop metastasis predominantly in the lungs, metastasis formation was also followed by BLI, and the first signs of positivity emerged on day 15 in “non-treated” tumor-bearing mice with significant differences becoming evident on day 21 until the end of the experiment (Figs. 4C, D and S5C; Table S3). Interestingly, the sh-miR1 treated group started showing some traces of metastasis from day 21 that remained much lower than their “non-treated” tumor-bearing counterparts, and the sh-miR2-treated group displayed minimal signs of metastasis throughout the entire observation period. As with the primary tumor, a significant interaction between time and treatment was observed (*p* < 0.0001), and each factor had significant effects (*p* < 0.0001) on metastasis development. These findings were further corroborated by ex vivo BLI of lung explants, indicating widespread metastasis in “non-treated” tumor-bearing mice, limited traces in sh-miR1-treated mice, and photon flux at or near background levels in sh-miR2-treated mice, suggesting a strong suppression of metastatic spread (Fig. S6).

### Functional performance improves in FN-RMS tumor-bearing mice upon MICAL2 silencing

**Fig. 5:**
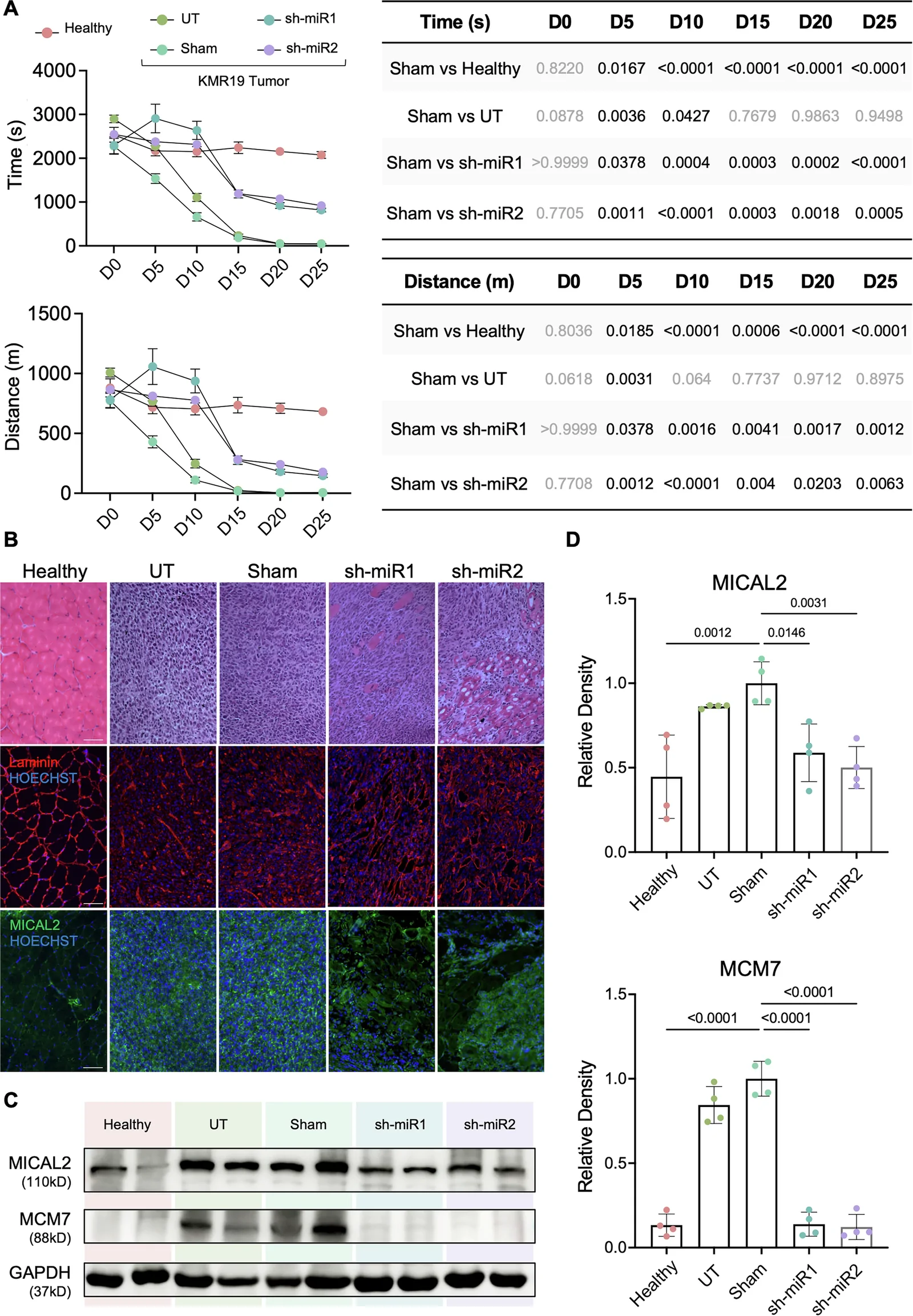
Improved functional performance and preserved myofibers are observed upon MICAL2 silencing in FN-RMS tumor-bearing mice. **A** Functional assessment by treadmill exhaustion tests performed 0-, 5-, 10-, 15-, 20- and 25-days post-tumor injection. From top to bottom, graphs of Time (s) and Distance (m) of run. Data are expressed as mean ± SD (*n* = 6 mice per group). Statistical analysis was performed using two-way ANOVA with Tukey’s multiple comparisons test (Table S4). **B** Representative images of hematoxylin and eosin staining (top panel) and IF images for laminin (red, middle panel) and MICAL2 (green, bottom panel). Nuclei were stained with HOECHST (blue) on in vivo muscle tissue/tumor sections. Scale bars = 100 µm. **C** WB analysis of MICAL2, MCM7, and GAPDH in muscle/tumor tissue samples from healthy and FN-RMS tumor-bearing mice. **D** Quantification of WB analyses where data are representative of samples from four mice per group and are expressed as mean ± SD. Band intensities were normalized to GAPDH, then to sham, and presented as relative density. Statistical analysis was performed using one-way ANOVA with Tukey’s multiple comparisons test.

Since tumor growth can impair ambulation capacities, further in vivo examination was to challenge the mice with treadmill exhaustion tests. Firstly, a baseline readout of the physical capacity of all the healthy mice prior to tumor injections (day 0) was obtained; then, treadmill exhaustion tests were conducted every five days to monitor the functional performance of the mice over the course of the experiment. The results of these tests were expressed as four different parameters, namely time (s), distance (m), work (J), and power (W). Analysis of time and distance revealed that the “non-treated” tumor-bearing mice displayed progressively reduced running time and distance (Fig. 5A). In contrast, the healthy and sh-miR-treated MICAL2 KD mice maintained relatively stable running times and distances until day 10, and there were no significant differences in either parameter between healthy mice and the two groups of sh-miR-treated mice until this time point, suggesting comparable performance levels (Fig. 5A; Table S4). Based on these two parameters, work and power were calculated as previously described [23, 30], and the results were in accordance with those obtained for time and distance (Fig. S7A; Table S4). Moreover, histological analyses suggested that FN-RMS tumors largely replaced muscle tissue in the hindlimbs of “non-treated” tumor-bearing mice, while both sh-miR1 and sh-miR2 treatment could contain tumor progression and better maintain muscle structure in place (Fig. 5B). More specifically, hematoxylin and eosin staining performed on the in vivo samples demonstrated that while the “non-treated” mice only showed tumor mass, the sh-miR-treated mice were able to retain some myofibers (Fig. 5B, upper panels), which correlated with their improved functional performances. Myofibers were again detected in the sh-miR-treated mice by staining for laminin, whereas the “non-treated” mice showed irregular and unorganized laminin expression in addition to prolific MICAL2 expression (Fig. 5B, middle and lower panels). On the other hand, sh-miR-treated mice displayed a mixture of tumor and preserved myofibers and accordingly exhibited high MICAL2 expression in the tumor areas but much less in the myofibers (Fig. 5B, lower panels). WB analysis of muscle and tumor tissue proteins corroborated that MICAL2 was indeed significantly reduced in sh-miR-treated mice compared to “non-treated” controls (Fig. 5C and D, upper panel). Moreover, given that MICAL2 silencing had demonstrated modulating effects on cell proliferation, minichromosome maintenance complex component 7 (MCM7) protein abundance was also evaluated (Fig. 5C and D, lower panel). MCM7 plays a crucial role in DNA replication and represents not only a marker for cell proliferation but also a driver of tumorigenesis upon dysregulated expression [34–36]. In line with previous studies describing elevated MCM7 expression in different malignancies [37], MCM7 was upregulated in the “non-treated” mice but reduced in sh-miR-treated mice, consistent with the observed attenuation in tumor growth. Additionally, after assessing the weight of tumor, muscle, metastatic tissue, and filter organs, it was noticeable that tumor weight comprised ~30% of the total body weight of “non-treated” tumor-bearing mice, while tumor weight was significantly lower, accounting for only ~12% of the total body weight, in sh-miR-treated mice (Fig. S7B). Overall, the combined results indicated that MICAL2 depletion upon treatment with sh-miR1/2 significantly improved functional performance by hampering tumor growth while preserving intact muscle fibers.

### Bulk RNA-seq reveals suppressed tumorigenic programs and enhanced myogenic integrity with MICAL2 silencing

**Fig. 6:**
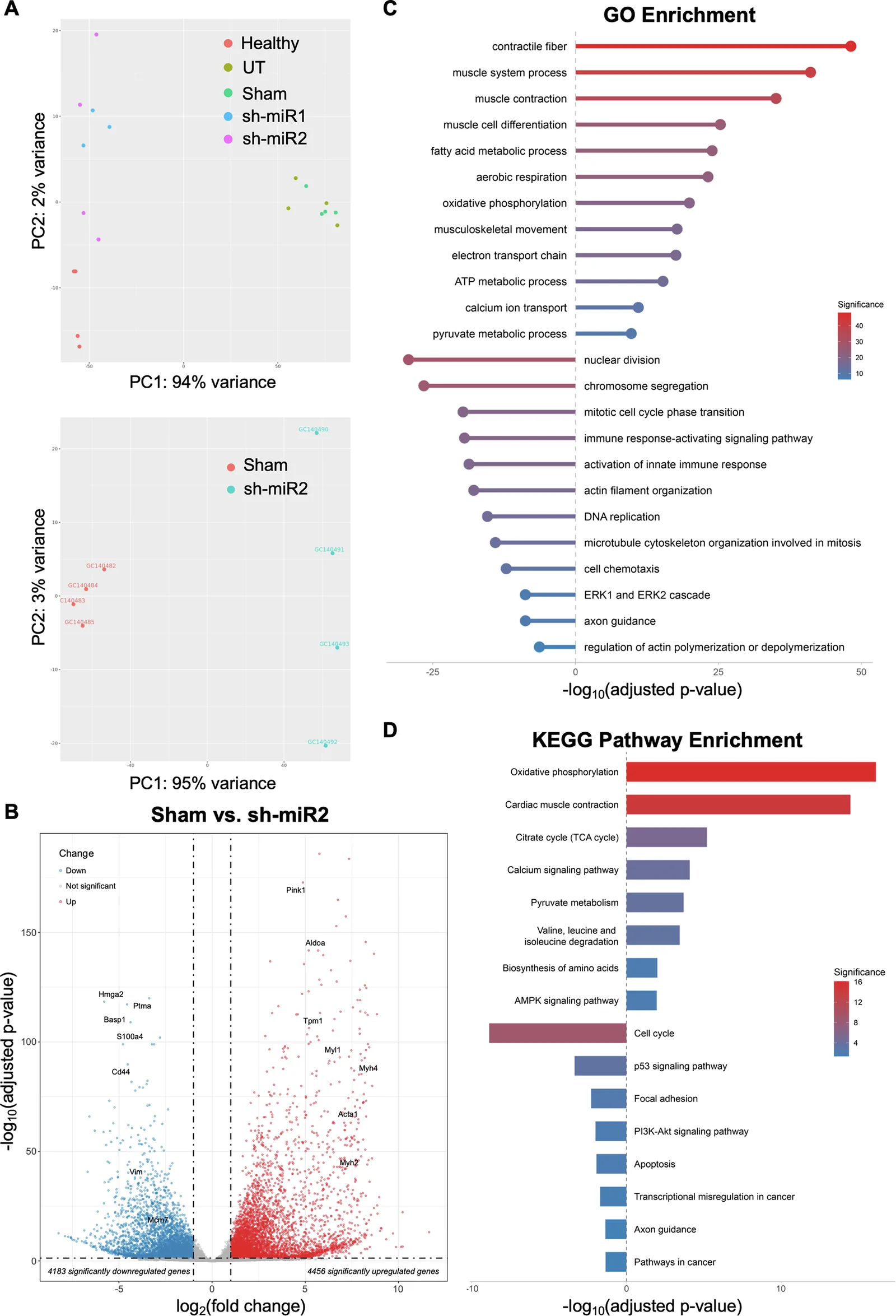
RNA-seq analysis reveals that MICAL2 silencing in FN-RMS tumor-bearing mice appears to suppress tumor programs and promote normal skeletal muscle phenotype. Bulk RNA-seq was performed on in vivo muscle/tumor tissues with samples from four mice per group submitted for analysis. **A** PCA plots displaying all experimental groups (top) and sham vs. sh-miR2 (bottom). **B** Volcano plot of the DEGs between sham and sh-miR2. Results of (**C**) GO and (**D**) KEGG pathway enrichment analyses based on the differentially up- and downregulated genes between sham and sh-miR2. Bars indicate -log_10_ (adjusted *p*-value) for selected terms and pathways: positive values indicate upregulated while negative values indicate downregulated terms and pathways. Color gradient represents the magnitude of statistical significance.

To assess the molecular impact of MICAL2 silencing on tumor progression and muscle integrity, bulk RNA-sequencing was performed to compare tumor and muscle tissue from FN-RMS tumor-bearing mice to muscle tissue from healthy controls (Figs. 6, S8, and S9). Principal component analysis (PCA) from the RNA-seq of all samples revealed a 94% PC1 variance among healthy muscle tissue versus “non-treated” tumors, whereas a clear shift toward healthy tissue was observed for sh-miR-treated mice, clustering all together with a 2% PC2 variance (Fig. 6A, top panel). When comparing only sh-miR2-treated and sham mice, the stark differences were again emphasized with PC1 explaining the vast majority of variance (95%) and PC2 contributing minimally (3%), demonstrating once more that most of the variability lies between the two groups (sh-miR2 vs. sham) rather than within each group (Fig. 6A, bottom panel). For downstream comparisons, UT data is not explicitly shown due to their high similarity to sham, as evidenced by their close clustering in Fig. 6A.

Volcano plots of differentially down- and up-regulated genes underscored significant differences between “non-treated” tumor-bearing mice and both healthy and sh-miR-treated mice (Figs. 6B and S8A, B). Among the most differentially expressed genes, proliferation and migration markers such as *S100* family genes, *Cd44*, *Vim*, *Ptma*, and *Hmga2* were highly expressed in “non-treated” tumor-bearing tissue compared to healthy muscle (Fig. S8A), and, interestingly, all of these genes were downregulated when comparing “non-treated” vs. sh-miR-treated tissues (Figs. 6B and S8B). Moreover, key myogenic marker genes belonging to the myosin and actin families, such as *Myl1, Myh2, Myh4, Tpm1*, and *Acta1*, were downregulated in “non-treated” tumor-bearing murine tissue, compared to healthy tissue, and intriguingly, all of these genes were upregulated when comparing sh-miR-treated to “non-treated” tissues.

In addition, both sh-miR1- and sh-miR2-treated tissues showed an upregulation of *Aldoa* and *Pink1*, which are genes critical for muscle energy homeostasis, compared to “non-treated” tissues (Figs. 6B and S8B). Although *Aldoa* is a key enzyme of glycolysis, in muscle, it also contributes to other functions such as fiber maintenance, regulation of cell shape and motility, striated muscle contraction, and actin cytoskeleton organization [38, 39]. Consistent with these roles, *Aldoa* expression was reduced in “non-treated” tumors relative to healthy muscle, but MICAL2 silencing appeared to restore it toward physiological levels, likely reflecting preserved myofibers and functional metabolism rather than enhanced tumor glycolysis. Similarly, the upregulation of *Pink1*, a putative mitochondrial serine/threonine kinase, in sh-miR-treated tissues hinted at improved mitochondrial quality and oxidative metabolism taking place in residual myofibers [40, 41]. Therefore, treatment with sh-miR1/2 seemed to induce increased expressions of *Aldoa* and *Pink1* that could be supporting metabolic adaptations involved in maintaining muscle integrity.

On the pathway level, gene ontology (GO) analyses of these differentially expressed genes (DEGs) revealed that, as expected, comparing “non-treated” to healthy tissues demonstrated the clear disruption of muscle-specific gene programs and the significant enrichment of cell-cycle and mitotic pathways (Fig. S8C). However, upon MICAL2 depletion, this pattern was reversed, as muscle-related biological processes and cellular components, such as muscle system process, muscle cell differentiation, and contractile fiber, were highly enriched in sh-miR-treated mice, suggesting a reinstatement of contractile machinery and myofiber architecture that were normally destroyed in tumor-dominated muscle tissue (Figs. 6C and S8D). Additionally, the upregulation of aerobic respiration, oxidative phosphorylation, electron transport chain, and ATP metabolic process indicated enhanced mitochondrial energy production, in line with improved endurance exhibited during the treadmill exhaustion tests. In contrast, biological processes significantly downregulated in sh-miR-treated mice were primarily concerning cell cycle progression, immune response, and cytoskeletal remodeling, pointing to the suppression of tumor cell proliferation and invasion.

GO findings were reinforced by KEGG enrichment analysis, which associated increased oxidative metabolic capacity (oxidative phosphorylation, citrate cycle, amino acid degradation, AMPK signaling pathway) and attenuation of oncogenic signaling (cell cycle, focal adhesion, PI3K-AKT, transcriptional misregulation in cancer) with MICAL2 silencing treatment (Figs. 6D and S8E, F). Intriguingly, only modest differences were found when comparing healthy muscles to sh-miR-treated tissues, where there were just hundreds rather than thousands of DEGs (Fig. S9A, B). Correspondingly, the enriched terms resulting from GO and KEGG analyses were largely unrelated to muscle biology and were primarily associated with sensory receptor pathways and some expected immune-related differences (Fig. S9C, D, E, F), further substantiating that MICAL2 KD appeared to cause a shift toward a transcriptional state resembling healthy muscle.

### Proteomic analysis corroborates repression of oncogenic signaling and reinforcement of muscle identity with MICAL2 silencing

**Fig. 7:**
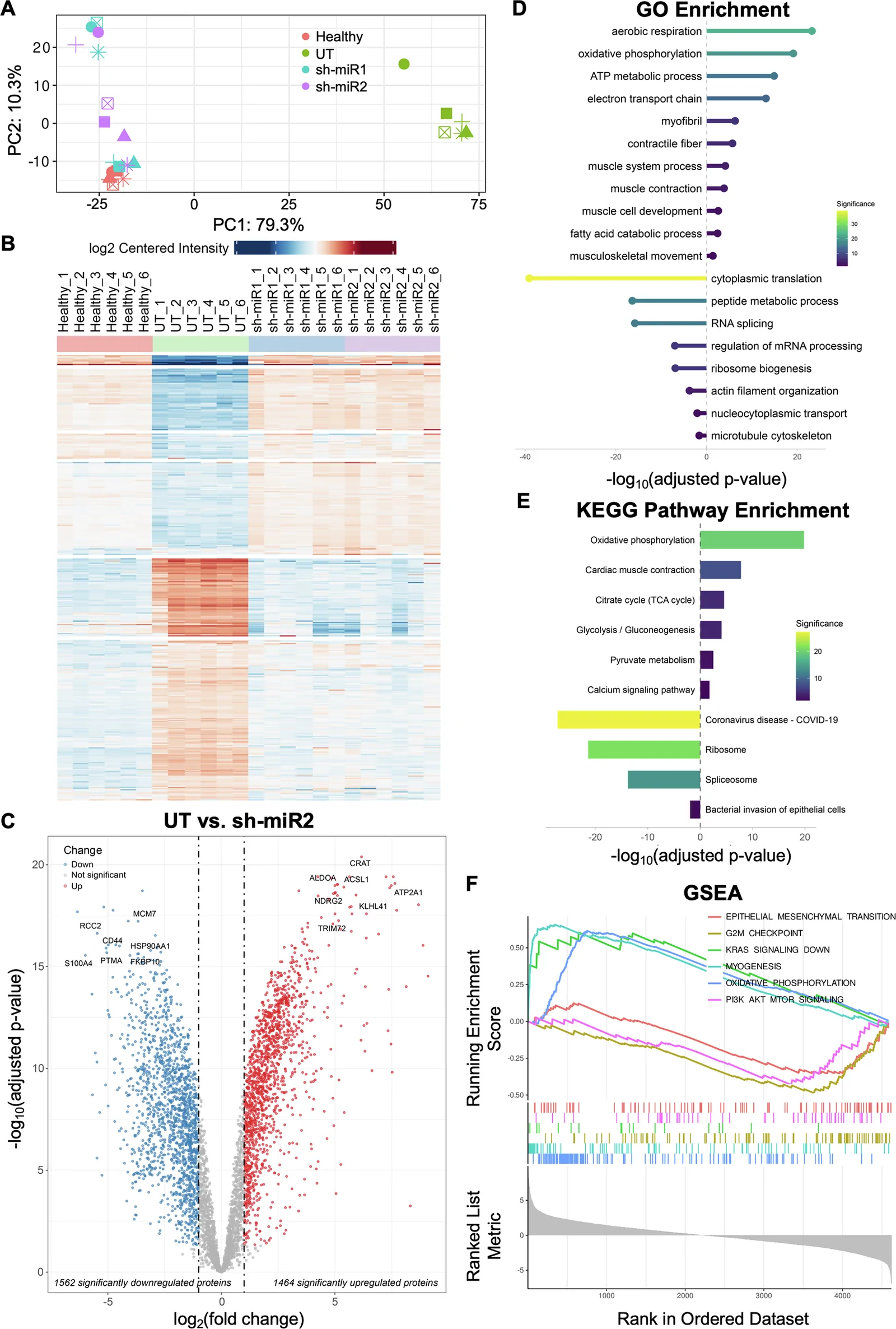
Mass spectrometry analysis corroborates RNA-seq results demonstrating positive effects of MICAL2 silencing in FN-RMS tumor-bearing mice. Mass spectrometry was performed on in vivo muscle/tumor tissues with six mice analyzed per group. For the UT condition, samples from three UT mice and three sham mice were pooled together. **A** PCA plot displaying all experimental groups. **B** Heatmap representing an overview of expression of all significant (differentially abundant) proteins (rows) in all samples (columns). **C** Volcano plot of DAPs between UT and sh-miR2. Results of (**D**) GO, (**E)** KEGG, and (**F**) GSEA based on up- and downregulated proteins between UT and sh-miR2. Bars indicate -log_10_ (adjusted *p*-value) for selected terms and pathways: positive values indicate upregulated while negative values indicate downregulated terms and pathways. Color gradient represents the magnitude of statistical significance. For GSEA, positive NES indicates enrichment among proteins upregulated in sh-miR2 relative to UT. Full GSEA statistics are provided in Table S5.

In addition to bulk RNA-seq, we also performed mass spectrometry-based proteomics analysis on the healthy and tumor-bearing mouse muscle tissues to determine whether the observed transcriptional changes of MICAL2 silencing were reflected at the protein level and to obtain further insights into its functional impact in vivo. For this analysis, “non-treated” samples comprising three UT and three sham were pooled together and analyzed as one condition, collectively designated as UT in the figures. Remarkably, the results substantiated the RNA-based findings on multiple levels. Firstly, the PCA plot of all the experimental groups combined revealed a 79.3% PC1 variance separating the “non-treated” tumor tissues from the healthy/sh-miR-treated tissues (Fig. 7A). Comparable to before, these latter groups clustered closely with only a 10.7% PC2 variance, which recapitulated that most of the observed differences were between groups (healthy + sh-miR-treated vs. “non-treated”) rather than within groups (healthy vs. sh-miR-treated). The clear similarities between the healthy and sh-miR-treated samples were further illustrated by the heatmap of differentially abundant proteins (DAPs) showing that these conditions exhibited highly similar abundance patterns while in contrast, the “non-treated” group formed a distinct cluster with its own proteomic signature (Fig. 7B).

Volcano plots showcasing the DAPs between the different experimental groups again demonstrated the marked differences between the “non-treated” conditions and the healthy and sh-miR-treated conditions (Figs. 7C, S10A, B, and S11A, B). Notably, among the proteins downregulated with sh-miR treatment was MCM7 (Figs. 7C and S10B), which mirrored the previous WB results (Fig. 5C) and reinforced the connection between MICAL2 silencing and reduced proliferative activity. Furthermore, S100A4, CD44, and PTMA protein abundances were also reduced with MICAL2 silencing, consistent with their transcriptional downregulation observed in the RNA-seq data. Markers associated with cell proliferation, migration, and EMT including MAPK1/3, PTK2, and MMP2 showed significant reductions in protein abundance following sh-miR treatments (Fig. S12A, B). Conversely, myosin heavy chain isoforms including MYH4, MYH7, and MYH8 were highly suppressed in “non-treated” tumor-bearing mice but significantly upregulated upon MICAL2 silencing, reiterating the enhanced preservation of normal skeletal muscle phenotype (Fig. S12C).

As expected, GO analysis based on these DAPs produced similar results to that of the RNA-seq. Proteins downregulated in the “non-treated” tumor setting (relative to healthy) were once more associated with muscle-related biological processes while the upregulated proteins were not only related to cancer spread and survival, but also linked to cytoskeletal remodeling and nucleocytoplasmic transport, reminiscent of MICAL2’s function (Fig. S10C). However, GO terms enriched among proteins upregulated upon MICAL2 silencing, such as oxidative phosphorylation, ATP metabolic process, contractile fiber, and muscle system process, suggested a shift away from tumor-driving processes and toward a more muscle-like state, involving increased mitochondrial metabolism and improved muscle structural integrity (Figs. 7D and S10D). Concurrently, a downregulation of cellular machinery sustaining tumor growth and invasion, including cytoplasmic translation, RNA splicing, ribosome biogenesis, and actin filament organization, reflected diminished oncogenic signaling.

KEGG analyses similarly revealed that MICAL2 silencing seemed to suppress biosynthetic and inflammatory pathways, which were elevated in “non-treated” tumors (Fig. S10E). In addition, pathways supporting a physiological muscle metabolic state were also identified in sh-miR-treated mice, with enrichment of oxidative phosphorylation, citrate cycle, and calcium signaling (Figs. 7E and S10F). Nevertheless, as these mice still bore tumors despite preserving some myofibers, GO analyses comparing them to healthy controls revealed the expected enrichment of terms associated with inflammatory responses and a reduction of muscle contractile and structural components (Fig. S11C, D), while minimal differences were detected in KEGG pathways (Fig. S11E, F).

Gene set enrichment analysis (GSEA) was also performed to complement these findings (Figs. 7F and S10G, H; Table S5), revealing that while myogenesis and oxidative phosphorylation were strongly depleted in “non-treated” tumors relative to healthy muscle (NES = –3.27 and –3.39; p.adj < 0.001), these hallmark gene sets became the most significantly enriched with sh-miR treatment (NES = +2.99 and +3.03; p.adj < 0.001). Interestingly, KRAS Signaling - Down displayed the same shift, indicating that silencing MICAL2 seemed to attenuate KRAS pathway activity. Conversely, EMT, G2M checkpoint, and PI3K-AKT-mTOR signaling showed the reverse pattern of being highly enriched in “non-treated” tumors and suppressed with MICAL2 KD. However, compared to healthy muscle, sh-miR-treated mice still exhibited downregulation of myogenesis and oxidative phosphorylation (Fig. S11G, H; Table S5), signaling that MICAL2 silencing does not completely restore the normal muscle proteome.

To further validate the proteomics results, we confirmed several markers exhibited consistent alterations at both RNA and protein levels. Given MICAL2’s role in cytoskeletal dynamics, we focused on actin network regulators whose activities are dependent on MICAL2-mediated actin oxidation. As such, we found that cofilin-1 (CFL1), profilin-1 (PFN1), and actin-related protein2/3 complex subunit 3 (ARPC3) gene expression and protein abundance were reduced in sh-miR-treated mice to levels more comparable to healthy controls (Fig. S13), suggesting that MICAL2 silencing may have diminished cytoskeletal remodeling by decreasing CFL1-driven severing of actin filaments [42], lowering the need for PFN1-mediated actin monomer turnover [43], and limiting ARPC2/3-dependent nucleation of branched actin networks [44]. Moreover, as mentioned previously, markers of tumor growth and invasion (MCM7, CD44, and PTMA) also showed concordant transcriptional and proteomic downregulation with sh-miR-treatment, once more substantiating the attenuation of tumor activity (Fig. S14).

## Discussion

Rhabdomyosarcoma is a malignant neoplasm characterized by dysregulated myogenesis that exhibits survival rates closely linked to histological subtypes [45]. Despite most RMS cases being classified as fusion negative, comprehensive studies defining the drivers of FN-RMS development and progression remain limited, and the molecular basis of its myogenic dysregulation and aberrant cytoskeletal dynamics is poorly understood. In this context, MICAL2 overexpression has been reported across multiple malignancies, where it promotes proliferative, migratory, and invasive behavior through actin cytoskeleton remodeling [26, 46]. Here, we extend this oncogenic role to FN-RMS and identify MICAL2 as a mediator of FN-RMS tumor aggressiveness, demonstrating that its silencing attenuates malignant phenotypes both in vitro and in vivo by altering key molecular pathways implicated in facilitating tumor growth and dissemination.

Among the pathways altered by MICAL2 silencing was RAS-ERK signaling, which, as previously mentioned, is frequently constitutively active in FN-RMS and leads to uncontrolled cell proliferation and resistance to apoptosis-inducing drugs [14, 47]. Notably, MICAL2 KD induced a significant reduction in ERK signaling, echoing effects of pharmacological inhibition of this hyperactive pathway, which has been previously shown to hamper tumor progression in colorectal, lung, and breast cancers [48, 49]. Additionally, because metastatic disease in FN-RMS is strongly associated with poor prognoses, the observed downregulation of activated FAK signaling is particularly relevant. Existing literature suggests that reduced FAK activity can mitigate malignant phenotypes by limiting cell proliferation and invasion and inhibiting tumor angiogenesis, which are mechanisms that may also be relevant contributors to FN-RMS progression [50, 51]. Complementing these changes, the decrease in VEC expression is also notable as it is known to facilitate cancer progression by promoting tumor angiogenesis and vasculogenic mimicry [52, 53]. Moreover, FAK-mediated VEC phosphorylation is instrumental in maintaining cells in a mesenchymal state [32, 54], which corresponds to loss of barrier function and acquisition of invasive phenotype, and its downregulation therefore suggests that MICAL2 silencing triggers changes that hint at a partial reversal of EMT-associated processes.

Multi-omics analyses further illustrated the multifaceted role of MICAL2 in shaping both tumor behavior and muscle integrity by revealing that its silencing induces a transcriptional and proteomic shift that simultaneously suppresses oncogenic programs and reinforces myogenic structure and function. This convergence nicely highlights the duality of MICAL2’s role as a cytoskeletal regulator; while physiological MICAL2 expression is essential for myogenic lineage commitment [23], its overexpression appears to redirect actin turnover toward promoting cancerous behavior [55]. Although we did not directly evaluate changes in cytoskeletal remodeling, our transcriptomic and proteomic results strongly suggest that actin dynamics were altered following MICAL2 KD. In particular, GO analyses highlighted multiple variations of “actin filament organization,” “microtubule cytoskeleton,” and “regulation of actin depolymerization” among the significantly enriched terms that were downregulated in MICAL2-silenced tumors. Furthermore, CFL1, PFN1, and ARPC3 were all significantly reduced to levels more comparable to healthy muscle, suggesting a broad attenuation of actin remodeling machinery, and direct cytoskeletal imaging represents an important next step for obtaining more definitive evidence.

Consistent with reduced actin turnover capacity, KEGG analysis of bulk RNA-seq data identified concurrent downregulation of cell cycle, focal adhesion, transcriptional misregulation in cancer, and PI3K-AKT pathways. GSEA of hallmark gene sets further confirmed these findings at the protein level with additional suppression of EMT and G2M checkpoint programs, which collectively indicate a repression of proliferative and invasive capacities with MICAL2 KD. Notably, on the transcriptional level, p53 signaling and apoptosis also emerged among these pathways, and at first glance, this may seem paradoxical given their associations with tumor suppression [56]. However, within the context of reduced tumor growth, diminished metastatic potential, and better functional outcomes observed in the treated mice, this pattern most likely reflects a reduction of upstream cellular stresses, as opposed to a dampening of tumor-suppressive mechanisms. This is in line with MICAL2’s known involvement in contributing to cellular stresses through F-actin oxidation and ROS production [26, 55]. With MICAL2 silencing, it is plausible that these stresses are attenuated, resulting in suppression of p53-driven stress responses. Nevertheless, since apoptosis-related assays were not performed, these results should be regarded as just transcriptional alterations in stress-associated signaling rather than definitive evidence of altered apoptotic activity.

Furthermore, the transcriptomic and proteomic data revealed that concomitant with diminution of malignant features was an upregulation of sarcomeric structural proteins and oxidative metabolic enzymes, which support a physiological muscle phenotype. On account of the fact that these analyses were performed on bulk tumor tissue, this signature likely reflects contributions from both tumor cell reprogramming and myofiber preservation, and single-cell approaches will be required to distinguish the two. Nonetheless, the functional relevance of these changes was evident in vivo, where MICAL2 silencing was linked to preserved myofibers and improved physical performance, with corresponding enhanced mitochondrial metabolism observed at both RNA and protein levels. This aligns with our prior work where we achieved similar phenotypic outcomes of reduced tumor growth and diminished metastatic potential by inhibiting the mitochondrial pyruvate carrier via reintroduction of miR-449a and miR-340 [57]. These findings converge on the same principle of reprogramming FN-RMS cell identity and metabolism to push the cells toward a non-tumorigenic state, with MICAL2 silencing specifically promoting a more oxidative, muscle-like signature while miRNA treatment causes a shift toward a quiescent, mesenchymal state.

To contextualize these findings within human disease, we examined three human RMS cell lines and found that MICAL2 expression was increased in RD18, an FN-RMS-derived line with an NRAS mutation, and not in the others of FP-RMS origin. In accordance with the murine results, MICAL2 silencing in RD18 reduced migratory behavior, complemented by altered ERK and FAK activity. Notably, these results align with a recent study that showed that MICAL2 promotes KRAS signaling and ERK1/2 pathway activation in pancreatic ductal adenocarcinoma [58]. In that investigation, Garg and colleagues showed that silencing MICAL2 resulted in attenuation of ERK pathway activity and suppression of both EMT and KRAS Signaling hallmark gene sets, findings that we recapitulated at the proteomic level. Therefore, the reduction of p-ERK in MICAL2-silenced RD18 cells falls in line with the notion that MICAL2 may modulate RAS-associated signaling; however, total ERK was also reduced in these cells but not in the pancreatic cancer models, and this may reflect differences in transcriptional regulation of the ERK pathway across cancer types, which warrants further investigation. Nevertheless, the fact that similar patterns are conserved across two distinct cancer types suggests that MICAL2 may be a common effector in RAS-driven oncogenesis and is not just specific to FN-RMS. Moreover, we also analyzed a publicly available RMS patient dataset, which showed that FN-RMS tumors harboring mutations in either KRAS, NRAS, or HRAS appear to exhibit an inclination for higher *MICAL2* expression compared to normal skeletal muscle. This reiterates a possible link between constitutive RAS activation and MICAL2 dysregulation in driving FN-RMS aggressiveness, and further studies in larger cohorts with defined RAS mutational status will provide a clearer picture regarding the clinical relevance of this association.

In summary, this study identifies MICAL2 as a novel contributor to FN-RMS pathogenesis by demonstrating that its silencing simultaneously suppresses oncogenic signaling and enhances myogenic integrity. Consolidating transcriptomic, proteomic, and functional data across murine and human models puts forth MICAL2 as a potential link between cytoskeletal dysregulation and tumor aggressiveness. Integrating the findings presented here with prior studies on MICAL2’s role in myogenic commitment underscores the importance of maintaining its moderate expression to navigate between loss of viability and cancer development.

## Materials and methods

### Culture of murine and human cell lines

Human MABs were cultured on collagen-coated surfaces in Iscove’s Modified Dulbecco’s Medium supplemented with 15% fetal bovine serum (FBS), 1% penicillin/streptomycin (P/S), 1% l-glutamine, 1% non-essential amino acids, 1% sodium pyruvate, 1% insulin-transferrin-selenium, 1:10,000 basic fibroblast growth factor, and 0.2% beta-mercaptoethanol, under standard incubator conditions (humidified atmosphere, 37 °C, 5% CO_2_) [33]. Murine myoblast cells (C2C12) were cultured under standard incubator conditions with high glucose Dulbecco’s Modified Eagles Medium (DMEM-HG) supplemented with 10% FBS, 1% P/S, 1% sodium pyruvate, and 2 mM l-glutamine. Human RMS cell lines RD18, RH30, and RH41, as well as murine RMS cell lines KMR19, KMR46, and M3-9M were cultured under standard incubator conditions with DMEM-HG supplemented with 10% FBS, and 1% P/S. Cell passaging was done every 2-3 days when the cells were approximately 80% confluent and regularly tested for mycoplasma. RD18 was kindly gifted by Professor Alessandro Fanzani (University of Brescia, Italy), RH41 and RH30 were received from Texas Tech University Health Sciences Center-Children’s Oncology Group, and KMR19 and KMR46 were obtained from Professor Jason Yustein (Baylor College of Medicine, Houston, TX, USA).

### Plasmid construction

Generation of the plasmid constructs that were investigated in the present study was performed by the lab of Professor Rik Gijsbers from the Department of Molecular Virology and Gene Therapy at KU Leuven. The plasmids contain short hairpin sequences obtained from Merck’s predesigned shRNA database, which were converted into a miRNA structure (hence called sh-miRNA) and target different regions of the *Mus musculus* (*Mm*) *Mical2* mRNA. Target sequences of the shRNA are GCAGTGCCCTAACATTTCCAA, GCTCAGGTTTATCCCAGTGAA and GCCAGCATCTGTAACCAATTA for sh-miRNA1 -2 and -3 MmMical2, respectively. Specifically, sh-miRNA1-MmMical2 and sh-miRNA3-MmMical2, which represent sh-miR1 and sh-miR3, respectively, bind to the untranslated region while sh-miRNA2-MmMical2 (sh-miR2) binds to the coding region of the sequence. An additional plasmid, which is designated as sham, contains a miRNA targeting FLuc, rather than *Mm Mical2* mRNA, as a non-targeting control.

### Transient in vitro transfections

In preparation for transient transfection experiments with the aim of silencing MICAL2 expression, cells were plated 24 h prior to transfections at a density of 12,000 cells/cm^2^. These experiments were performed on the FN-RMS cell lines by employing lipofectamine 2000 (Invitrogen) as the transfection reagent with Opti-MEM (Gibco Thermo Fisher) as the basal medium. A solution made of Opti-MEM medium with 1:20 lipofectamine 2000 was incubated at room temperature (RT) for 5 min. In parallel, the same volume of Opti-MEM was incubated for 5 min with either a concentration of 0.00125 µg/µL of sh-miR1/2/3 or sh-miR-FLuc (sham), or 1:15 esiRNA targeting MICAL2 (Mission^®^ esiRNA Sigma Aldrich). The Opti-MEM solution with either esiRNA or plasmid was put in the lipofectamine solution and incubated together for 20 min at RT. Meanwhile, culture medium was removed, and cells were washed once with phosphate-buffered saline (PBS). The resultant lipofectamine-plasmid/esiRNA solution in Opti-MEM then substituted the original culture medium, with the exception of the negative control (UT), which contained only Opti-MEM, and the lipofectamine control (Lipo C-), which contained Opti-MEM with lipofectamine. Transfection lasted for 9 h under standard incubation conditions as previously described and was terminated with the addition of DMEM + 10% FBS + 1% P/S to each well. After which, the cells were allowed to proliferate for a further 72 h (recovery period) before being used for subsequent analyses (IF, WB, and migration assay). The transfection time and recovery period were determined based on prior experiments that demonstrated that 9 h of transfection incubation time yields the most ideal results in terms of cell viability and percentage of transfected cells, and 72 h of proliferation post-transfection produced peak MICAL2 silencing effects [23, 30].

### Immunofluorescence assay

Immunofluorescence assays were performed as previously described [59, 60]. Briefly, cells were fixed using 4% paraformaldehyde (PFA) (Sigma-Aldrich) for 15 min at RT then washed three times with PBS. The fixed cells were then permeabilized using 1% bovine serum albumin (BSA) and 0.2% TritonX-100 in PBS solution for 30 min and subsequently blocked with 10% donkey serum for 1 h. Incubation overnight at 4 °C with primary antibodies against rabbit anti-MICAL2 (1:300) (Santa Cruz sc-135498) and mouse anti-Ki67 (1:300) (BD Biosciences 556003) diluted in 1% BSA in PBS solution was followed by incubation with anti-mouse and anti-rabbit secondary antibodies (1:500) conjugated with 594 or 488 AlexaFluor fluorochromes (Thermo Fisher Scientific Invitrogen™). Nuclei were stained with Hoechst (33342, Thermo Fisher Scientific, 1:10,000) for 1 min, and imaging was done with the Eclipse Ti Microscope via Image-Pro Plus 6.0 software (both Nikon). Quantification and analysis of the obtained images was done with ImageJ software.

### Western blots

Western blots (WB) were done as previously described [61]. Briefly, cell lysates were obtained by using RIPA buffer (Sigma-Aldrich) supplemented with 10 mM sodium fluoride, 0.5 mM sodium orthovanadate, 1:100 protease inhibitor cocktail, and 1 mM phenylmethylsulphonyl fluoride (PMSF). Cell lysates were then sonicated for 30 s with 10% amplitude, left on ice for 15 min, and centrifuged for 15 min at 13,000 rpm at 4 °C. For all samples, 40 μg of protein were isolated for heat-denaturation in sample-loading buffer (50 mM Tris–HCl, pH 6.8, 100 mM DTT, 2% SDS, 0.1% bromophenol blue, 10% glycerol) using ThermoCycler for 10 min at 99 °C. Tris-glycine-SDS-polyacrylamide gels were used to resolve proteins via electrophoresis that were then transferred to nitrocellulose membranes (Protran, Nitrocellulose membrane, Sigma-Aldrich) and blocked using TRIS-buffered saline (TBS) with 0.05% TWEEN and 5% skim milk powder (Sigma-Aldrich). Subsequently, the membranes were left for overnight incubation with primary antibodies (Table S6) diluted in 2.5% skim milk. Incubation with secondary horseradish peroxidase-conjugated antibodies (BioRad) diluted 1:5000 in 2.5% milk took place the following day. Detection of protein bands using a GelDoc chemiluminescence detection system (BioRad) was done by incubating membranes with SuperSignal Dura Chemiluminescence substrate (Thermo Fisher Scientific), and the Quantity One software (BioRad) was utilized for quantification. Relative densitometry was calculated by normalizing the protein band against the background and α-TUB or GAPDH (housekeeping protein). Uncropped WB membranes are provided as Supplementary Figures.

### XTT assay

Following manufacturer’s protocols, the TACS® XTT cell proliferation assay kit (R&D Systems) was used to assess the metabolic activity of KMR19 and KMR46 after transfection experiments at five separate time points of 0 h (immediately before transfection), 9 h (at the end of transfection), 24 h, 48 h, and 72 h. Cells were seeded on 96-well plates at a density of 12,000 cells/cm^2^ (~4000 cells/well). Transfection medium was replaced with culture medium prior to incubation with XTT working solution. XTT, a tetrazolium salt, is cleaved by mitochondrial enzymes to produce formazan, which displays an orange coloring whose absorbance was measured at 450 nm using a microplate reader (BioTek) after a 4 h incubation time. Relative absorbances were calculated by normalizing against absorbance readings of wells containing XTT working solution only.

### Transwell migration assay and crystal violet staining

Transfected cells (see transfection protocol above) were detached from 6-well plates using 0.25% Trypsin-EDTA solution, and pelleted cells were resuspended in serum free Opti-MEM medium. 50,000 cells suspended in 100 µL of Opti-MEM were seeded in each 24-well transwell insert (Costar™, Corning Incorporated, 6.5 mm diameter, 8 µm pore size, polyester (PET) membrane) while 800 µL of culture medium was added into the lower well to contact the membrane of the inserts. Incubation under standard conditions lasted for 24 h to permit the cells to migrate. The following day, excess medium and non-migrated cells were gently removed from the inserts using a cotton swab, and the culture medium from the lower well was also removed. Inserts and lower wells were washed twice with PBS, after which, fixation took place by incubating the inserts with 4% PFA added to the top of the membrane and submerged in the lower well. PBS washes were done subsequently and were followed by a 10 min incubation of the inserts immersed in 70% ethanol. Membranes were left outside to dry for approximately 10 min after excess ethanol was removed with a cotton swab. Finally, the inserts were stained with 1% crystal violet for 15 min at RT. Following the staining, excess crystal violet was removed with a pipette tip or cotton swab, and the inserts were carefully dipped into Milli-Q water to further remove the remaining dye. Images were subsequently acquired using a DMi1 inverted microscope (Leica) with LAS EZ software (Leica).

### Transduction of KMR19 cells with LV_Luc2-HygroR and LV_TetON miRFP670miR1/2-MmMical2/eGFP-PuroR vectors

Cloning of the different miR transfer plasmids into inducible TET-ON constructs and all subsequent steps for preparing the finalized lentiviral vectors and transduced cells were handled by the lab of Professor Rik Gijsbers (Department of Molecular Virology and Gene Therapy, KU Leuven). KMR19 cells were stably transduced at low transduction efficiency with a lentiviral vector (LV) encoding firefly luciferase (FLuc) driven by a universal EFS (EF1a-short) promoter (LV_EFS-FLuc-IRES-HygroR) and subsequently selected with Hygromycin. The resulting cells were next transduced with a second LV encoding a red fluorescent protein (miRFP670) and the respective sh-miR1 or 2, or a sh-miR targeting eGFP as the sham control, driven by a Tet-inducible promoter (TetON). The resulting cells selected from these two transductions were therefore, positive for firefly luciferase (FLuc + ), contain the respective miR sequence targeting *Mm Mical2* mRNA or eGFP, and can be induced to switch off MICAL2 expression upon doxycycline administration. However, vector integration site and copy number were not directly assessed.

### Bioluminescence imaging for tumor engraftment and progression

The complete in vivo experimental timeline is provided in Fig. S5A. The experimental groups are designated as follows: 1) healthy negative controls; 2) positive controls (FLuc+ tumor); 3) sham (FLuc+ tumor with LV_TetON-miRFP670-miR-eGFP-PuroR); 4) sh-miR1 (FLuc+ tumor with LV_TetON-miRFP670-miR1-MmMical2-PuroR); and 5) sh-miR2 (FLuc+ tumor with LV_TetON-miRFP670-miR2-MmMical2-PuroR). 9-week-old C57BL/6 mice were used for these experiments, and each mouse was injected with 1) saline, 2) 1 × 10^5^ KMR19 FLuc+ cells, 3) 1 × 10^5^ KMR19 FLuc+ cells with LV_TetON-miRFP670-miR-eGFP-PuroR, 4) 1 × 10^5^ KMR19 FLuc+ cells with LV_TetON-miRFP670-miR1-MmMical2-PuroR, or 5) 1 × 10^5^ KMR19 FLuc+ cells with LV_TetON-miRFP670-miR2-MmMical2-PuroR. Each group is composed of six mice (three males and three females). Under anesthesia (10% Ketamine, 5% Xylazine, 85% saline solution, 100 μL intraperitoneal dose/10 g mouse), the mice were injected intramuscularly in bilateral hindlimbs in TA, GN and Q muscles with the cells suspended in 200 μl of saline water. Following these injections, tumor cells were allowed to proliferate and engraft for 5 days. To administer doxycycline, it was diluted in artificially flavored gelatin (2 mg/mL), which was given to all the mice throughout the course of the experiment beginning on day 0 (daily fresh cube of gelatin containing 6 mg doxycycline per cage) [62]. Monitoring tumor progression using BLI began on day 2, and starting on day 5, was performed every 2 days by placing the mice in the flow chamber of an IVIS® Spectrum machine. The mice were anesthetized via isoflurane inhalation in an open system and injected intraperitoneally with 126 mg/kg of D-luciferin [30]. Subsequently, Living Image® software was utilized to capture multiple frames to determine optimal exposure time to maximize signal. The software was also used to calculate total flux (photons/sec) by comparing the same region of interest (ROI) for all animals, which were normalized on background signals and non-injected mice.

### Treadmill exhaustion test

The five experimental groups of mice (see above) underwent treadmill exhaustion tests that were carried out starting from day 0, before injections and administration of doxycycline began, and every five days thereafter. During each treadmill test, electric shock pulses were presented with an intensity of 1.22 mA and frequency of 3 Hz [23]. After introducing mice to the treadmill belt, they were given 5 min to adapt to the environment with the motor speed set to zero. Training time of 2 min at a speed of 4 m/min was provided, followed by the test, which also started with a 4 m/min speed and maintained a constant uphill incline of 20°. The speed was increased by 1 m/min every minute until reaching the maximum speed of 27 m/min. The test continued until mice reached exhaustion, which is characterized by a ≥ 10 s rest of the mice on the electric grid. The mice were weighed immediately following each run, and speed (m/min), distance (m), and time (min and sec) were recorded to calculate the work of each run in joules (J). The formula applied for these calculations is the following: work (J) = body mass (kg) x gravity (9.81 m/sec^2^) x vertical speed (m/sec x angle) x time (sec). Power (W) was then calculated by dividing the work by the times obtained from the tests.

### RNA-Seq

RNA isolation was done with the PureLink® RNA mini kit (Ambion), which was followed by DNase I treatment with the Turbo^TM^ DNA-free kit (Thermo Fisher Scientific). RNA ( > 10 μg) samples were verified and processed by the Genomics Core (KU Leuven – UZ Leuven). RNA-sequencing libraries were constructed with the Lexogen library. Samples were indexed with unique adapters and pooled for single read (50 bp) sequencing in an Illumina HiSeq 4000 system. Quality control of raw reads was performed with FastQC v0.11.7 (https://www.bioinformatics.babraham.ac.uk/projects/fastqc/). Adapters were filtered using Trimmomatic v0.39 [63]. Splice-aware alignment was carried out using Hisat2 against the reference genome using the default parameters [64]. Quantification of reads per gene was conducted using FeatureCounts from the Subread package [65]. Genes with fewer than 1 count across all samples were excluded prior to analysis. Differential expression analysis was performed using DESeq2 v1.44.0 in R [66]. DEGs were defined by |log_2_ FC | ≥ 1 and adjusted *p*-value < 0.05, and reported *p*-values underwent adjustment for multiple testing using the Benjamini-Hochberg procedure to control the false discovery rate (FDR). GO/KEGG overrepresentation analyses were performed using clusterProfiler v4.12.6 with the org.Mm.eg.db annotation database and all detected genes as the background universe.

### Mass spectrometry analysis

Muscle tissues were lysed in RIPA (see WB section of materials and methods above). 30 µg of the protein amount were mixed with 10% SDS and 50 mM TEAB. Sample preparation was conducted using the S-Trap micro spin column (ProtiFi) following the manufacturer’s instructions. After cell lysis and mixture with SDS-based buffer, samples were reduced using 5 mM tris(2-carboxyethyl)phosphine (TCEP), alkylated by 20 mM iodoacetamide (IAA), and further denatured by 2.5% (v/v) phosphoric acid. The samples were then diluted with 165 µl of binding buffer (90% methanol in 100 mM TEAB). After loading the samples onto S-trap micro S-trap columns and centrifuging for 30 s at 10,000 xg, the columns were washed three times with 150 µl binding buffer. Trypsin at a 1:30 ratio (enzyme:protein) in 20 µl of 50 mM TEAB was added and incubated overnight at 37 °C. Peptides were eluted in a sequential manner using centrifugation steps (1 min at 10,000 xg): first with 40 µl 50 mM TEAB, then with 40 µl 0.2% formic acid (FA) in water, and finally, with 40 µl water/acetonitrile (ACN) (50/50, v/v). The eluted peptides were completely dried by speed vacuum centrifugation.

The tryptic peptides were reconstituted in 0.5% ACN + 99.5% H_2_O containing 0.1% formic acid (FA). Approximately 500 ng were loaded onto an Evotip and analyzed using an Evosep One LC system (EVOSEP) coupled to a ZenoTOF 7600 mass spectrometer equipped with an Optiflow source (SCIEX). 60SPD (samples per day) method with a 21 min gradient was employed using an EV1109 Performance column (8 cm×150 µm, 1.5 µm). Operating in positive mode, the mass spectrometer utilized an OptiFlow V source with a spray voltage of 4500 V and employed SWATH mode. The SWATH acquisition consisted of 65 variable-size windows covering a precursor ion mass range of 400-1250 m/z.

All raw data from the ZenoTOF 7600 system (.wiff) were acquired using SCIEX OS and processed with DIA-NN 1.9 (Data-Independent Acquisition by Neural Networks) in library-free mode [67, 68]. Spectra were compared against the Mus Musculus reference proteome database (https://www.uniprot.org/proteomes/UP000000589). Enzyme specificity allowed cleavage C-terminal to arginine and lysine, permitting cleavage at proline bonds with a maximum of two missed cleavages. Oxidation of methionine residues and N-terminus acetylation were considered as variable modifications and cysteine carbamidomethylation as a fixed modification. Match between runs (MBR) was enabled and MS1 and MS2 were set to 20ppm. The neural network classifier was set to single-pass mode, and protein inference was based on genes. The remaining default settings of DIA-NN were kept, utilizing Heuristic protein inference, QuantUMS (high precision) mode with retention time (RT)-dependent normalization enabled, and library generation based on IDs, RT & IM profiling [69]. The DIA-NN protein group output was further evaluated using in-house R scripts. DIA abundance data were converted to log_2_ scale, samples were grouped by conditions, and missing values were imputed using the approach “bpca”, Bayesian missing value imputation. Protein-wise linear models combined with empirical Bayes statistics from the limma package in R Bioconductor were used for the differential expression analyses [70]. A cutoff of the adjusted *p*-value of 0.05 (Benjamini-Hochberg method) along with a |log_2_ fold change| of 1 were applied to determine significantly regulated proteins in each pairwise comparison. GO and KEGG overrepresentation analyses were performed using clusterProfiler v4.12.6 with the org.Mm.eg.db annotation database and all detected proteins as the background universe. GSEA was carried out with Pathway-Analyst (https://analyst-suites.org/apps/pathway-analyst/).

### Statistical analysis

Sample Size Calculator (https://www.psychologie.hhu.de/arbeitsgruppen/allgemeine-psychologie-und-arbeitspsychologie/gpower; parameters: power, 0.80; alpha, 0.05) was employed to calculate sample sizes for in vivo experiments, and when applicable, sample size analysis was done according to average values derived from preliminary optimization/validation trials. GraphPad Prism 10.2.0 (GraphPad Software, San Diego, CA, USA) was utilized for all statistical analyses and generation of graphs for the results section. One-way ANOVA or two-way ANOVA tests were used for comparison of multiple factors, as appropriate. Photos obtained using fluorescent microscopes were analyzed and edited with ImageJ Version 1.53. Data is reported as mean ± standard deviation (SD). Specific information regarding the statistical test used and other relevant details for each independent experiment are detailed in the figure legends.

## Supplementary information

Supplementary Information

Original Blots

## Data Availability

Bulk RNA-seq data have been deposited in GEO under accession code GSE278087, and mass spectrometry proteomics data have been deposited to the ProteomeXchange Consortium via the PRIDE partner repository with the dataset identifier PXD056474. All other relevant data are available from the corresponding author upon reasonable request.
