# Supplementary material for: Unraveling the dual roles of MICAL2 in skeletal muscle physiology and fusion negative rhabdomyosarcoma tumor progression
Source: Cell Death Dis. 2026 Jul 7;17(1):845. doi: 10.1038/s41419-026-09029-w (PMC13623957; doi:10.1038/s41419-026-09029-w)
Supplement: Supplementary file 1 [file 41419_2026_9029_MOESM1_ESM.pdf]

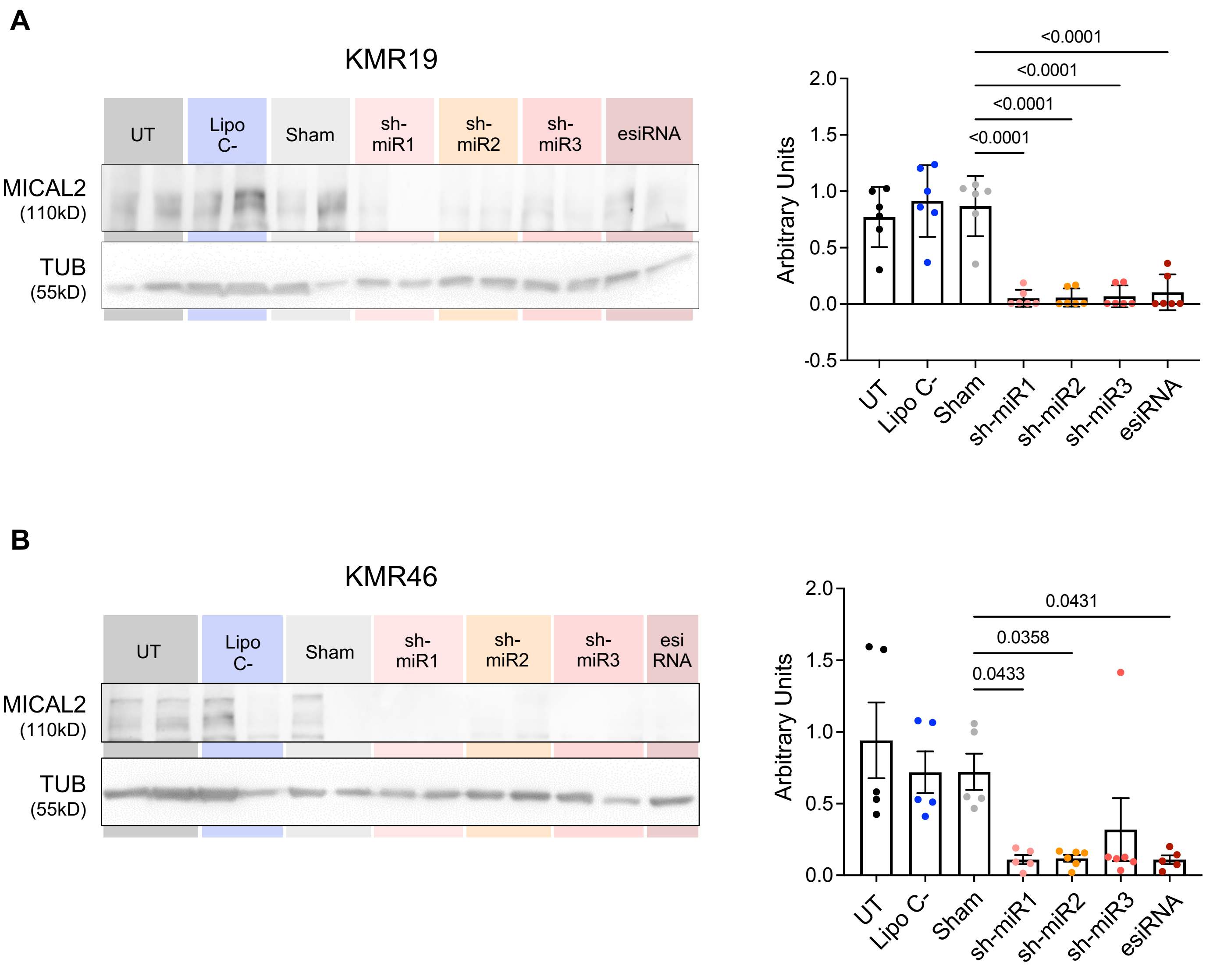

Supplementary Fig. S1 Validation of MICAL2 silencing in KMR19 and KMR46 murine FN-RMS cells. FN-RMS cells were collected 72h post-transfection with sham (non-targeting control), MICAL2-targeting plasmid constructs (sh-miR1, sh-miR2, sh-miR3), or commercial esiRNA (positive control). Untreated (UT) and lipofectamine-treated cells (Lipo C-) were included as negative controls. **A,B** WB analyses of MICAL2 in (**A**) KMR19 and (**B**) KMR46 cells. Band intensities were normalized to TUB and presented as arbitrary units. Data are representative of six independent experiments (n = 6) and expressed as mean ± SD. Statistical analysis was performed using one-way ANOVA with Dunnett's multiple comparisons test.

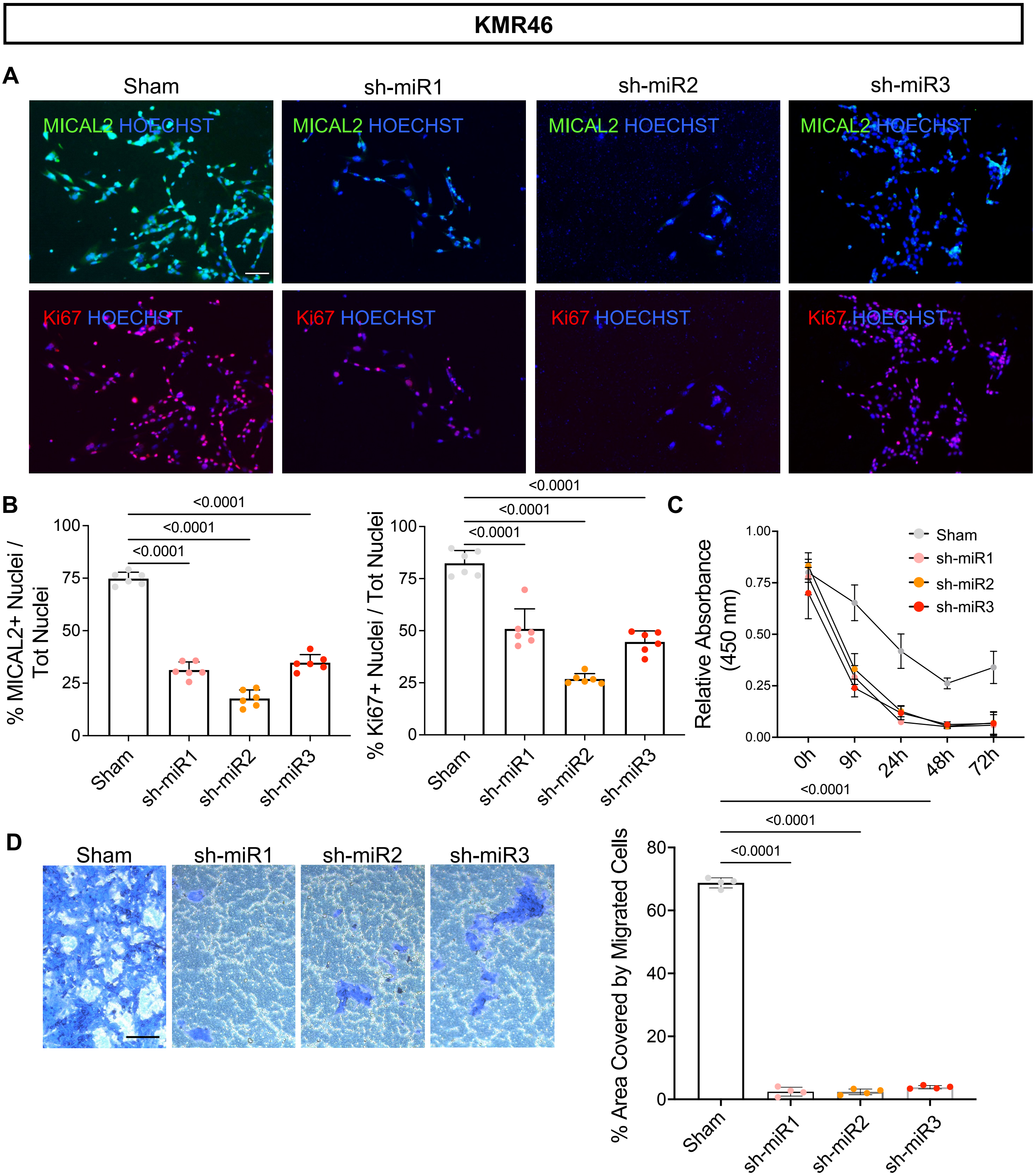
KMR46
A
Sham
sh-miR1
sh-miR2
sh-miR3
MICAL2 HOECHST
Ki67 HOECHST
B
% MICAL2+ Nuclei / Tot Nuclei
<0.0001
<0.0001
<0.0001
0
25
50
75
100
Sham
sh-miR1
sh-miR2
sh-miR3
% Ki67+ Nuclei / Tot Nuclei
C
Relative Absorbance (450 nm)
0.00
0.25
0.50
0.75
1.00
0h
9h
24h
48h
72h
Sham
sh-miR1
sh-miR2
sh-miR3
D
% Area Covered by Migrated Cells
0
20
40
60
80

Supplementary Fig. S2 MICAL2 silencing reduces proliferative and migratory capacities in murine KMR46 FN-RMS cells. A Representative immunofluorescence images for MICAL2 (green) and Ki67 (red) in KMR46 FN-RMS cells 72h post-transfection with MICAL2-targeting plasmid constructs. Nuclei were stained with HOECHST (blue). Scale bar = 50 µm. **B** Relative quantification of MICAL2 (left) and Ki67 (right) shown as % expression (number of positive nuclei over total number of nuclei). Data are representative of six independent experiments (n = 6) and expressed as mean ± SD. Statistical analysis was performed using one-way ANOVA with Dunnett's multiple comparisons test. **C** XTT assessment of metabolic activity in transfected KMR46 cells. Absorbance measurements were recorded at 0, 9, 24, 48, and 72h post-transfection. Data are representative of three independent experiments (n = 3) and expressed as mean ± SD. Statistical analysis was performed using two-way ANOVA with Dunnett's multiple comparisons test (Table S1). **D** Representative images of crystal violet staining of transfected KMR46 cells after 24h migration in a Boyden chamber assay (left). Quantification is expressed as the % of the area covered by migrated KMR46 cells (right). Scale bar = 100 µm. Data are representative of four independent experiments (n = 4) and expressed as mean ± SD. Statistical analysis was performed using one-way ANOVA with Dunnett's multiple comparisons test.

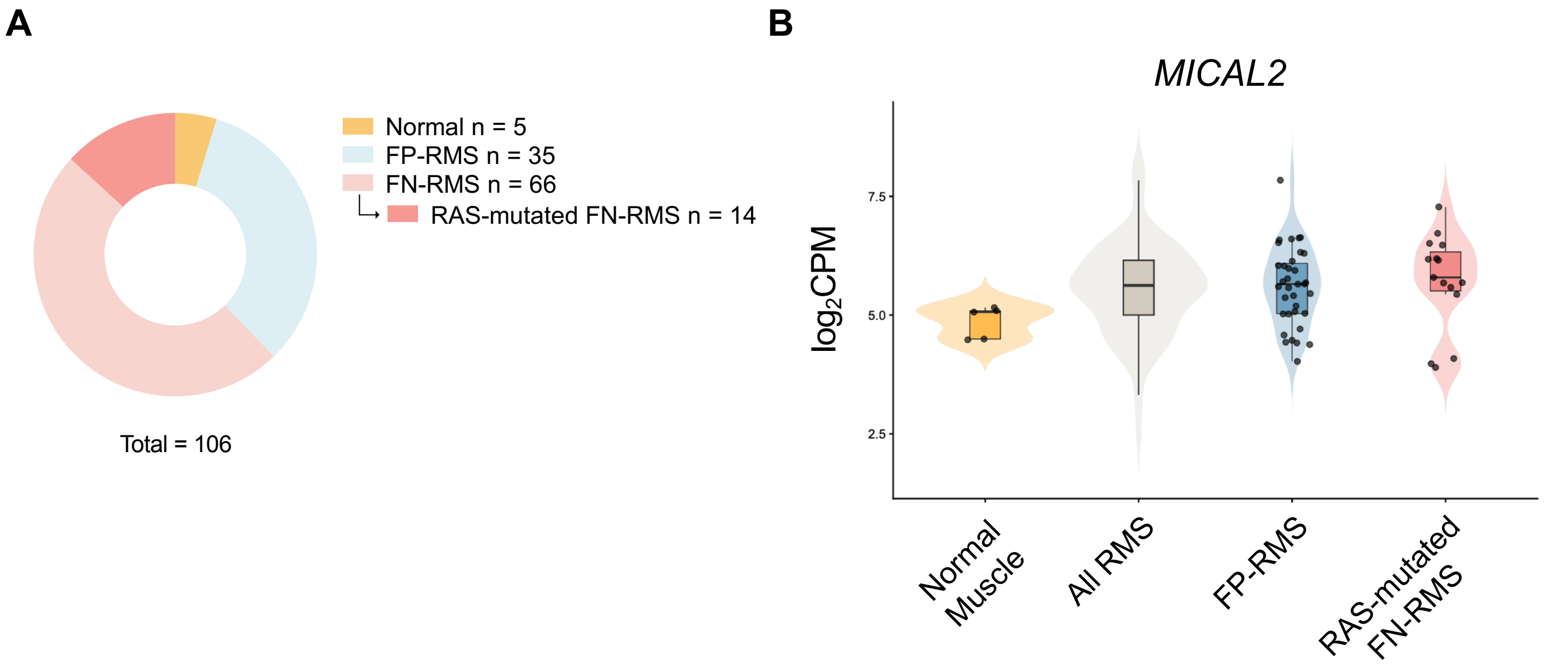

Supplementary Fig. S3 MICAL2 displays aberrant expression in human primary RMS samples. Analysis of RNA-seq dataset from GSE108022. **A** Donut chart of the distribution of sample types in the dataset. In total, there are 106 samples, consisting of 5 normal muscles, 35 FP-RMS patient samples, and 66 FN-RMS patient samples (14 of which, are RAS-mutated). **B** Violin plots of $\log_2$ transformed counts per million ($\log_2$CPM) expression level of *MICAL2* in normal muscle, across all RMS samples, within FP-RMS, and within RAS-mutated FN-RMS. Points represent individual samples. Expression values were obtained using the limma-voom pipeline with TMM library-size normalization (edgeR).

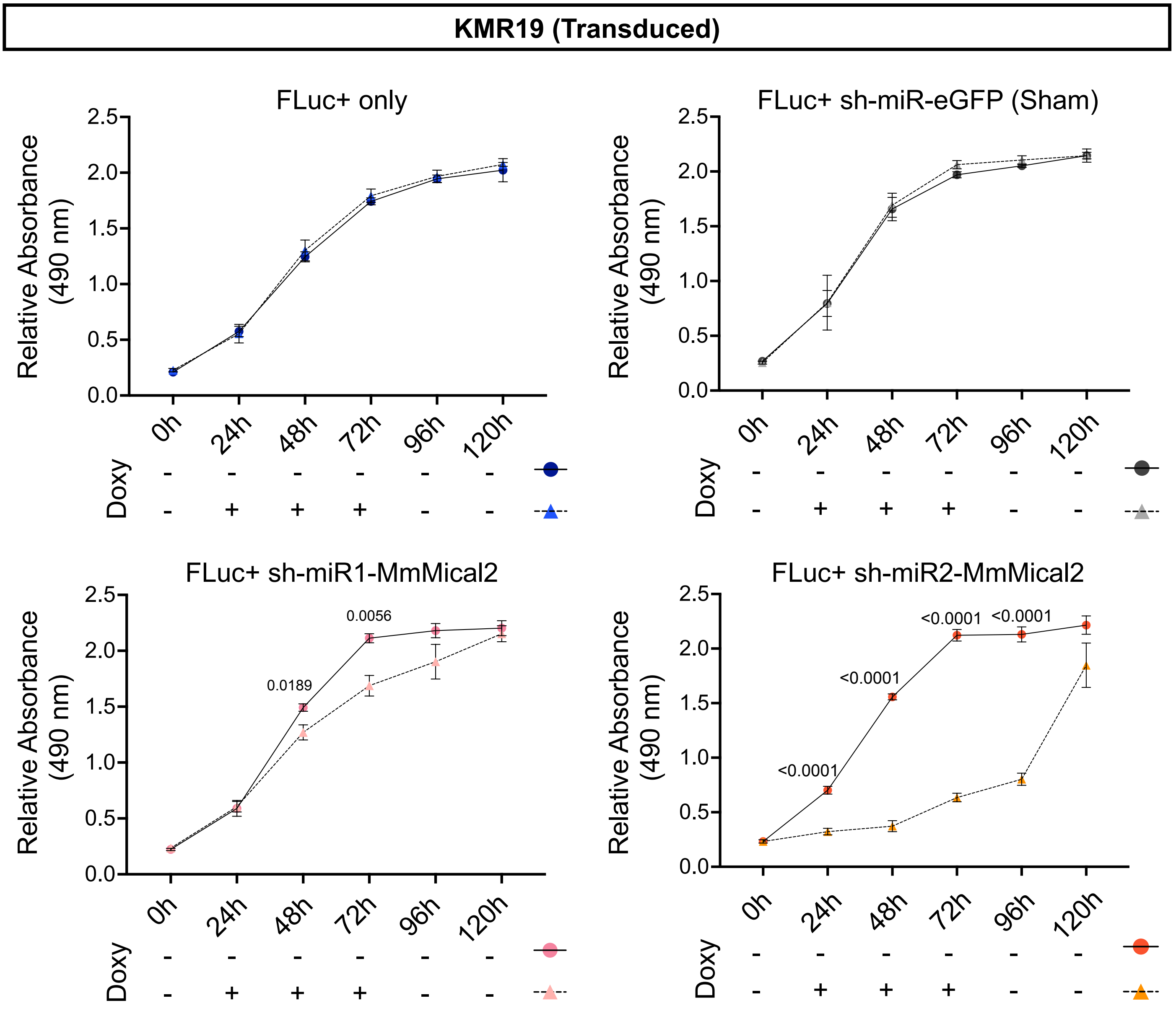

Supplementary Fig. S4 Transduced KMR19 cells exhibit reversible changes in metabolic activity upon doxycycline exposure. XTT assays of transduced KMR19 cells: FLuc+ only, FLuc+ with sh-miR-eGFP (Sham), and FLuc+ with sh-miR1/2-MmMical2. + and - symbols indicate doxycycline presence during the interval preceding each timepoint measurement. 0h represented baseline (one day after seeding cells), and measurements were recorded at 24, 48, 72, 96, and 120h. Data are representative of three independent experiments (n = 3) and expressed as mean ± SD. Statistical analysis was performed using two-way repeated measures ANOVA with Šídák's multiple comparisons test (Table S2).

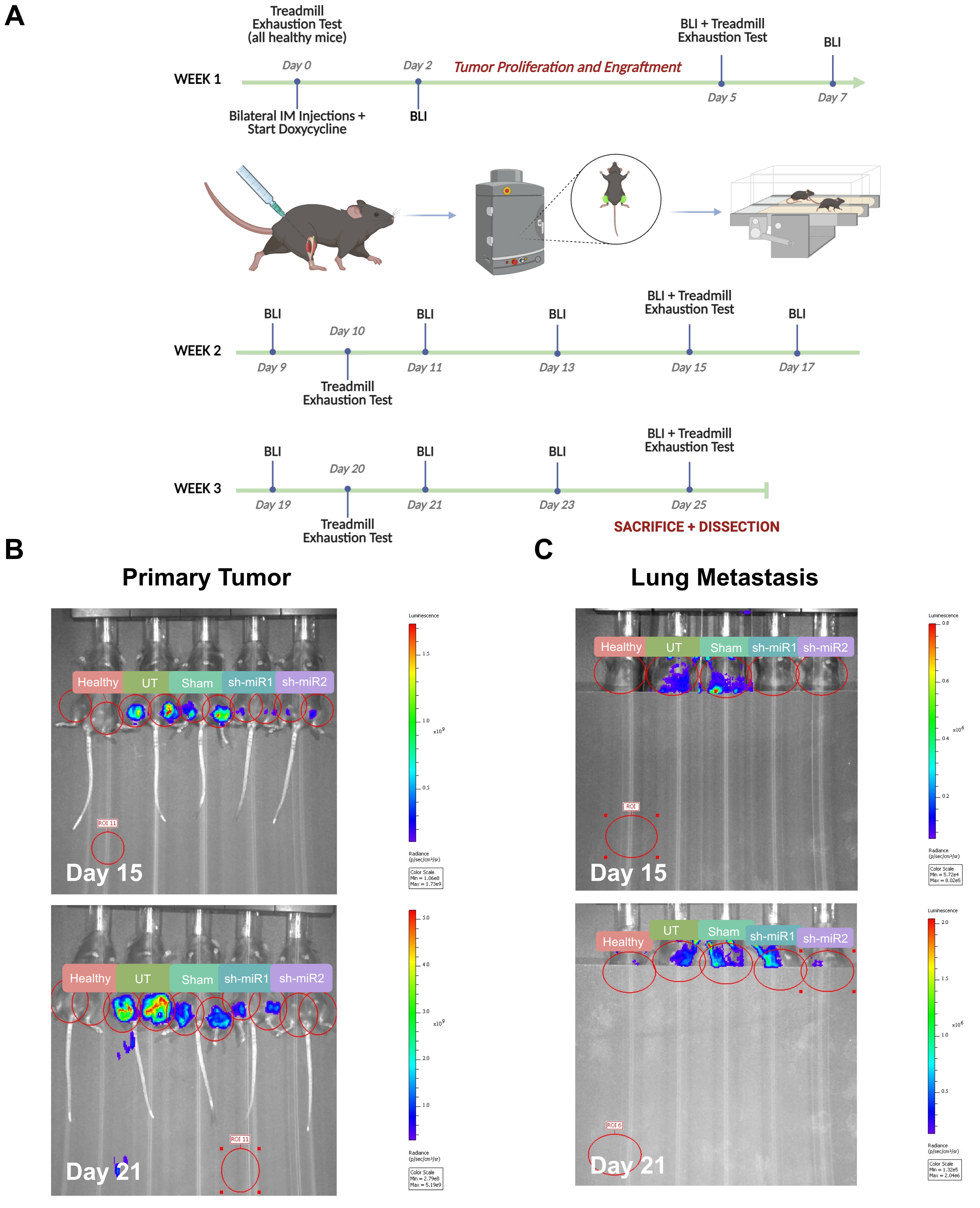

Supplementary Fig. S5 Primary tumor progression and metastasis formation capacities are reduced upon MICAL2 silencing in FN-RMS tumor-bearing mice. A Schematic representation outlining the timeline of *in vivo* experiments. **B,C** Representative bioluminescent images of healthy, UT, sham, sh-miR1, and sh-miR2 tumor-bearing mice. Images were taken on days 15 and 21 with (**B**) representing primary tumors and (**C**) representing tumor metastasis to the lungs.

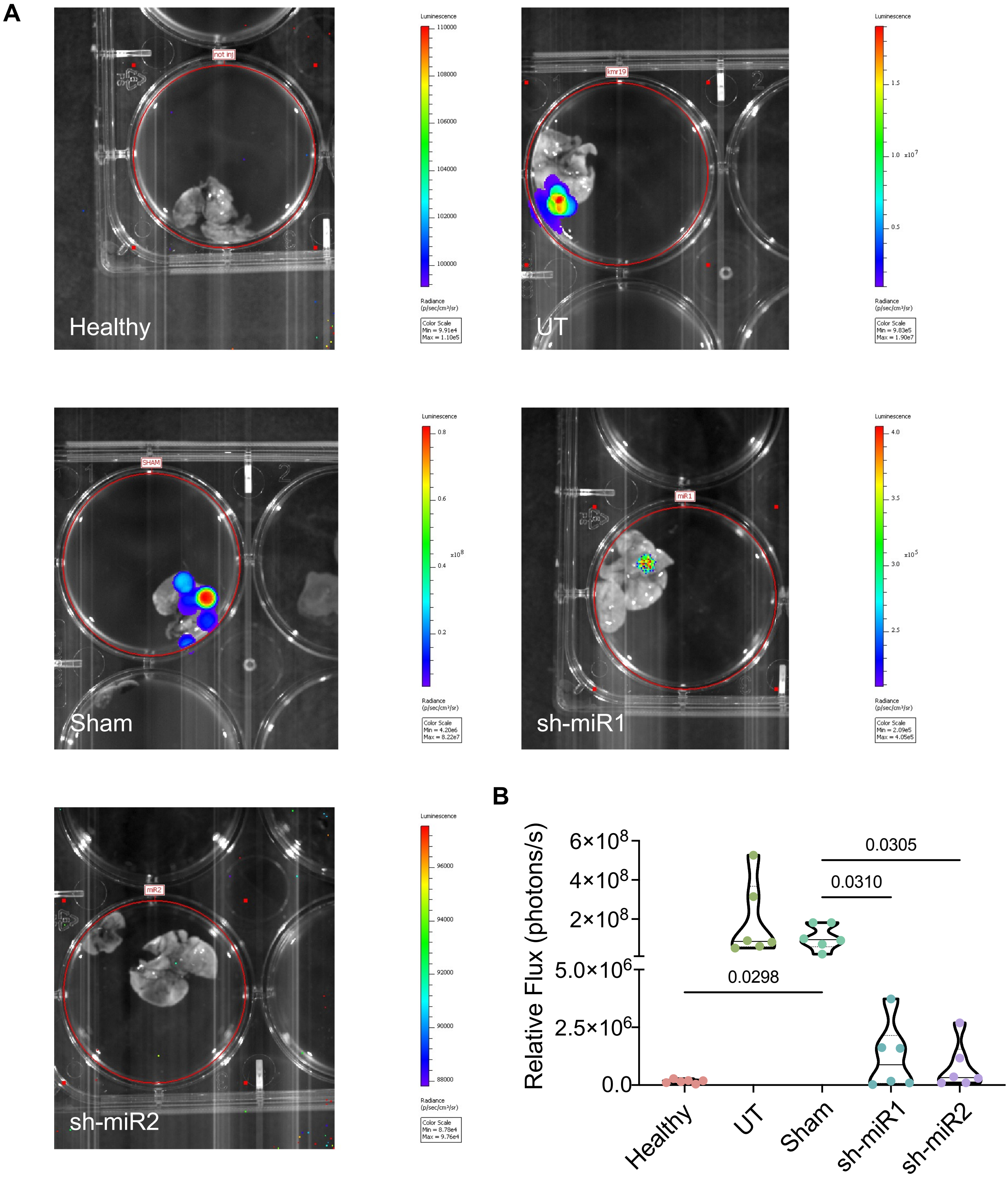

Supplementary Fig. S6 *Ex vivo* metastatic cells are drastically diminished upon MICAL2 silencing in FN-RMS tumor-bearing mice. A Representative bioluminescent images of *ex vivo* lungs explanted from healthy, UT, sham, sh-miR1, and sh-miR2 tumor-bearing mice. Images were taken on day 25, immediately after explant of the organ. **B** Quantification of the BLI signal. Data are expressed as mean ± SD (n = 6 mice per group). Statistical analysis was performed using Brown-Forsythe and Welch ANOVA tests.

A

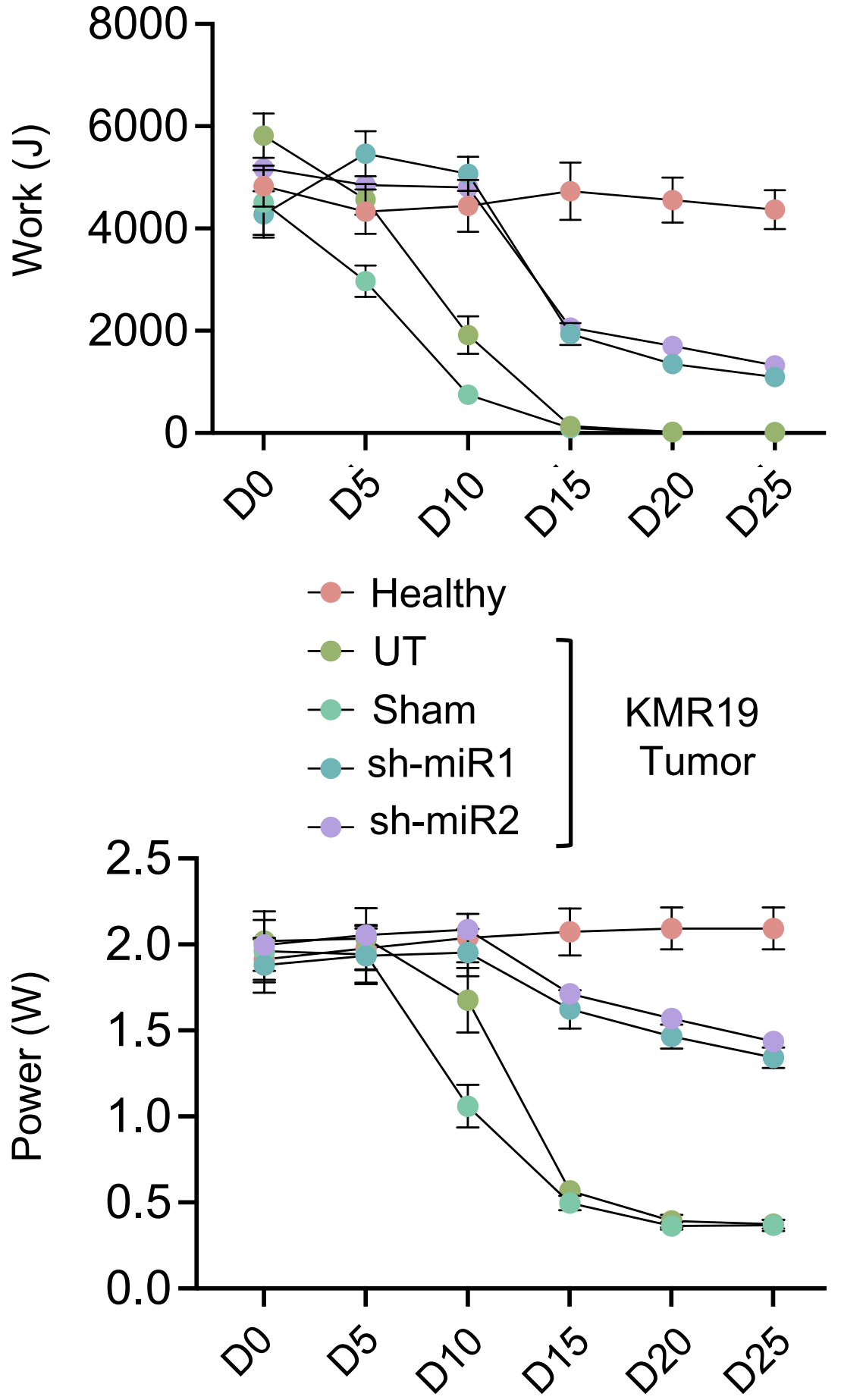

Work (J)	D0	D5	D10	D15	D20	D25
Sham vs Healthy	0.992	0.1635	0.0026	0.0024	0.0008	0.0005
Sham vs UT	0.4822	0.0269	0.1196	0.723	0.9095	0.9841
Sham vs sh-miR1	0.998	0.0085	<0.0001	0.0018	0.0005	0.0001
Sham vs sh-miR2	0.9554	0.0486	0.0006	0.003	0.0029	0.001

Power (W)	D0	D5	D10	D15	D20	D25
Sham vs Healthy	0.9993	0.9998	0.0029	0.0002	0.0001	<0.0001
Sham vs UT	0.9992	0.9953	0.1257	0.8364	0.9554	0.9998
Sham vs sh-miR1	0.9967	>0.9999	0.0049	0.0003	<0.0001	<0.0001
Sham vs sh-miR2	>0.9999	0.9921	0.0115	0.0026	<0.0001	<0.0001

B

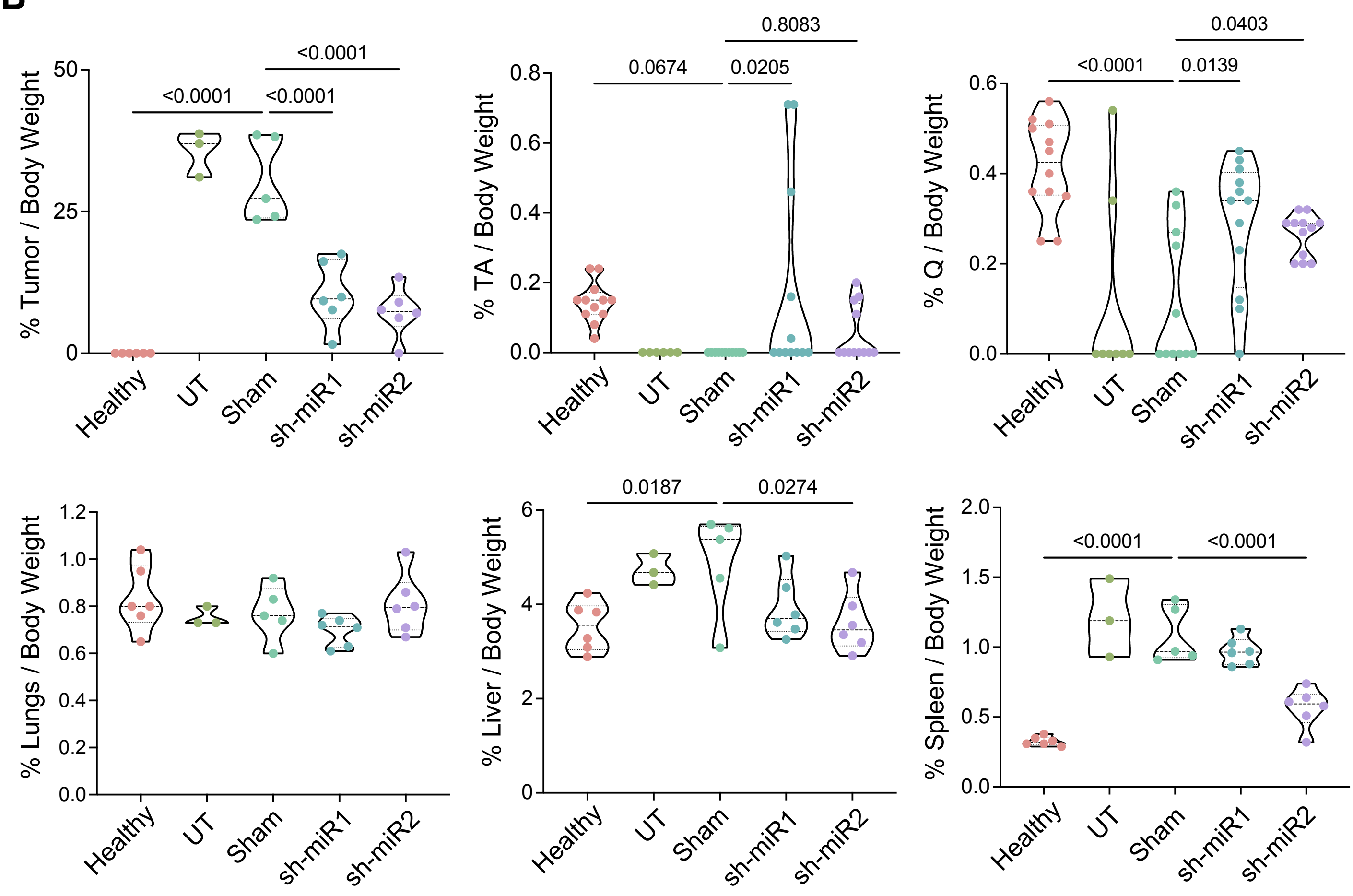

Supplementary Fig. S7 Effects of MICAL2 silencing on physical exertion capacity and organ weight in FN-RMS tumor-bearing mice. A Functional assessment by treadmill exhaustion test performed 0-, 5-, 10-, 15-, 20- and 25-days post-tumor injection. From top to bottom, graphs of Work (J) and Power (W). Data are expressed as mean ± SD (n = 6 mice per group). Statistical analysis was performed using two-way ANOVA with Tukey's multiple comparisons test (Table S4). **B** Weight percentage of tumor mass, TA muscle, Q muscle, lungs, liver, and spleen over total body weight of each individual mouse. Data are expressed as mean ± SD (n = 3-6 mice per group). Statistical analysis was performed using one-way ANOVA with Dunnett's multiple comparisons test.

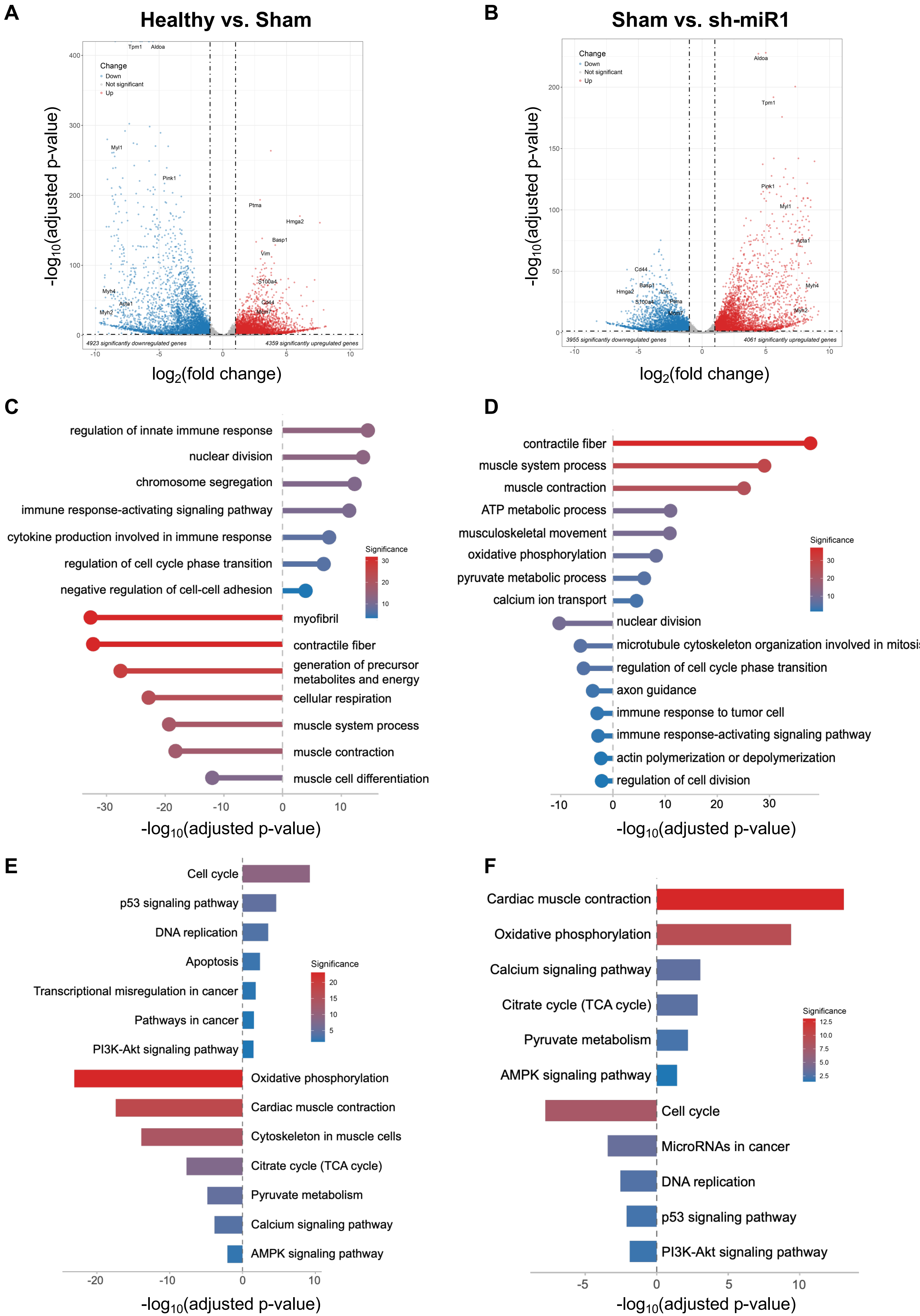

A
Healthy vs. Sham
Change
Down
Not significant
Up
Tpm1
Aldoa
Myl1
Pink1
Ptma
Hmga2
Basp1
Vim
S100a4
Cd44
Mcm7
Myh4
Acta1
Myh2
4923 significantly downregulated genes
4359 significantly upregulated genes
-log10(adjusted p-value)
log2(fold change)
B
Sham vs. sh-miR1
Change
Down
Not significant
Up
Aldoa
Tpm1
Pink1
Myl1
Acta1
Myh4
Myh2
Cd44
Basp1
Hmga2
Vim
S100a4
Ptma
Mcm7
3955 significantly downregulated genes
4061 significantly upregulated genes
-log10(adjusted p-value)
log2(fold change)
C
regulation of innate immune response
nuclear division
chromosome segregation
immune response-activating signaling pathway
cytokine production involved in immune response
regulation of cell cycle phase transition
negative regulation of cell-cell adhesion
myofibril
contractile fiber
generation of precursor metabolites and energy
cellular respiration
muscle system process
muscle contraction
muscle cell differentiation
Significance
-log10(adjusted p-value)
D
contractile fiber
muscle system process
muscle contraction
ATP metabolic process
musculoskeletal movement
oxidative phosphorylation
pyruvate metabolic process
calcium ion transport
nuclear division
microtubule cytoskeleton organization involved in mitosis
regulation of cell cycle phase transition
axon guidance
immune response to tumor cell
immune response-activating signaling pathway
actin polymerization or depolymerization
regulation of cell division
Significance
-log10(adjusted p-value)
E
Cell cycle
p53 signaling pathway
DNA replication
Apoptosis
Transcriptional misregulation in cancer
Pathways in cancer
PI3K-Akt signaling pathway
Oxidative phosphorylation
Cardiac muscle contraction
Cytoskeleton in muscle cells
Citrate cycle (TCA cycle)
Pyruvate metabolism
Calcium signaling pathway
AMPK signaling pathway
Significance
-log10(adjusted p-value)
F
Cardiac muscle contraction
Oxidative phosphorylation
Calcium signaling pathway
Citrate cycle (TCA cycle)
Pyruvate metabolism
AMPK signaling pathway
Cell cycle
MicroRNAs in cancer
DNA replication
p53 signaling pathway
PI3K-Akt signaling pathway
Significance
-log10(adjusted p-value)

Supplementary Fig. S8 RNA-seq analysis results demonstrate suppression of oncogenic signaling concurrent with enhancement of myogenic identity with MICAL2 silencing in FN-RMS tumor-bearing mice. Multi-level transcriptomic analyses are shown for the Healthy vs. Sham comparison (**A**,**C**,**E**) and the Sham vs. sh-miR1 comparison (**B**,**D**,**F**). **A**,**B** Volcano plots of DEGs. **C-F** Selected enrichment terms of (**C**,**D)** GO and (**E**,**F)** KEGG analyses based on DEGs. Bars indicate -$\log_{10}$ (adjusted p-value) for selected terms and pathways: positive values indicate upregulated while negative values indicate downregulated terms and pathways. Color gradient represents the magnitude of statistical significance.

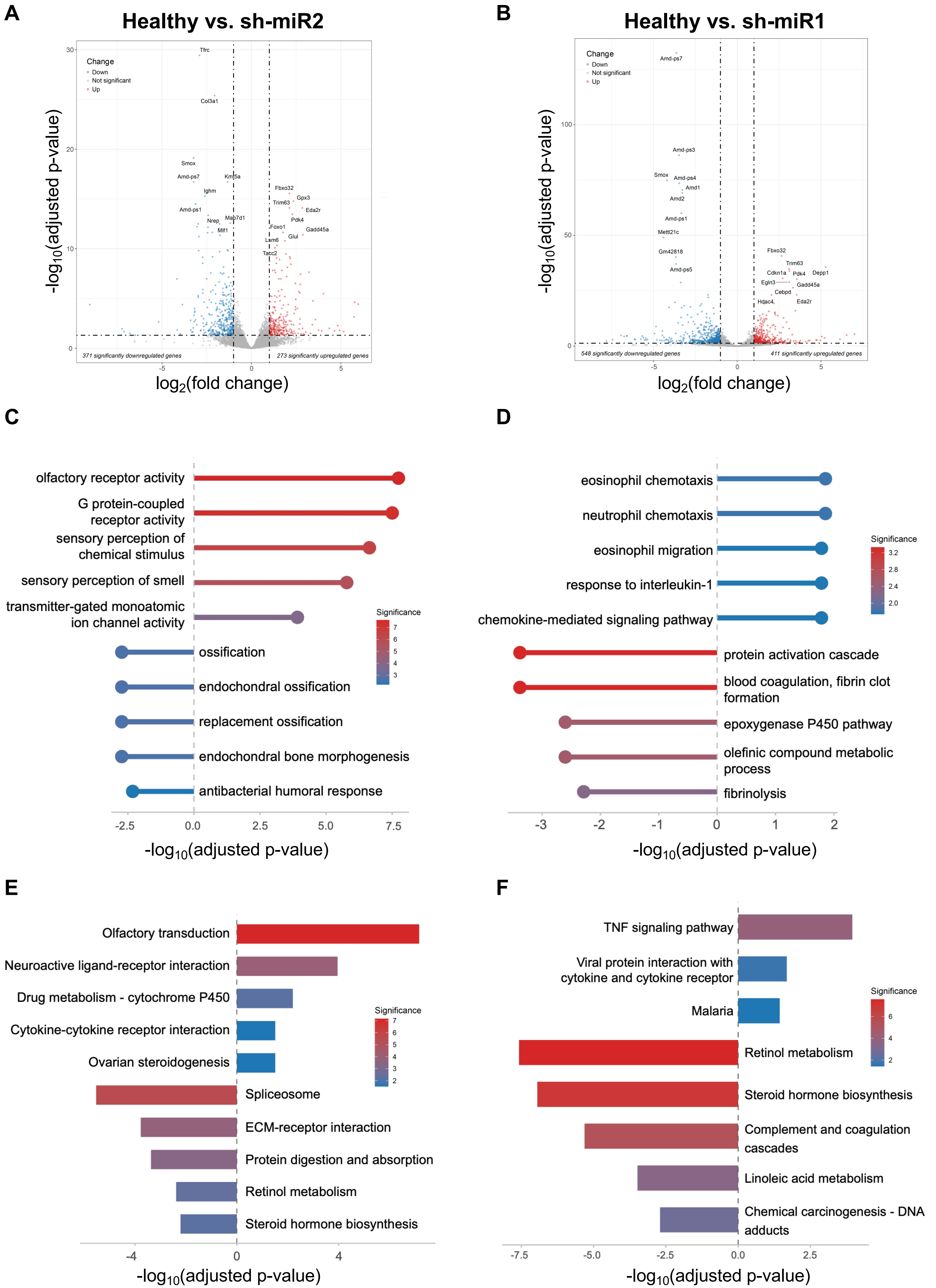
A
Healthy vs. sh-miR2
Change
Down
Not significant
Up
Tfrc
Col3a1
Smox
Amd-ps7
Kmt5a
Ighm
Amd-ps1
Nrep
Map7d1
Mlf1
Fbxo32
Gpx3
Trim63
Eda2r
Pdk4
Foxo1
Gadd45a
Lsm6
Glul
Tacc2
371 significantly downregulated genes
273 significantly upregulated genes
-log10(adjusted p-value)
log2(fold change)
B
Healthy vs. sh-miR1
Amd-ps7
Amd-ps3
Smox
Amd-ps4
Amd1
Amd2
Amd-ps1
Mettl21c
Gm42818
Amd-ps5
Fbxo32
Trim63
Cdkn1a
Pdk4
Depp1
Egln3
Gadd45a
Cebpd
Hdac4
Eda2r
548 significantly downregulated genes
411 significantly upregulated genes
C
olfactory receptor activity
G protein-coupled receptor activity
sensory perception of chemical stimulus
sensory perception of smell
transmitter-gated monoatomic ion channel activity
ossification
endochondral ossification
replacement ossification
endochondral bone morphogenesis
antibacterial humoral response
Significance
D
eosinophil chemotaxis
neutrophil chemotaxis
eosinophil migration
response to interleukin-1
chemokine-mediated signaling pathway
protein activation cascade
blood coagulation, fibrin clot formation
epoxygenase P450 pathway
olefinic compound metabolic process
fibrinolysis
E
Olfactory transduction
Neuroactive ligand-receptor interaction
Drug metabolism - cytochrome P450
Cytokine-cytokine receptor interaction
Ovarian steroidogenesis
Spliceosome
ECM-receptor interaction
Protein digestion and absorption
Retinol metabolism
Steroid hormone biosynthesis
F
TNF signaling pathway
Viral protein interaction with cytokine and cytokine receptor
Malaria
Retinol metabolism
Steroid hormone biosynthesis
Complement and coagulation cascades
Linoleic acid metabolism
Chemical carcinogenesis - DNA adducts
-log10(adjusted p-value)

Supplementary Fig. S9 RNA-seq analysis results demonstrate similarities between healthy control and sh-miR-treated FN-RMS tumor-bearing mice. Multi-level transcriptomic analyses are shown for the Healthy vs. sh-miR2 comparison (**A**,**C**,**E**) and the Healthy vs. sh-miR1 comparison (**B**,**D**,**F**). **A**,**B** Volcano plots of DEGs. **C-F** Selected enrichment terms of (**C**,**D)** GO and (**E**,**F)** KEGG analyses based on DEGs. Bars indicate -$\log_{10}$ (adjusted p-value) for selected terms and pathways: positive values indicate upregulated while negative values indicate downregulated terms and pathways. Color gradient represents the magnitude of statistical significance.

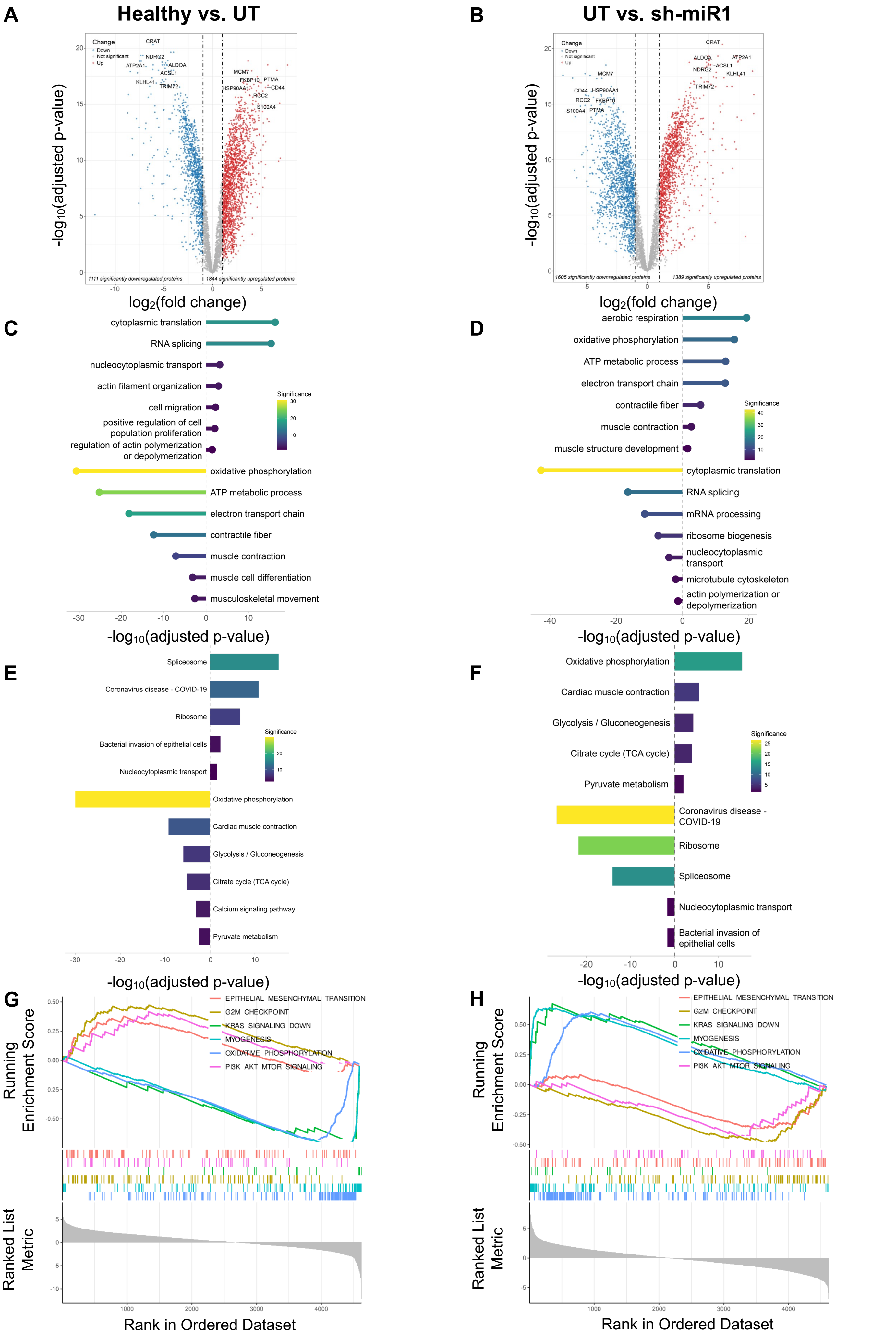
A
Healthy vs. UT
B
UT vs. sh-miR1
-log10(adjusted p-value)
log2(fold change)
1111 significantly downregulated proteins
1844 significantly upregulated proteins
1605 significantly downregulated proteins
1389 significantly upregulated proteins
Change
Down
Not significant
Up
CRAT
NDRG2
ALDOA
ATP2A1
ACSL1
KLHL41
TRIM72
MCM7
FKBP10
PTMA
HSP90AA1
CD44
RCC2
S100A4
C
cytoplasmic translation
RNA splicing
nucleocytoplasmic transport
actin filament organization
cell migration
positive regulation of cell population proliferation
regulation of actin polymerization or depolymerization
oxidative phosphorylation
ATP metabolic process
electron transport chain
contractile fiber
muscle contraction
muscle cell differentiation
musculoskeletal movement
Significance
D
aerobic respiration
oxidative phosphorylation
ATP metabolic process
electron transport chain
contractile fiber
muscle contraction
muscle structure development
cytoplasmic translation
RNA splicing
mRNA processing
ribosome biogenesis
nucleocytoplasmic transport
microtubule cytoskeleton
actin polymerization or depolymerization
E
Spliceosome
Coronavirus disease - COVID-19
Ribosome
Bacterial invasion of epithelial cells
Nucleocytoplasmic transport
Oxidative phosphorylation
Cardiac muscle contraction
Glycolysis / Gluconeogenesis
Citrate cycle (TCA cycle)
Calcium signaling pathway
Pyruvate metabolism
F
Oxidative phosphorylation
Cardiac muscle contraction
Glycolysis / Gluconeogenesis
Citrate cycle (TCA cycle)
Pyruvate metabolism
Coronavirus disease - COVID-19
Ribosome
Spliceosome
Nucleocytoplasmic transport
Bacterial invasion of epithelial cells
G
H
Running Enrichment Score
EPITHELIAL MESENCHYMAL TRANSITION
G2M CHECKPOINT
KRAS SIGNALING DOWN
MYOGENESIS
OXIDATIVE PHOSPHORYLATION
PI3K AKT MTOR SIGNALING
Ranked List Metric
Rank in Ordered Dataset

Supplementary Fig. S10 Mass spectrometry analysis complements RNA-seq results in demonstrating simultaneous repression of malignant phenotypes and improvement of muscle integrity in sh-miR-treated FN-RMS tumor-bearing mice. Multi-level proteomic analyses are shown for the Healthy vs. UT comparison (**A**,**C**,**E**,**G**) and the UT vs. sh-miR1 comparison (**B**,**D**,**F**,**H**). **A**,**B** Volcano plots of DAPs. **C-H** Selected enrichment terms of (**C**,**D)** GO, (**E**,**F)** KEGG, and (**G**,**H**) GSEA based on DAPs. Bars indicate -$\log_{10}$ (adjusted p-value) for selected terms and pathways: positive values indicate upregulated while negative values indicate downregulated terms and pathways. Color gradient represents the magnitude of statistical significance. For GSEA, positive NES indicates enrichment among proteins upregulated in the second group relative to the first. Full GSEA statistics are provided in Table S5.

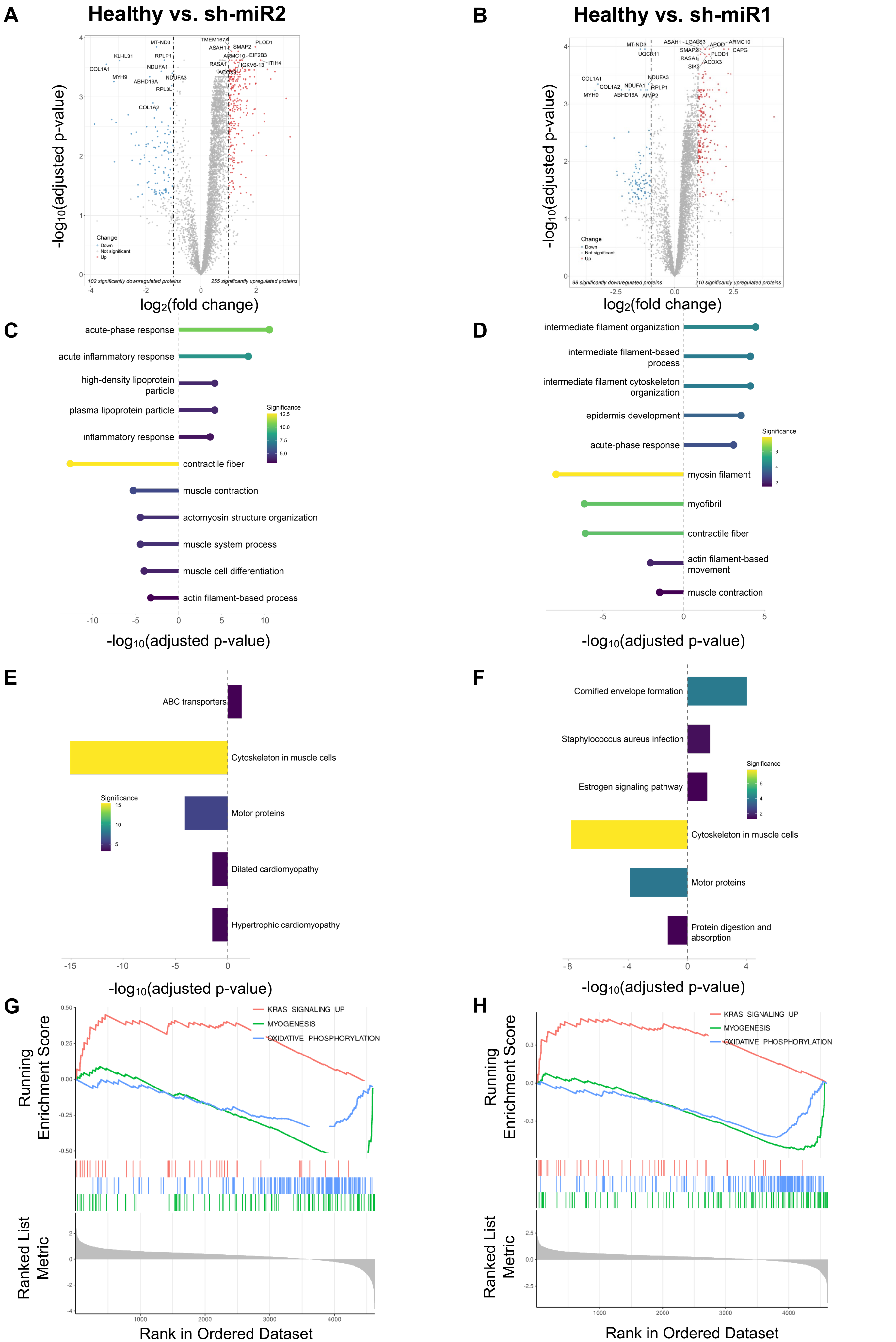

A
Healthy vs. sh-miR2
B
Healthy vs. sh-miR1
-log10(adjusted p-value)
log2(fold change)
102 significantly downregulated proteins
255 significantly upregulated proteins
98 significantly downregulated proteins
210 significantly upregulated proteins
Change
Down
Not significant
Up
C
acute-phase response
acute inflammatory response
high-density lipoprotein particle
plasma lipoprotein particle
inflammatory response
contractile fiber
muscle contraction
actomyosin structure organization
muscle system process
muscle cell differentiation
actin filament-based process
Significance
D
intermediate filament organization
intermediate filament-based process
intermediate filament cytoskeleton organization
epidermis development
acute-phase response
myosin filament
myofibril
contractile fiber
actin filament-based movement
muscle contraction
E
ABC transporters
Cytoskeleton in muscle cells
Motor proteins
Dilated cardiomyopathy
Hypertrophic cardiomyopathy
F
Cornified envelope formation
Staphylococcus aureus infection
Estrogen signaling pathway
Cytoskeleton in muscle cells
Motor proteins
Protein digestion and absorption
G
H
Running Enrichment Score
KRAS_SIGNALING_UP
MYOGENESIS
OXIDATIVE_PHOSPHORYLATION
Ranked List Metric
Rank in Ordered Dataset

Supplementary Fig. S11 Mass spectrometry analysis complements RNA-seq results in demonstrating similarities between healthy control and sh-miR-treated FN-RMS tumor-bearing mice. Multi-level proteomic analyses are shown for the Healthy vs. sh-miR2 comparison (**A**,**C**,**E**,**G**) and the Healthy vs. sh-miR1 comparison (**B**,**D**,**F**,**H**). **A**,**B** Volcano plots of DAPs. **C-H** Selected enrichment terms of **(C**,**D)** GO, **(E**,**F)** KEGG, and (**G**,**H**) GSEA based on DAPs. Bars indicate -$\log_{10}$ (adjusted p-value) for selected terms and pathways: positive values indicate upregulated while negative values indicate downregulated terms and pathways. Color gradient represents the magnitude of statistical significance. For GSEA, positive NES indicates enrichment among proteins upregulated in sh-miR1/2 relative to Healthy. Full GSEA statistics are provided in Table S5.

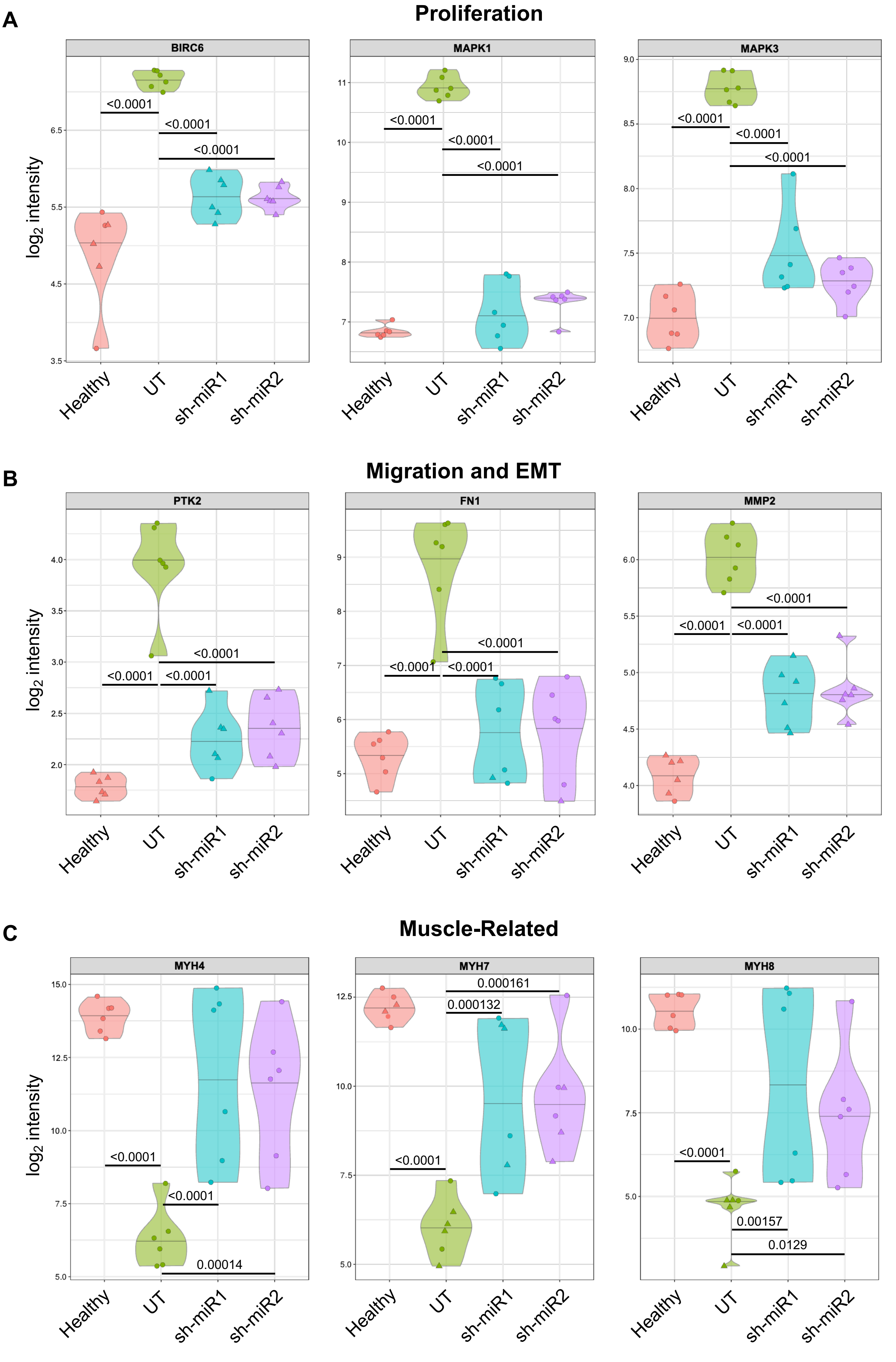
A
Proliferation
BIRC6
MAPK1
MAPK3
B
Migration and EMT
PTK2
FN1
MMP2
C
Muscle-Related
MYH4
MYH7
MYH8
log2 intensity
Healthy
UT
sh-miR1
sh-miR2
<0.0001
0.000161
0.000132
0.00014
0.00157
0.0129

Supplementary Fig. S12 Mass spectrometry analysis reveals significant alterations in markers associated with cell proliferative and migratory capacities and muscle phenotype upon MICAL2 silencing in FN-RMS tumor-bearing mice. A,B,C Violin plots showing differential protein abundances for (**A**) proliferation markers: BIRC6, MAPK1, MAPK3; (**B**) migration and EMT-associated markers: PTK2, FN1, MMP2; and (**C**) muscle-related markers: MYH4, MYH7, MYH8. Statistical significance is indicated by adjusted p-values.

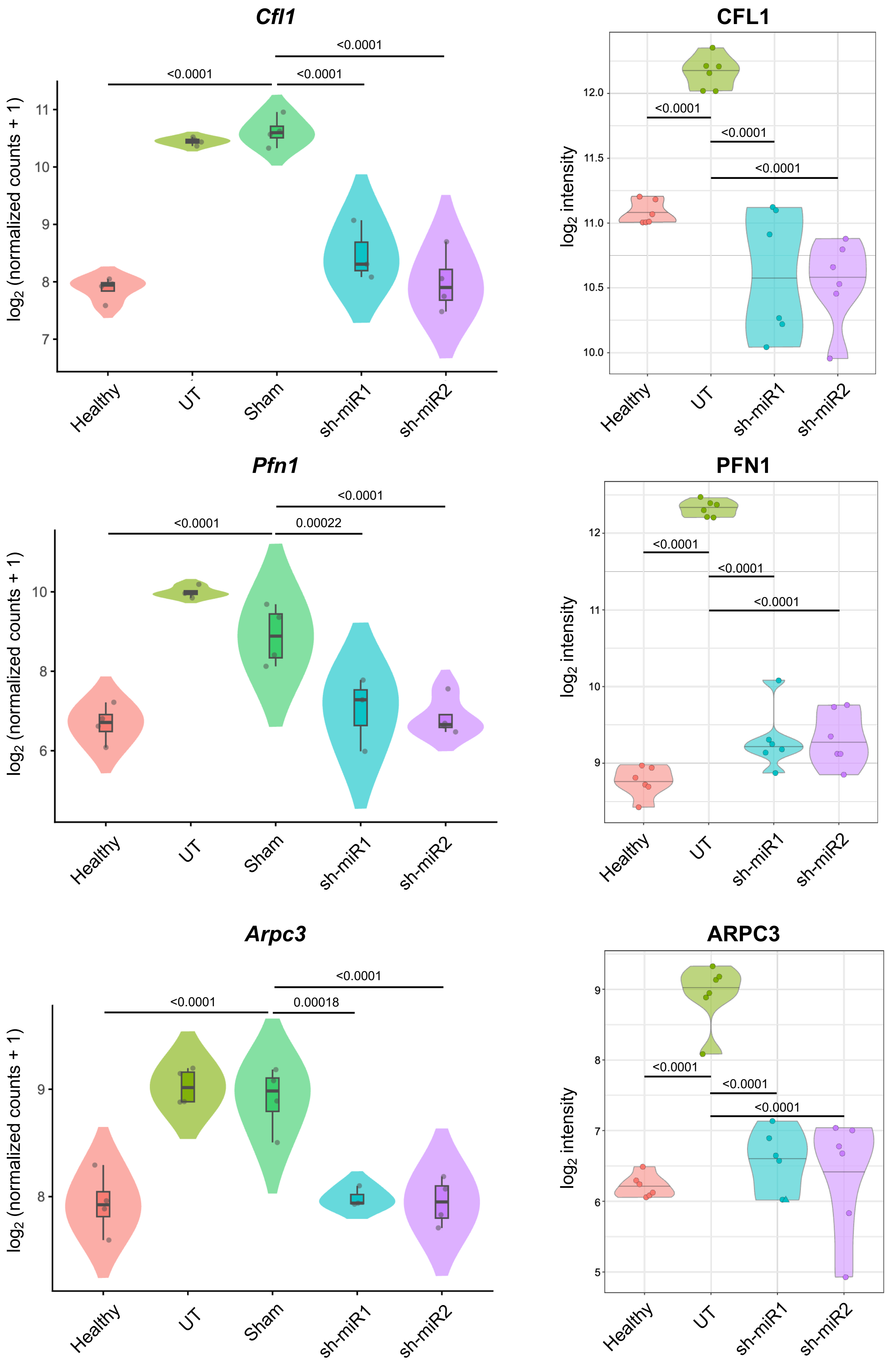

Cfl1
CFL1
Pfn1
PFN1
Arpc3
ARPC3
log2 (normalized counts + 1)
log2 intensity
<0.0001
0.00022
0.00018
Healthy
UT
Sham
sh-miR1
sh-miR2

Supplementary Fig. S13 Transcriptomic and proteomic analyses reveal concordant expression patterns of actin remodeling regulators. CFL1, PFN1, and ARPC3 expression levels were assessed in healthy, “non-treated,” and sh-miR-treated tumor-bearing mice. Data are presented as violin plots showing RNA (log_2[normalized counts + 1]) and protein (log_2 intensity) levels, and statistical significance is indicated by adjusted p-values.

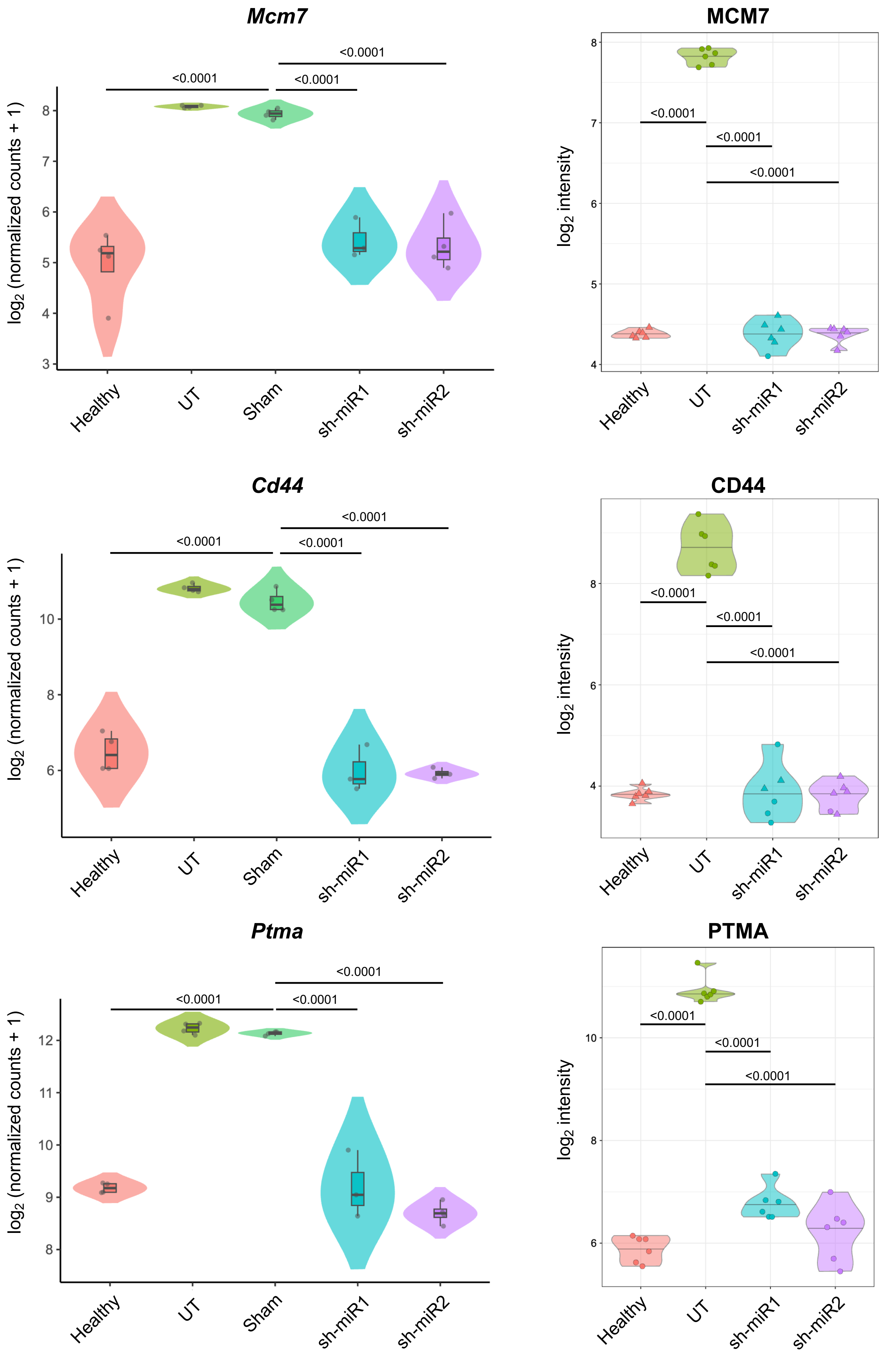

Mcm7
log2 (normalized counts + 1)
3
4
5
6
7
8
<0.0001
<0.0001
<0.0001
Healthy
UT
Sham
sh-miR1
sh-miR2
MCM7
log2 intensity
4
5
6
7
8
<0.0001
<0.0001
<0.0001
Healthy
UT
sh-miR1
sh-miR2
Cd44
log2 (normalized counts + 1)
6
8
10
<0.0001
<0.0001
<0.0001
Healthy
UT
Sham
sh-miR1
sh-miR2
CD44
log2 intensity
4
6
8
<0.0001
<0.0001
<0.0001
Healthy
UT
sh-miR1
sh-miR2
Ptma
log2 (normalized counts + 1)
8
9
10
11
12
<0.0001
<0.0001
<0.0001
Healthy
UT
Sham
sh-miR1
sh-miR2
PTMA
log2 intensity
6
8
10
<0.0001
<0.0001
<0.0001
Healthy
UT
sh-miR1
sh-miR2

Supplementary Fig. S14 Transcriptomic and proteomic analyses reveal concordant expression patterns of tumor growth and invasion markers. MCM7, CD44, and PTMA expression levels were assessed in healthy, “non-treated,” and sh-miR-treated tumor-bearing mice. Data are presented as violin plots showing RNA (log_2[normalized counts + 1]) and protein (log_2 intensity) levels, and statistical significance is indicated by adjusted p-values.

Supplementary Table S1. Complete table of significance values for XTT assays of KMR19 (Fig. 1D) and KMR46 (Fig. S2C) based on two-way ANOVA with Dunnett's multiple comparisons test.

**KMR19**	**Relative Absorbance (450 nm)**	**0h**	**9h**	**24h**	**48h**	**72h**
	Sham vs. Lipo C-	>0.9999	0.7881	<0.0001	<0.0001	<0.0001
	Sham vs. sh-miR1	0.9048	0.0746	<0.0001	<0.0001	<0.0001
	Sham vs. sh-miR2	0.9496	0.1353	0.0019	<0.0001	<0.0001
	Sham vs. sh-miR3	0.9909	0.3669	<0.0001	<0.0001	<0.0001
**KMR46**	**Relative Absorbance (450 nm)**	**0h**	**9h**	**24h**	**48h**	**72h**
	Sham vs. sh-miR1	0.9305	<0.0001	<0.0001	<0.0001	<0.0001
	Sham vs. sh-miR2	0.8215	<0.0001	<0.0001	<0.0001	<0.0001
	Sham vs. sh-miR3	0.0773	<0.0001	<0.0001	<0.0001	<0.0001
**KMR19**	**Relative Absorbance (450 nm)**	**Lipo C-**	**Sham**	**sh-miR1**	**sh-miR2**	**sh-miR3**
	0h vs. 9h	0.0441	0.2553	0.8863	0.852	0.7502
	0h vs. 24h	<0.0001	<0.0001	0.9117	0.0112	0.2145
	0h vs. 48h	<0.0001	<0.0001	0.0063	0.0057	0.2752
	0h vs. 72h	<0.0001	<0.0001	0.027	<0.0001	<0.0001
	9h vs. 24h	<0.0001	0.0003	>0.9999	0.0456	0.7862
	9h vs. 48h	<0.0001	<0.0001	0.0186	0.0223	0.8675
	9h vs. 72h	<0.0001	<0.0001	0.0889	<0.0001	<0.0001
	24h vs. 48h	<0.0001	<0.0001	0.0145	0.999	0.9998
	24h vs. 72h	<0.0001	<0.0001	0.0726	0.0015	0.0025
	48h vs. 72h	<0.0001	<0.0001	0.9765	0.0037	0.0014
**KMR46**	**Relative Absorbance (450 nm)**	**Sham**	**sh-miR1**	**sh-miR2**	**sh-miR3**	
	0h vs. 9h	0.0029	<0.0001	<0.0001	<0.0001	
	0h vs. 24h	<0.0001	<0.0001	<0.0001	<0.0001	
	0h vs. 48h	<0.0001	<0.0001	<0.0001	<0.0001	
	0h vs. 72h	<0.0001	<0.0001	<0.0001	<0.0001	
	9h vs. 24h	<0.0001	<0.0001	<0.0001	0.0025	
	9h vs. 48h	<0.0001	<0.0001	<0.0001	<0.0001	
	9h vs. 72h	<0.0001	<0.0001	<0.0001	<0.0001	
	24h vs. 48h	<0.0001	0.9556	0.1674	0.4173	
	24h vs. 72h	0.1084	0.9887	0.3959	0.5105	
	48h vs. 72h	0.1203	0.9995	0.9882	0.9999	

Supplementary Table S2. Complete table of significance values for XTT assays of transduced KMR19 cell lines (Fig. S4) based on two-way repeated measures ANOVA with Šídák's multiple comparisons test.

**Relative Absorbance (490 nm)**	**FLuc+ vs. FLuc+ w/ doxy**	**Sham vs. Sham w/ doxy**	**sh-miR1 vs. sh-miR1 w/ doxy**	**sh-miR2 vs. sh-miR2 w/ doxy**
**0h**	0.9987	0.7658	0.6846	>0.9999
**24h**	0.9973	>0.9999	0.9997	<0.0001
**48h**	0.6687	0.9985	0.0189	<0.0001
**72h**	0.7904	0.0531	0.0056	<0.0001
**96h**	0.9949	0.3391	0.1636	<0.0001
**120h**	0.7519	>0.9999	0.9222	0.1601

Supplementary Table S3. Complete tables of significance values for all time points of *in vivo* BLI experiments for primary tumor (Fig. 4B) and lung metastasis (Fig. 4D) based on two-way ANOVA with Tukey's multiple comparisons test.

Primary Tumor BLI total flux (photons/sec)	Day 2	Day 5	Day 7	Day 9	Day 11	Day 13	Day 15	Day 17
Healthy vs. UT	>0.9999	>0.9999	>0.9999	>0.9999	0.9922	0.8607	0.5316	0.001
Healthy vs. Sham	>0.9999	>0.9999	>0.9999	0.9993	0.9902	0.1926	0.0037	0.0001
Healthy vs. sh-miR1	>0.9999	>0.9999	>0.9999	>0.9999	>0.9999	>0.9999	0.9992	0.9661
Healthy vs. sh-miR2	>0.9999	>0.9999	>0.9999	>0.9999	>0.9999	>0.9999	>0.9999	>0.9999
UT vs. Sham	>0.9999	>0.9999	>0.9999	0.9998	>0.9999	0.762	0.2636	0.9856
UT vs. sh-miR1	>0.9999	>0.9999	>0.9999	>0.9999	0.994	0.9044	0.688	0.0109
UT vs. sh-miR2	>0.9999	>0.9999	>0.9999	>0.9999	0.9942	0.8899	0.6028	0.0014
Sham vs. sh-miR1	>0.9999	>0.9999	>0.9999	0.9996	0.9924	0.2384	0.0086	0.0017
Sham vs. sh-miR2	>0.9999	>0.9999	>0.9999	0.9994	0.9925	0.2214	0.0055	0.0002
sh-miR1 vs. sh-miR2	>0.9999	>0.9999	>0.9999	>0.9999	>0.9999	>0.9999	>0.9999	0.9803

Primary Tumor BLI total flux (photons/sec)	Day 19	Day 21	Day 23	Day 25
Healthy vs. UT	<0.0001	<0.0001	<0.0001	<0.0001
Healthy vs. Sham	<0.0001	<0.0001	<0.0001	<0.0001
Healthy vs. sh-miR1	0.2513	0.019	0.3748	0.1894
Healthy vs. sh-miR2	0.9982	0.9991	0.9997	0.9995
UT vs. Sham	0.9938	<0.0001	0.957	0.0381
UT vs. sh-miR1	0.0105	<0.0001	<0.0001	<0.0001
UT vs. sh-miR2	<0.0001	<0.0001	<0.0001	<0.0001
Sham vs. sh-miR1	0.0375	0.0443	<0.0001	<0.0001
Sham vs. sh-miR2	<0.0001	<0.0001	<0.0001	<0.0001
sh-miR1 vs. sh-miR2	0.4148	0.0405	0.4873	0.2893

Source of Variation	% of total variation	p-value
Interaction	21.24	<0.0001
Row Factor (time)	19.65	<0.0001
Column Factor (group)	17.31	<0.0001

Lung Metastasis BLI total flux (photons/sec)	Day 5	Day 11	Day 15	Day 17	Day 19	Day 21	Day 23	Day 25
Healthy vs. UT	>0.9999	>0.9999	0.9999	0.2671	0.3313	<0.0001	<0.0001	<0.0001
Healthy vs. Sham	>0.9999	>0.9999	0.958	0.7548	0.2715	0.069	<0.0001	<0.0001
Healthy vs. sh-miR1	>0.9999	>0.9999	>0.9999	>0.9999	>0.9999	0.9705	0.9152	0.6567
Healthy vs. sh-miR2	>0.9999	>0.9999	>0.9999	>0.9999	>0.9999	>0.9999	>0.9999	>0.9999
UT vs. Sham	>0.9999	>0.9999	0.9828	0.9268	>0.9999	<0.0001	0.9156	0.8442
UT vs. sh-miR1	>0.9999	>0.9999	>0.9999	0.2675	0.3983	<0.0001	<0.0001	<0.0001
UT vs. sh-miR2	>0.9999	>0.9999	>0.9999	0.2587	0.3331	<0.0001	<0.0001	<0.0001
Sham vs. sh-miR1	>0.9999	>0.9999	0.9657	0.7554	0.3322	0.2693	<0.0001	<0.0001
Sham vs. sh-miR2	>0.9999	>0.9999	0.961	0.7446	0.2732	0.0907	<0.0001	<0.0001
sh-miR1 vs. sh-miR2	>0.9999	>0.9999	>0.9999	>0.9999	>0.9999	0.9856	0.9376	0.714

Source of Variation	% of total variation	p-value
Interaction	27.28	<0.0001
Row Factor (time)	20.47	<0.0001
Column Factor (group)	24.51	<0.0001

Supplementary Table S4. Significance values for in vivo treadmill exhaustion tests (Figs. 5A and S7A) based on two-way ANOVA with Tukey's multiple comparisons test.

Time (s)	Day 0	Day 5	Day 10	Day 15	Day 20	Day 25
Healthy vs. UT	0.3874	0.9535	0.0003	<0.0001	<0.0001	<0.0001
Healthy vs. Sham	0.822	0.0167	<0.0001	<0.0001	<0.0001	<0.0001
Healthy vs. sh-miR1	0.8722	0.3141	0.3291	0.0007	<0.0001	<0.0001
Healthy vs. sh-miR2	>0.9999	0.6106	0.7751	0.0008	0.0003	<0.0001
UT vs. Sham	0.0878	0.0036	0.0427	0.7679	0.9863	0.9498
UT vs. sh-miR1	0.119	0.423	0.0019	0.0003	0.0001	<0.0001
UT vs. sh-miR2	0.3173	0.9186	<0.0001	0.0004	0.0017	0.0006
Sham vs. sh-miR1	>0.9999	0.0378	0.0004	0.0003	0.0002	<0.0001
Sham vs. sh-miR2	0.7705	0.0011	<0.0001	0.0003	0.0018	0.0005
sh-miR1 vs. sh-miR2	0.8318	0.5631	0.6304	>0.9999	0.7741	0.798
**Distance (m)**	**Day 0**	**Day 5**	**Day 10**	**Day 15**	**Day 20**	**Day 25**
Healthy vs. UT	0.584	0.9412	0.0003	0.0006	<0.0001	<0.0001
Healthy vs. Sham	0.8036	0.0185	<0.0001	0.0006	<0.0001	<0.0001
Healthy vs. sh-miR1	0.8483	0.3099	0.3146	0.0019	<0.0001	<0.0001
Healthy vs. sh-miR2	0.9998	0.6262	0.7736	0.0021	0.0002	<0.0001
UT vs. Sham	0.0618	0.0031	0.064	0.7737	0.9712	0.8975
UT vs. sh-miR1	0.0849	0.4187	0.0031	0.0044	0.0017	0.0013
UT vs. sh-miR2	0.2258	0.9226	<0.0001	0.0043	0.0206	0.0064
Sham vs. sh-miR1	>0.9999	0.0378	0.0016	0.0041	0.0017	0.0012
Sham vs. sh-miR2	0.7708	0.0012	<0.0001	0.004	0.0203	0.0063
sh-miR1 vs. sh-miR2	0.8318	0.5526	0.5943	>0.9999	0.7611	0.8503
**Work (J)**	**Day 0**	**Day 5**	**Day 10**	**Day 15**	**Day 20**	**Day 25**
Healthy vs. UT	0.4949	0.9901	0.0185	0.0024	0.0008	0.0005
Healthy vs. Sham	0.992	0.1635	0.0026	0.0024	0.0008	0.0005
Healthy vs. sh-miR1	0.8882	0.4122	0.8349	0.0176	0.003	0.0015
Healthy vs. sh-miR2	0.9927	0.9157	0.9811	0.0212	0.0037	0.0014
UT vs. Sham	0.4822	0.0269	0.1196	0.723	0.9095	0.9841
UT vs. sh-miR1	0.1811	0.4993	0.0006	0.0019	0.0005	0.0001
UT vs. sh-miR2	0.9377	0.9833	0.004	0.0031	0.003	0.001
Sham vs. sh-miR1	0.998	0.0085	<0.0001	0.0018	0.0005	0.0001
Sham vs. sh-miR2	0.9554	0.0486	0.0006	0.003	0.0029	0.001
sh-miR1 vs. sh-miR2	0.8317	0.8694	0.9878	0.9952	0.6356	0.5782
**Power (W)**	**Day 0**	**Day 5**	**Day 10**	**Day 15**	**Day 20**	**Day 25**
Healthy vs. UT	0.9843	0.999	0.5623	0.0002	<0.0001	0.0001
Healthy vs. Sham	0.9993	0.9998	0.0029	0.0002	0.0001	<0.0001
Healthy vs. sh-miR1	0.9998	0.9994	0.9916	0.1551	0.0135	0.0049
Healthy vs. sh-miR2	0.9962	0.9974	0.9996	0.4775	0.0378	0.0106
UT vs. Sham	0.9992	0.9953	0.1257	0.8364	0.9554	0.9998
UT vs. sh-miR1	0.9729	0.9929	0.7571	0.0003	<0.0001	<0.0001
UT vs. sh-miR2	>0.9999	>0.9999	0.5724	0.0029	<0.0001	<0.0001
Sham vs. sh-miR1	0.9967	>0.9999	0.0049	0.0003	<0.0001	<0.0001
Sham vs. sh-miR2	>0.9999	0.9921	0.0115	0.0026	<0.0001	<0.0001
sh-miR1 vs. sh-miR2	0.9907	0.9889	0.9775	0.9894	0.8478	0.853

Supplementary Table S5. GSEA results for selected hallmark gene sets across all pairwise comparisons included in proteomics analysis (Figs. 7F, S10G,H, and S11G,H). For each comparison, only statistics for the gene sets highlighted in the corresponding figures are shown.

Comparison	Gene Set	Set Size	NES	p-value	p.adjust	q-value
**Healthy vs. UT**	Oxidative Phosphorylation	164	-3.39330901461	1E-10	1.56666666666e-09	8.0701754385e-10
**Healthy vs. UT**	Myogenesis	105	-3.27298867709	1E-10	1.56666666666e-09	8.0701754385e-10
**Healthy vs. UT**	KRAS Signaling Down	19	-2.12051418642	0.0003398415428	0.001774728057001	0.0009141936239
**Healthy vs. UT**	Epithelial Mesenchymal Transition	95	1.59864521648	0.0040443644814	0.013577509330486	0.0069940137648
**Healthy vs. UT**	PI3K-AKT-mTOR Signaling	53	1.62754832219	0.0035569095237	0.012859595970494	0.0066242039713
**Healthy vs. UT**	G2M Checkpoint	91	2.00099054000	2.4014764269e-06	1.61241988668e-05	8.3058583188e-06
**Healthy vs. sh-miR1**	Myogenesis	105	-2.61625881646	1e-10	4.7e-09	3.3684210526e-09
**Healthy vs. sh-miR1**	Oxidative Phosphorylation	164	-2.23496636072	9.4468546883e-10	2.22001085176e-08	1.5910492106e-08
**Healthy vs. sh-miR1**	KRAS Signaling Up	42	1.75718222004	0.0025719098875	0.015109970589147	0.0108290942632
**Healthy vs. sh-miR2**	Myogenesis	105	-2.54758129188	1.1698119873e-10	5.49811634047e-09	4.1866955336e-09
**Healthy vs. sh-miR2**	Oxidative Phosphorylation	164	-1.73092004575	3.5863242373e-05	0.000561857463854	0.0004278421897
**Healthy vs. sh-miR2**	KRAS Signaling Up	42	1.62267892473	0.0077876042960	0.040668600212962	0.0309682510020
**UT vs. sh-miR1**	G2M Checkpoint	91	-2.03158734786	3.6437232460e-06	2.85424987606e-05	1.7898991384e-05
**UT vs. sh-miR1**	PI3K-AKT-mTOR Signaling	53	-1.61130308007	0.0064224603996	0.021561117055912	0.0135209692623
**UT vs. sh-miR1**	Epithelial Mesenchymal Transition	95	-1.55639329687	0.0041609130277	0.017778446573274	0.0111488578734
**UT vs. sh-miR1**	KRAS Signaling Down	19	2.13726165564	0.0001446706972	0.000849940346631	0.0005329973058
**UT vs. sh-miR1**	Myogenesis	105	2.94682872258	1e-10	1.56666666666e-09	9.8245614035e-10
**UT vs. sh-miR1**	Oxidative Phosphorylation	164	3.00711393393	1e-10	1.56666666666e-09	9.8245614035e-10
**UT vs. sh-miR2**	G2M Checkpoint	91	-2.08835924853	5.0392958176e-07	3.94744839047e-06	2.4754435595e-06
**UT vs. sh-miR2**	PI3K-AKT-mTOR Signaling	53	-1.62120930841	0.0051189683345	0.020049292643771	0.0125729046814
**UT vs. sh-miR2**	Epithelial Mesenchymal Transition	95	-1.52045467629	0.0069536135902	0.025139987595536	0.0157652777754
**UT vs. sh-miR2**	KRAS Signaling Down	19	1.91321374058	0.0015577710983	0.008135026847050	0.0051014726028
**UT vs. sh-miR2**	Myogenesis	105	2.99046492703	1e-10	1.56666666666e-09	9.8245614035e-10
**UT vs. sh-miR2**	Oxidative Phosphorylation	164	3.02848222742	1e-10	1.56666666666e-09	9.8245614035e-10

Supplementary Table S6. WB primary antibodies and dilutions.

Primary antibody	Dilution
rabbit anti-MICAL2 (OriGene TA331975)	1:500
rabbit anti-MICAL2 (Proteintech 13965-1-AP)	1:1000
mouse anti-α-Tubulin (Sigma T5168)	1:1000
mouse anti-p-ERK (Santa Cruz sc-7383)	1:300
rabbit anti-total-ERK (Santa Cruz sc-271269)	1:300
mouse anti-p-FAK (Sigma Aldrich 05-1140)	1:500
rabbit anti-total-FAK (Sigma Aldrich 06-543)	1:500
goat anti-vascular endothelial cadherin (VEC) (R&D Systems AF1002)	1:500
mouse anti-MCM7 (Santa Cruz sc-9966)	1:1000
