# Supplementary material for: Unraveling the dual roles of MICAL2 in skeletal muscle physiology and fusion negative rhabdomyosarcoma tumor progression
Source: Cell Death Dis. 2026 Jul 7;17(1):845. doi: 10.1038/s41419-026-09029-w (PMC13623957; doi:10.1038/s41419-026-09029-w)

Original Blots

Fig. 2A

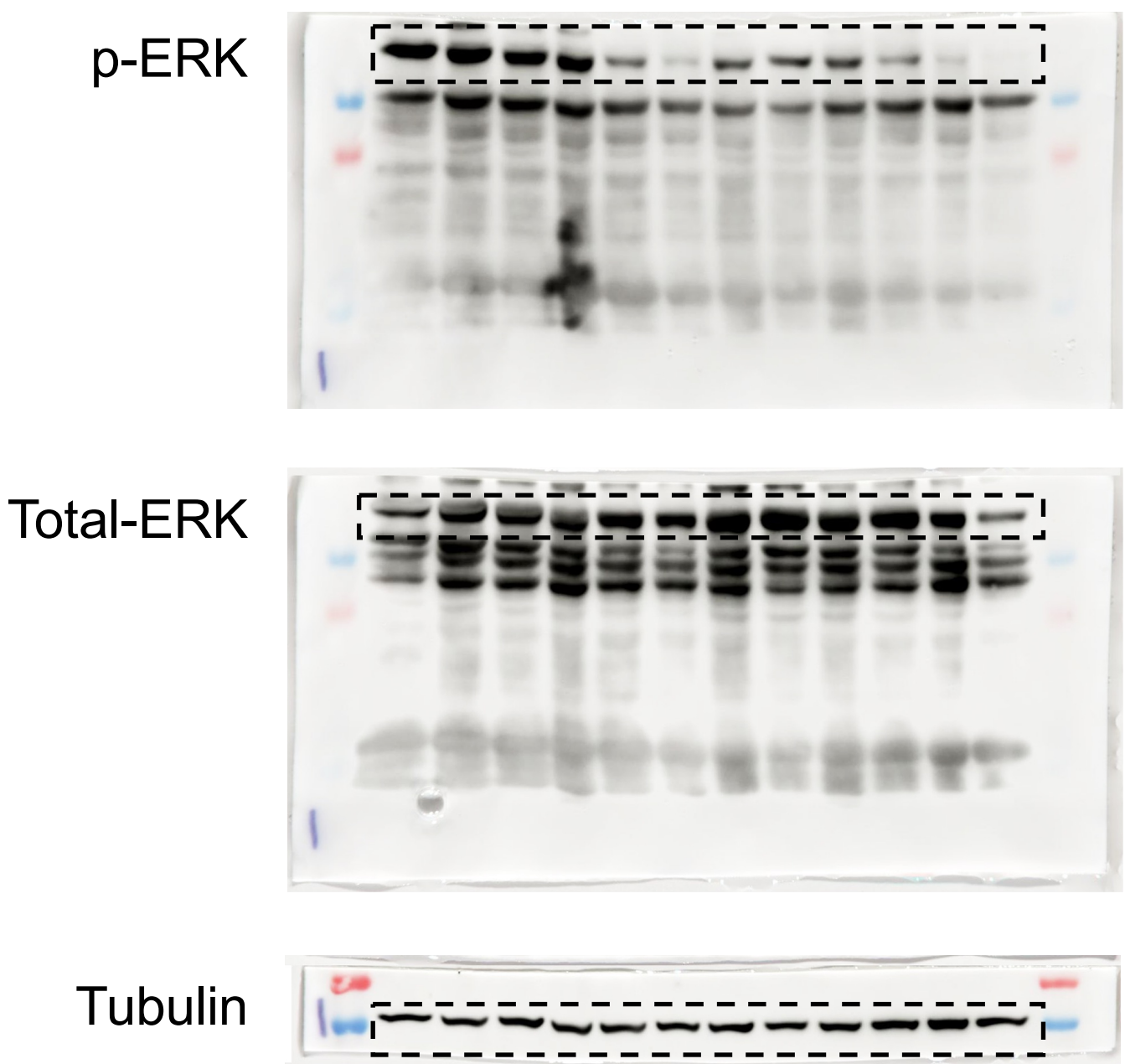

Fig. 2B

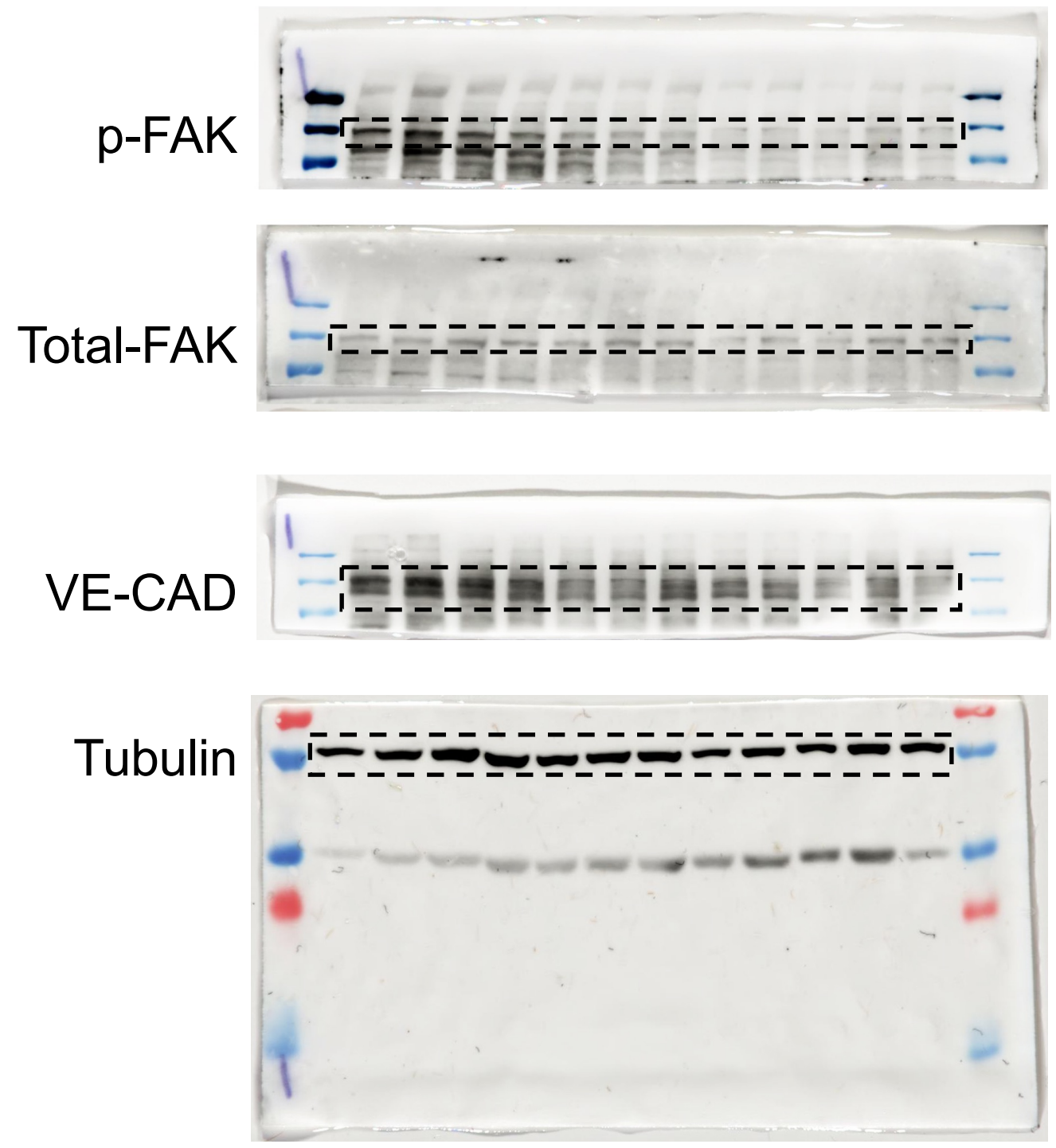

Fig. 2D

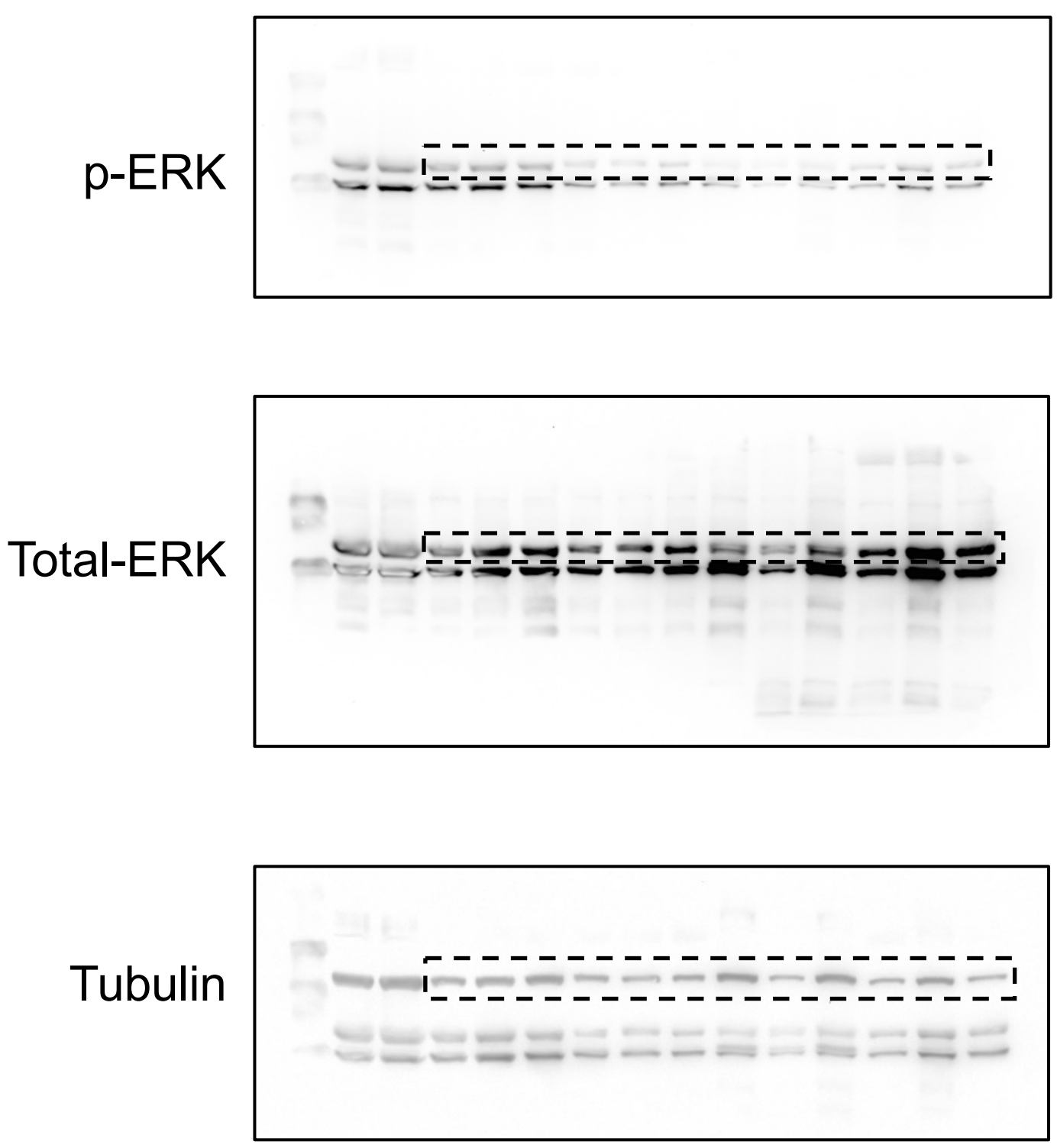

Fig. 2E

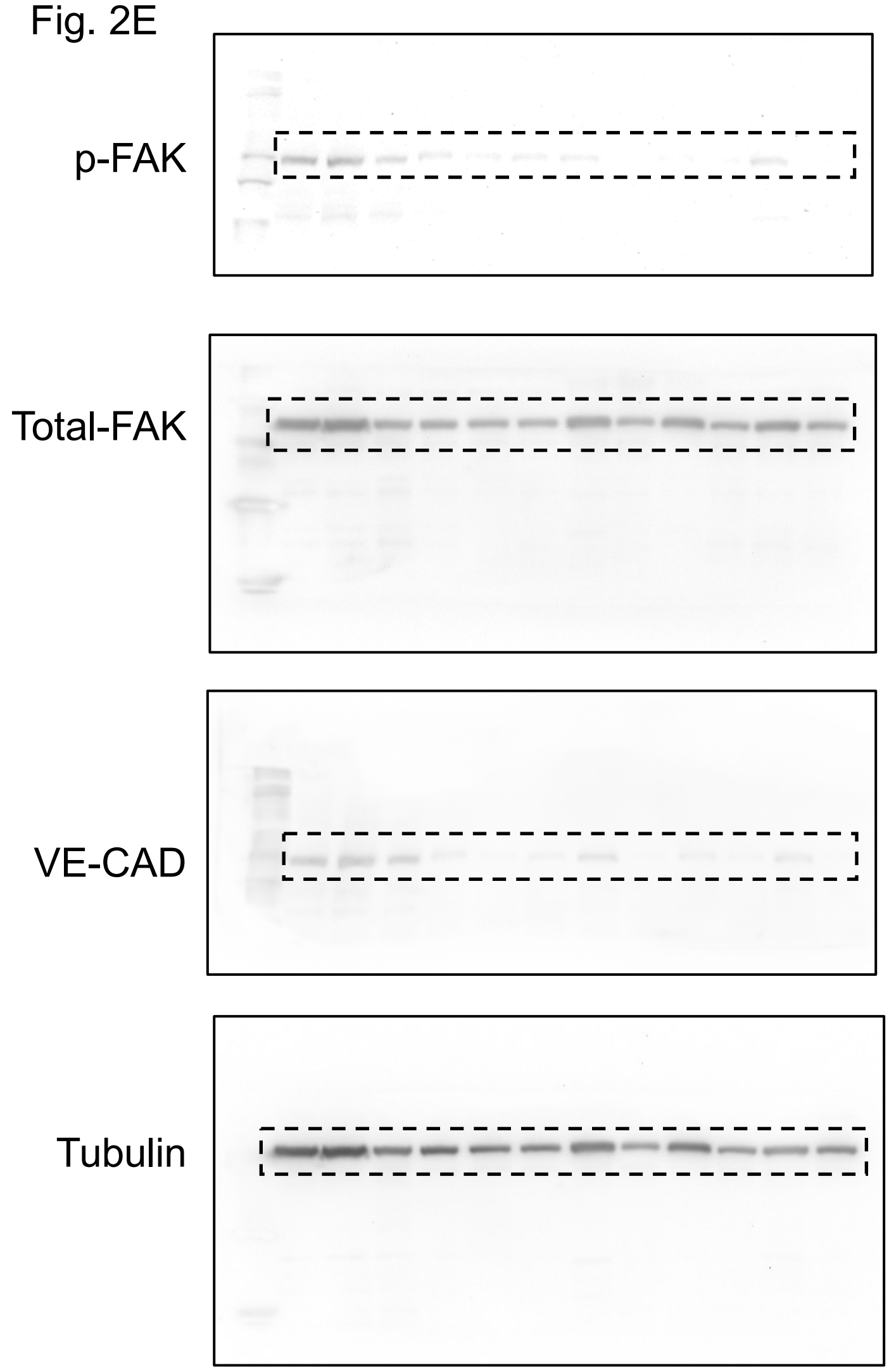

Fig. 3A

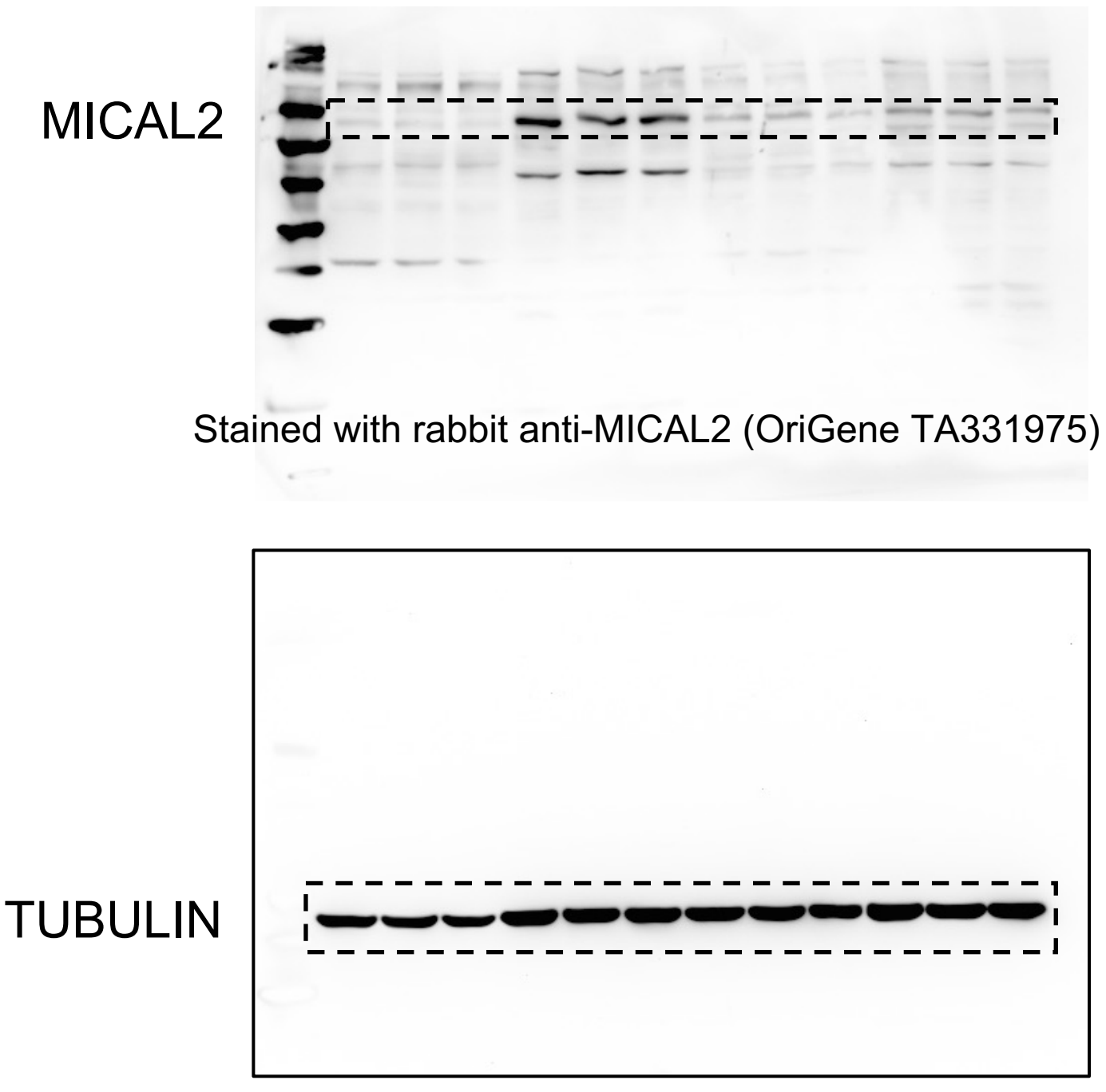
MICAL2
Stained with rabbit anti-MICAL2 (OriGene TA331975)
TUBULIN

Fig. 3E

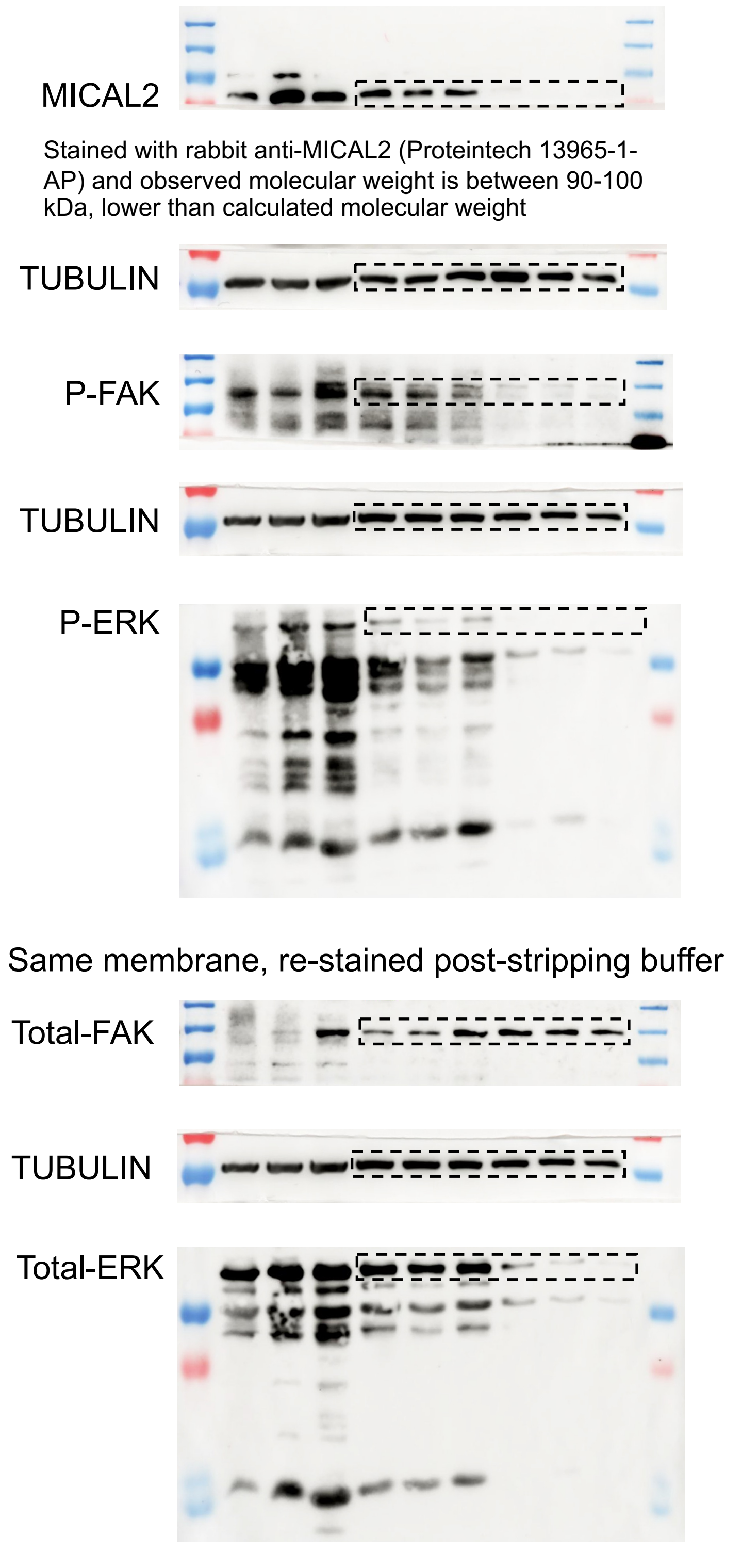
MICAL2
Stained with rabbit anti-MICAL2 (Proteintech 13965-1-AP) and observed molecular weight is between 90-100 kDa, lower than calculated molecular weight
TUBULIN
P-FAK
TUBULIN
P-ERK
Same membrane, re-stained post-stripping buffer
Total-FAK
TUBULIN
Total-ERK

Fig. 5C

MICAL2

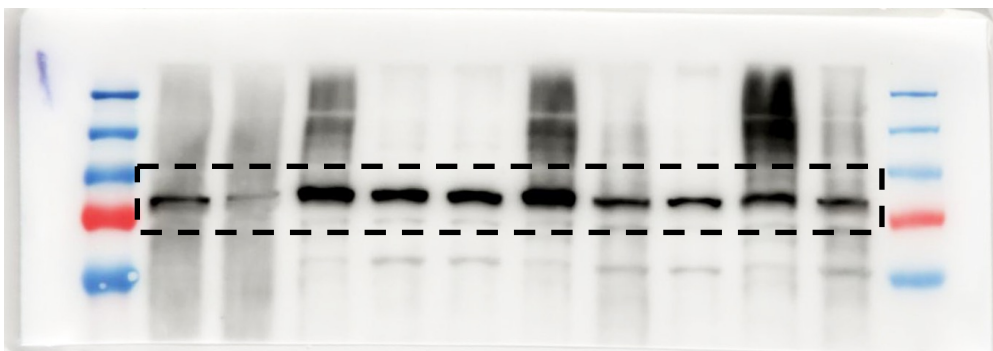

Stained with rabbit anti-MICAL2 (Proteintech 13965-1-AP)

GAPDH

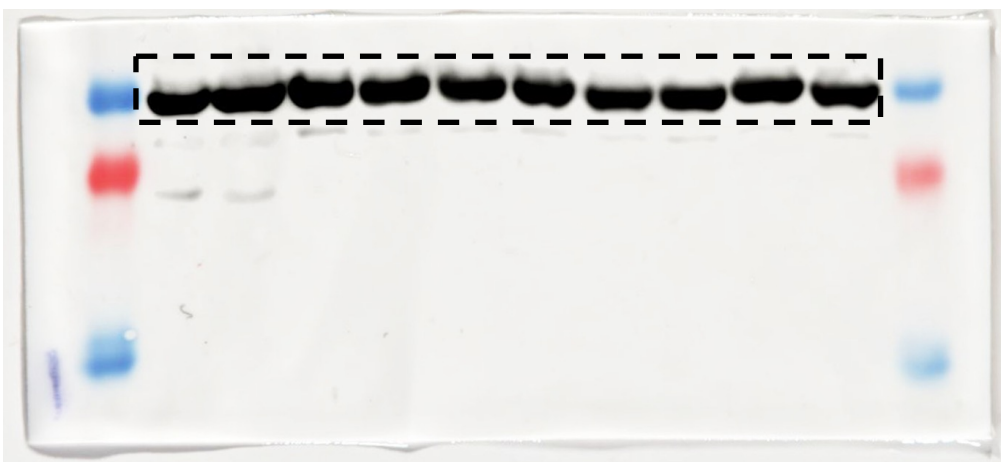

(used for quantification, not included in figure)

MCM7

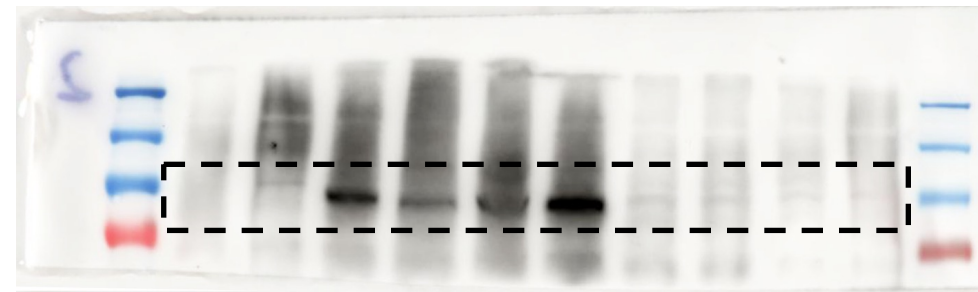

GAPDH

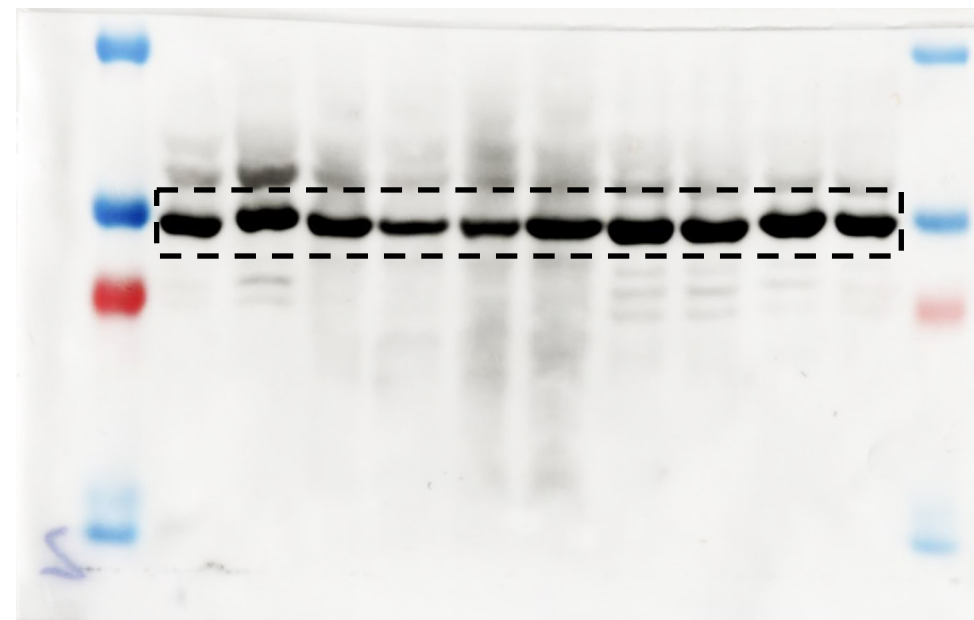

Fig. S1B

MICAL2

TUBULIN

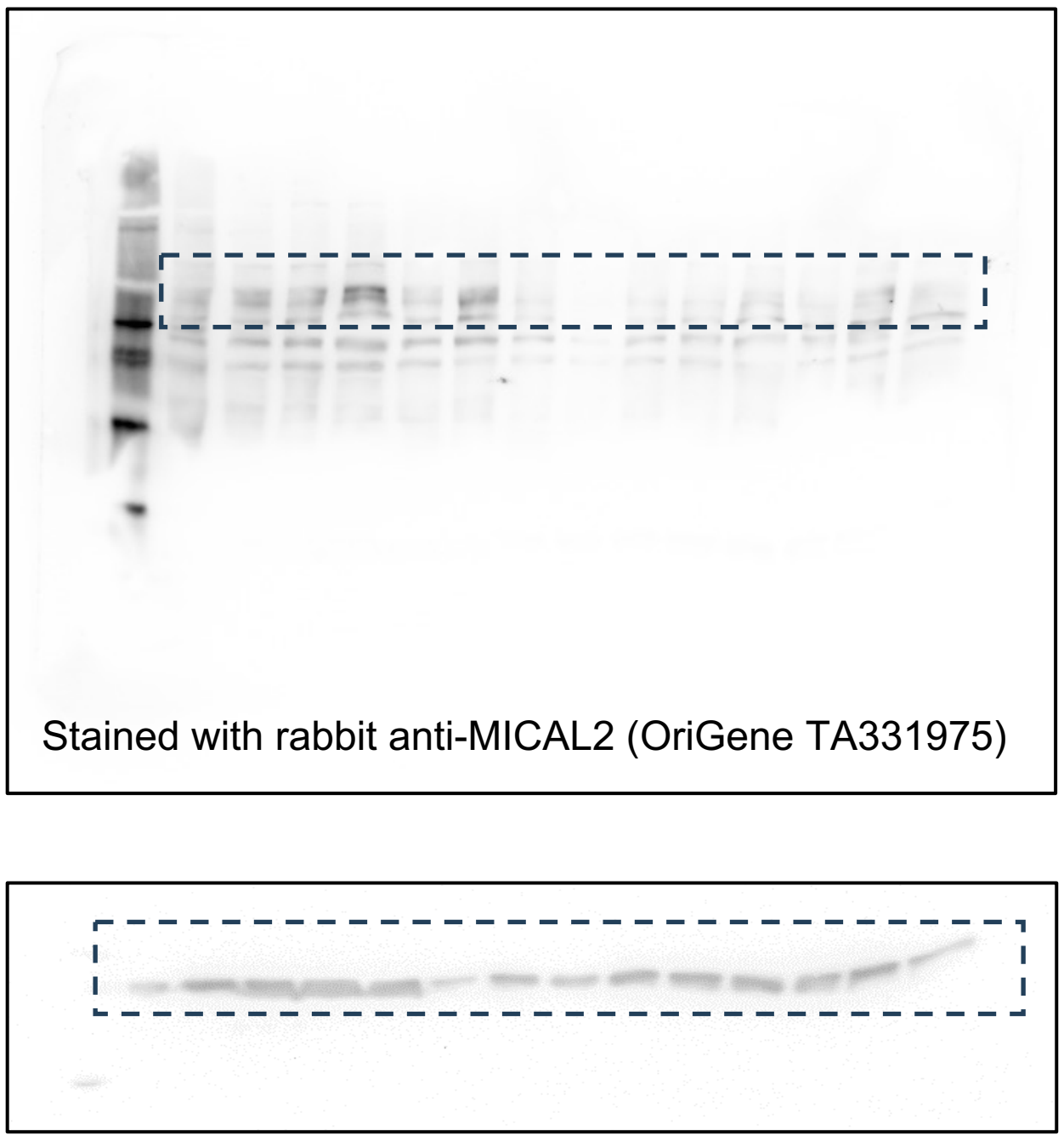

Fig. S1D

MICAL2

TUBULIN

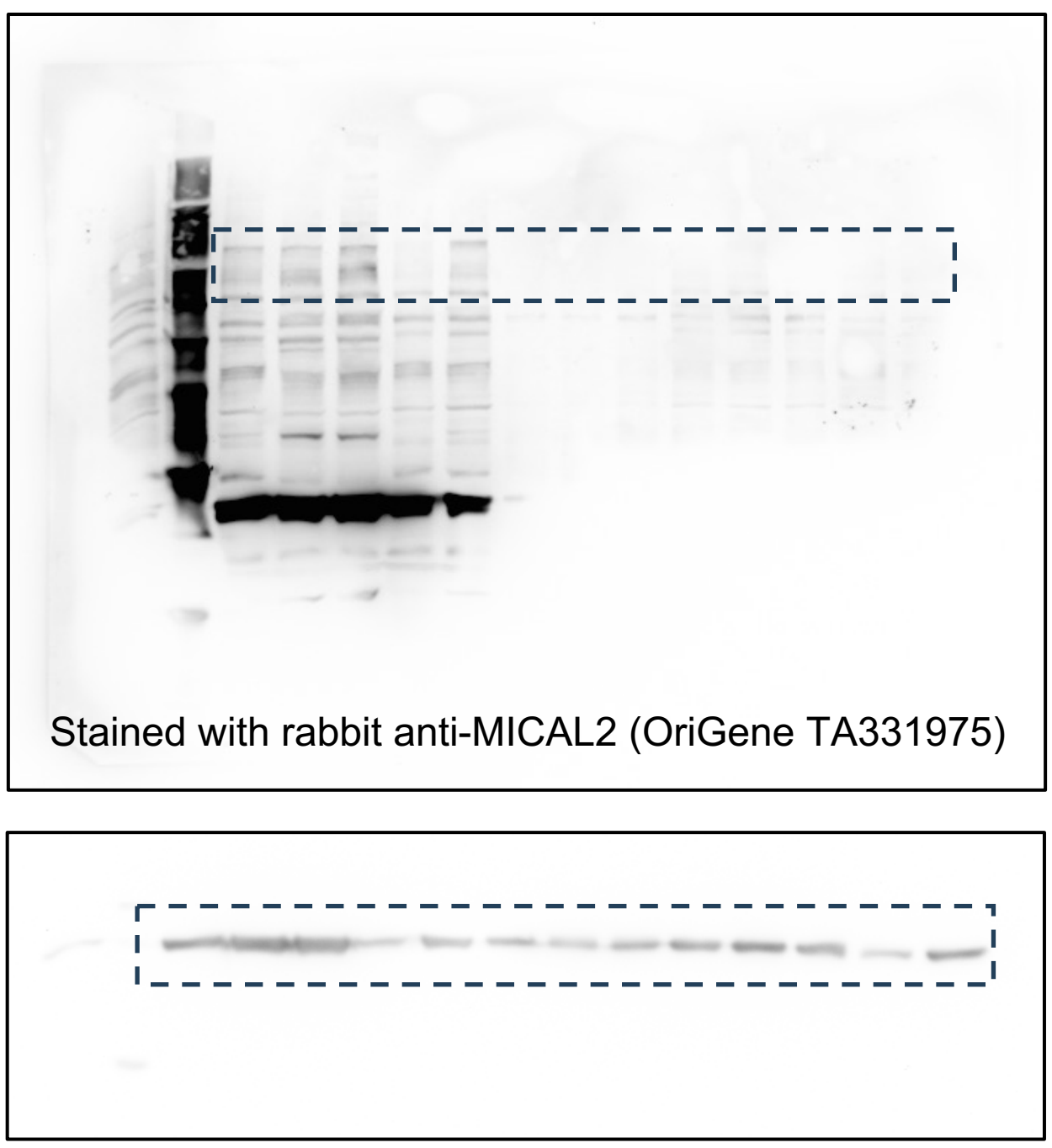

Supplement: Supplementary file 2 [file 41419_2026_9029_MOESM2_ESM.pdf]
